# greenPipes: an integrated data analysis pipeline for greenCUT&RUN and CUT&RUN genome-localization datasets

**DOI:** 10.1093/bioinformatics/btae307

**Published:** 2024-05-08

**Authors:** Sheikh Nizamuddin, H T Marc Timmers

**Affiliations:** Department of Urology, Medical Center-University of Freiburg, Freiburg, 79016, Germany; German Cancer Consortium (DKTK), partnersite Freiburg, a partnership between the DKFZ and Medical Center-University of Freiburg, Germany; Department of Urology, Medical Center-University of Freiburg, Freiburg, 79016, Germany; German Cancer Consortium (DKTK), partnersite Freiburg, a partnership between the DKFZ and Medical Center-University of Freiburg, Germany

## Abstract

**Motivation:**

To study gene regulation through transcription factors and chromatin modifiers, a variety of genome-wide techniques are used. Recently, CUT&RUN-based technologies have become popular, but a pipeline for the comprehensive analysis of CUT&RUN datasets is currently lacking. Here, we present the “greenPipes” package, which includes fine-tuned parameters specifically for bioinformatic analyses of greenCUT&RUN and CUT&RUN datasets. greenPipes provides additional functionalities for data analysis and data integration with other -omics technologies, which are either not available in other pipelines developed for CUT&RUN datasets or scattered in the literature as individual packages.

**Availability and implementation:**

Source code and a manual of the greenPipes are freely available on GitHub website (https://github.com/snizam001/greenPipes). The test datasets, comprehensive annotation files, and other datasets are available at https://osf.io/ruhj9/.

**Contact:**

n.sheikh@dkfz-heidelberg.de or m.timmers@dkfz-heidelberg.de

**Supplementary information:**

The handbook of greenPipes is available online at *Bioinformatics* as Supplementary text.

## 1 Introduction

Studying gene regulation in diverse species or model systems involves a primary focus on comprehending the mechanisms by which transcription factors and chromatin regulatory proteins operate. To understand these complex processes, a variety of *in vitro* and *in vivo* DNA-binding assays are used including genome-wide localization methods like ChIPseq, CUT&RUN, and CUT&Tag. These methods generate large datasets, which need to be compared with datasets originating from a variety of omics-technologies to obtain a better understanding of gene regulation at a global scale.

The CUT&RUN (cleavage under target and release using nuclease) technique is relatively new and was developed to profile genome-wide binding events of transcription factors, chromatin regulators, and histone modifications using low numbers of cells or nuclei under native conditions (Skene and Henikoff 2017). As far as we know, there are currently two primary pipelines for analyzing CUT&RUN data: CUT&RUNTools (versions 1 and 2) and the cutandrun tool of nf-core (Zhu *et al.* 2019, Ewels *et al.* 2020, Yu *et al.* 2021). However, these tools lack a comprehensive analysis beyond read alignment and peak calling, and they do not facilitate the comparison of CUT&RUN datasets with other -omics datasets.

To address these limitations, we have developed the “greenPipes” pipeline. Specifically tailored for the datasets generated by greenCUT&RUN and CUT&RUN, it incorporates spike-in normalization methods at different stage of analysis. With greenPipes, users can perform all analyses in a single run, and it offers additional features for data analysis along with seamless integration with other -omics technologies, such as targeted quantitative mass spectrometry. Many of the features found in greenPipes are absent in existing tools or are scattered across the literature as separate packages rendering greenPipes a valuable and user-friendly tool for researchers in this field. The distinctions of greenPipes compared to currently available pipelines are succinctly outlined in Supplementary Table 1.

## 2 Implementation

The greenPipes pipeline is built on the Python, R, and Perl languages. Besides its own functions written in R and Python, the pipeline depends on other tools including Bowtie2, HOMER, and deepTools. The greenPipes uses mamba for creating a conda environment for hassle-free and faster installation of greenPipes. Installation of Perl modules is facilitated through cpanm, while R packages are obtained from CRAN and the Bioconductor repository. greenPipes is designed to take advantage of multicore processors and supports multi-threading for faster data processing. It also provides the convenience of downloading a basic database for the species of interest of the user directly within the pipeline. This feature streamlines the setup process and ensures that users have the essential reference data for analyses. The other datasets, e.g. reference genome index files and additional annotation datasets, can be downloaded from https://osf.io/ruhj9/.

**Figure 1.**
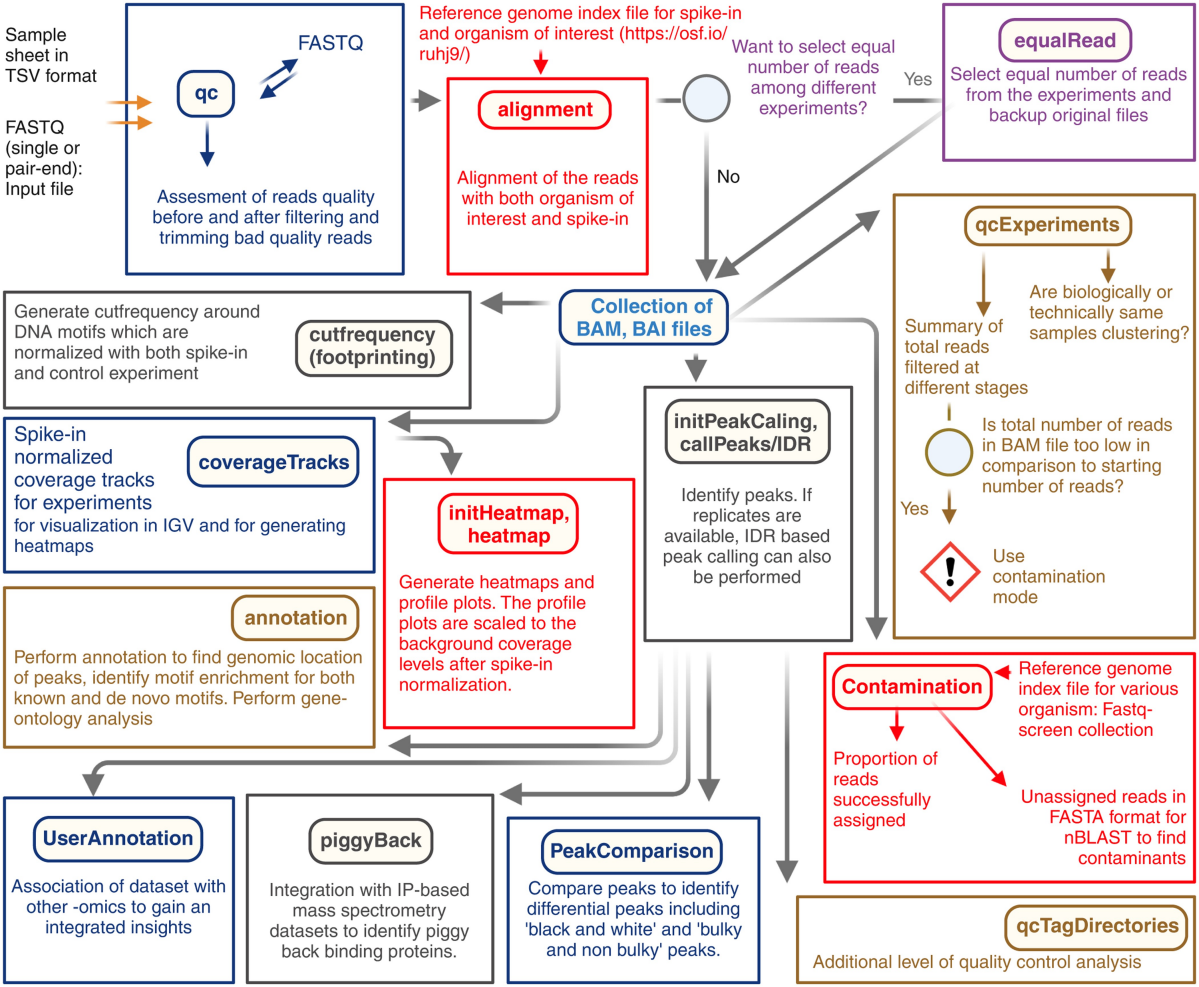
Visual representation of the workflow of the greenPipes pipeline. This pipeline only needs two experiment-specific input files and it run most of the analyses in a single command as shown in the workflow.

A comprehensive manual outlining the workflow and various parameters is available on the GitHub page and as a Supplementary file, and summarized in Fig. 1. The greenPipes pipeline offers extensive customization through numerous parameter settings. All these options are explained in the integrated help section. The program is designed to operate through the command line interface, featuring a user-friendly design that facilitates quick familiarity for users, including those who may not be experts in the field. In the event of a failure, the greenPipes pipeline allows users to re-enter and resume the process, promoting efficient and time-saving data processing.

The pipeline incorporates optimized parameters, coupled with insightful suggestions to guide users toward an optimal data analysis. For a detailed understanding of parameter choices and recommendations, users are encouraged to refer to the manual accompanying the greenPipes pipeline. The developers of techniques like CUT&RUN and similar methods endorse the use of spike-in normalization to mitigate technical variations in datasets (Skene and Henikoff 2017, Nizamuddin *et al.* 2021). The spike-in refers to heterologous DNA, either synthetic or derived from MNase-digested *Saccharomyces cerevisiae* or *Drosophila melanogaster* chromatin, or from carry-over *Escherichia coli* DNA. The greenPipes pipeline incorporates spike-in normalization in various modes when comparing different samples, such as (i) peak calling, (ii) differential peak calling, (iii) read coverage calculation, and (iv) heatmap generation. This allows users to initiate the analysis from any step, even if they did not normalize the dataset with spike-in DNA earlier. In cases where users intend to use carry-over *E. coli* DNA as a spike-in (Meers *et al.* 2019), we recommend assessing the correlation of carry-over *E. coli* DNA with add-on spike-in controls. Correlations may vary across laboratory setups, especially when employing highly purified preparations of MNase fusion proteins. Notably, controls typically exhibit a higher spike-in proportion compared to experiments due to the low background of CUT&RUN technologies. greenPipes issues a warning if spike-in levels in controls surpass those in experiments and provides recommended parameters for data analysis. We suggest employing control read depth coverage at a ratio of one-third or one-fourth compared to the experiment in the greenCUT&RUN technique. To address lower control sequencing depth, the normalization formula now includes the total number of reads. The formula has been modified so that if the coverage depth is identical in both control and experimental samples, it will be transformed into the spike-in normalization formula used by Nizamuddin *et al.* (2021).

Unlike conventional pipelines, greenPipes also identifies those peaks that exhibit variations not based on reads coverage, but are influenced by the length of the reads. These peaks are categorized as “Bulky” and “NonBulky” to capture this additional dimension. From a biological perspective, these peaks could be significant owing to distinct complex formations occurring at these loci. Additionally, the pipeline detects unique peaks within an experiment. To achieve this, it conducts simulations to determine the maximum background coverage achievable in a given experiment. Subsequently, when a genomic location exhibits four times or more reads coverage in another experiment, it is classified as a unique or “black and white” peak. To the best of our knowledge, this classification of peaks does not currently exist in any other pipeline. In addition to peak calling algorithms applicable to the individual experiments, Irreproducibility Discovery Rate (IDR)-based peak calling has also been implemented in greenPipes. This facilitates the utilization of IDR when users have experimental replicates, offering independence from varying significance peak calling criteria across labs and experiments. This implemented functionality is also not present in other pipelines.

It is a common practice to compare genome-wide binding profile datasets with other types of data, such as proteomics, transcriptomics, and various genomic techniques, to achieve a comprehensive understanding of the underlying molecular mechanisms. In contrast to other pipelines, greenPipes uniquely allows the comparison of these diverse datasets. By integrating affinity-based mass spectrometry with greenCUT&RUN and CUT&RUN datasets, greenPipes identifies piggy-back binding proteins. However, it is essential to acknowledge a limitation in this aspect. Identification of piggy-back-binding proteins is limited to those with known DNA motifs. greenPipes comes with comprehensive datasets designed for peak annotation. This includes enhancer–gene interaction datasets spanning 110 tissues/cell-lines, developed by Gao and Qian (2020). Additionally, it offers 8165 annotations covering enhancers and super-enhancers, genomic locations, DNase-I hypersensitive sites, predicted and measured histone modifications, transcription factor binding sites, and other protein-binding locations (Kichaev *et al.* 2017). greenPipes is designed to generate a unified integrated output, accommodating datasets from diverse sources, including user-provided annotation datasets as well.

## 3 Conclusion

Our work introduces the greenPipes pipeline as a powerful and user-friendly tool tailored for the analysis of greenCUT&RUN and CUT&RUN datasets and addressing limitations in currently available pipelines. The greenPipes pipeline stands out by offering comprehensive features, including multi-threading support, optimized parameter settings, and integration with other omics technologies, such as targeted quantitative mass spectrometry. Unlike current pipelines, greenPipes goes beyond conventional read coverage-based analysis, providing a unique classification of peaks influenced by read length. This classification, along with the detection of piggy-back-binding proteins and the comparison of diverse datasets, sets greenPipes apart as a versatile and inclusive analytical tool. In summary, greenPipes is a valuable addition to the repertoire of bioinformatic tools available for the analysis of gene regulation datasets, providing a holistic approach to understanding the complex mechanisms underlying transcription factors, chromatin regulators, and histone modifications.

## Supplementary Material

btae307_Supplementary_Data

## Data Availability

No new data were generated or analysed in support of this research.
