# Supplementary material for: greenPipes: an integrated data analysis pipeline for greenCUT&RUN and CUT&RUN genome-localization datasets
Source: Bioinformatics. 2024 May 8;40(5):btae307. doi: 10.1093/bioinformatics/btae307 (PMC11112040; doi:10.1093/bioinformatics/btae307)
Supplement: btae307_Supplementary_Data [file btae307_supplementary_data.zip › Supplementary table revised.docx]

**Supplementary table 1**: Comparison of greenPipes pipeline with available pipelines

| Criteria | Sub-criteria | Availability in the different pipelines | | | |
| --- | --- | --- | --- | --- | --- |
|  |  | greenPipes | CUT&RUNTools v1.0 | CUT&RUNTools v2.0 | cutandrun (nf-core) |
| URL | - | https://github.com/snizam001/greenPipe | https://bitbucket.org/qzhudfci/cutruntools/src/master/ | https://github.com/fl-yu/CUT-RUNTools-2.0 | https://github.com/nf-core/cutandrun |
| Support for bulk seq dataset | - | Available | Available | Available | Available |
| Support for single cell dataset | - | Not available | Not available | Available | Not available |
| QC of FASTQ files | - | Available | Available | Available | Available |
| Merging of re-sequenced FastQ files | - | Not available | Not available | Not available | Available |
| Alignment | Support for single-end reads | Available | Not available | Not available | Not available |
|  | Support for pair-end reads | Available | Available | Available | Available |
|  | Alignment post-processing *i.e.* duplicate removal | Not available | Not available | Not available | Available |
| Option to select equal number of reads | - | Available | Not available | Not available | Not available |
| QC of the Experiment | - | Available | Not available | Not available | Available |
| Contamination | - |  |  |  |  |
| Peak calling | Peak calling in the individual experiments: MACS2 | Not available | Available | Available | Available |
|  | Peak calling in the individual experiments: HOMER | Available | Not available | Not available | Not available |
|  | Peak calling in the individual experiments: SEACR | Available | Available | Available | Available |
|  | IDR based peak calling (Support for the experimental replicates) | Available | Not available | Not available | Not available |
| qc of the Tag directories | - | Available | Not available | Not available | Not available |
| Peak comparison | Common peaks | Available | Not available | Not available | Available |
|  | Differential peaks | Available | Not available | Not available | Not available |
|  | Unique peaks | Available | Not available | Not available | Not available |
|  | Bulky/Non-bulky peaks | Available | Not available | Not available | Not available |
| Footprinting | - | Available | Available | Available | Not available |
| Heatmaps | - | Available | Not available | Not available | Available |
| Coverage tracks | - | Available | Not available | Not available | Available |
| Annotations | Known motif finding | Available | Not available | Not available | Not available |
|  | *De novo* motif finding | Available | Available | Available | Not available |
|  | Annotation to the genomic features | Available | Not available | Not available | Not available |
|  | Gene ontology | Available | Not available | Not available | Not available |
| Integration with IP-based mass spectrometry datasets | - | Available | Not available | Not available | Not available |
| Integration with transcriptomics and other genomic techniques | - | Available | Not available | Not available | Not available |
