# Supplementary material for: greenPipes: an integrated data analysis pipeline for greenCUT&RUN and CUT&RUN genome-localization datasets
Source: Bioinformatics. 2024 May 8;40(5):btae307. doi: 10.1093/bioinformatics/btae307 (PMC11112040; doi:10.1093/bioinformatics/btae307)
Supplement: btae307_Supplementary_Data [file btae307_supplementary_data.zip › Manual-revised.pdf]

greenPipes: an automated data analysis platform for greenCUT&RUN and CUT&RUN genome-localization datasets

Sheikh Nizamuddin[1, 2] and H.T. Marc Timmers[1, 2, ‡]

[1]Department of Urology, Medical Center-University of Freiburg, 79016 Freiburg, Germany
[2]German Cancer Consortium (DKTK) partner site Freiburg, German Cancer Research Center (DKFZ), 69120 Heidelberg, Germany
[‡]**Corresponding author**: H.T. Marc Timmers, m.timmers@dkfz-heidelberg.de

Copyright © Sheikh Nizamuddin (n.sheikh@dkfz-heidelberg.de) and Marc Timmers (m.timmers@dkfz-heidelberg.de)

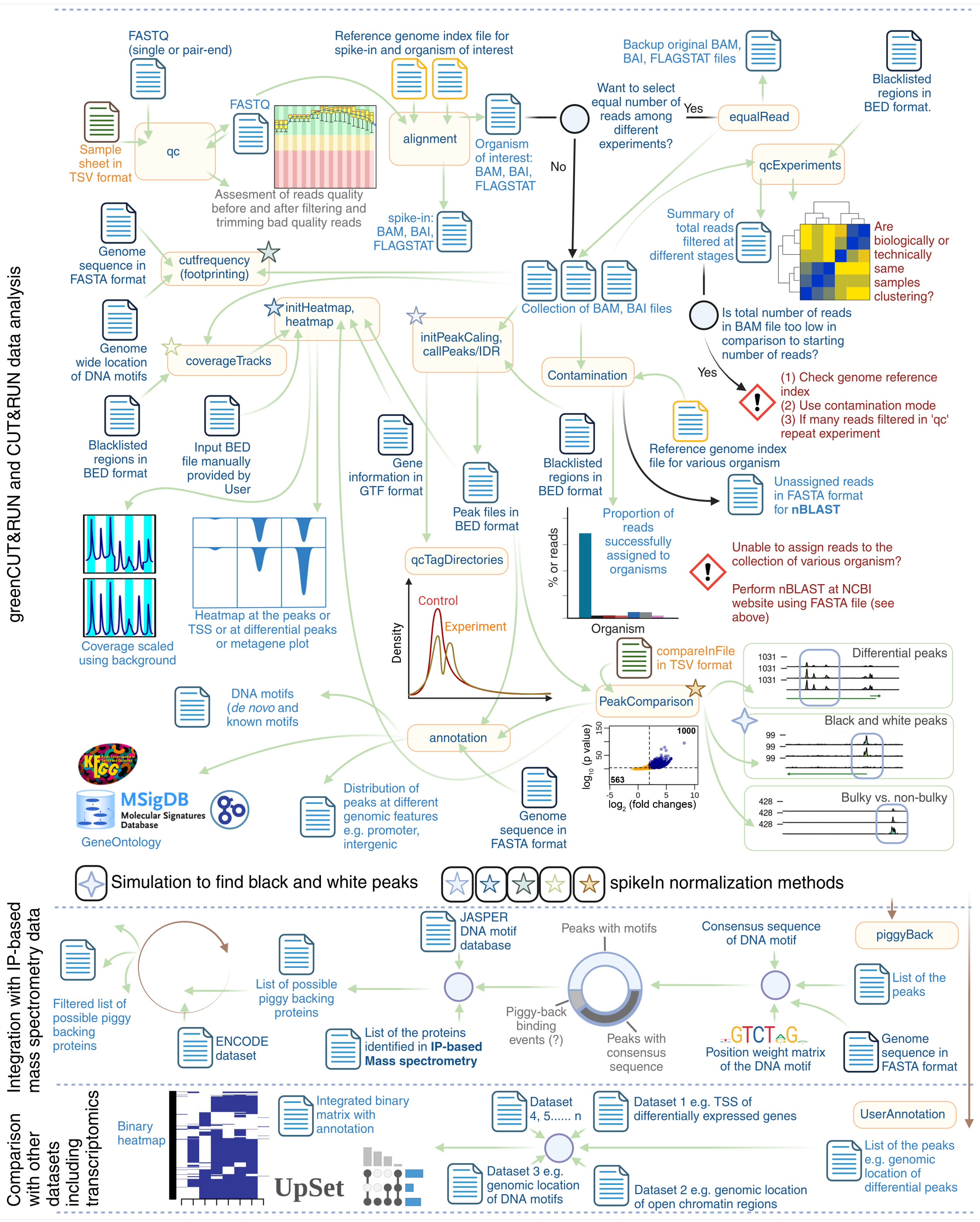

Figure 1: The workflow of greenPipes.

Contents

1 Introduction 1

2 Set up of greenPipes 2
2.1 Automatic installation of greenPipes and its dependencies . . . 3
2.2 Dependencies . . . 3
2.3 greenPipes: Datasets . . . 4
2.3.1 Genome index files and black listed regions . . . 4
2.3.2 Annotation dataset . . . 5
2.3.3 Test dataset . . . 5

3 Modes and options overview, experiment-specific input files in greenPipes, quick start and help 7
3.1 Modes and options . . . 7
3.2 Experiment specific files . . . 8
3.2.1 Sample sheet for experimental design . . . 8
3.2.2 Sheet for peak comparison among different experiments . . . 9
3.3 Quick start . . . 9
3.4 Getting help, error and log file . . . 10

4 Quality control (qc) 13

5 Alignment and selection of equal reads 15
5.1 alignment . . . 15
5.2 equalRead . . . 17

6 Quality control of the experiment 20
6.1 qcExperiment . . . 21
6.2 contamination . . . 22

7 Peak calling 25
7.1 initPeakCalling . . . 26
7.2 qcTagDirectories . . . 29
7.3 callPeaks . . . 31
7.4 idr . . . 33

8 Annotation 36
8.1 annotation . . . 36
8.2 UserAnnotation . . . 39

9 Comparison of peaks **44**
9.1 Differential peaks 45
9.2 Black and white 46
9.3 Bulky *vs.* nonBulky 47
9.4 Motifs, annotation and gene ontology 48
9.5 Options 48

10 Visualization of peaks **50**
10.1 Distribution of spike-in 50
10.2 coverageTracks 51

11 Footprinting **54**
11.1 cutfrequency 54

12 Heatmap **58**
12.1 initHeatmap 59
12.2 heatmap 59

13 Integration and comparison with other -omics datasets **65**
13.1 Integration with IP-based mass spectrometry 65
13.1.1 piggyBack 65
13.1.2 doughnut 69
13.2 Comparison with other -omics technology 70
13.2.1 Cis-elements/factors: Comparison with ATACseq and other omics technologies 70
13.2.2 Trans-elements/factors: Comparison with transcriptomics 70

14 Appendix **72**

Chapter 1

Introduction

The primary objective in many laboratories investigating gene regulation across various species or model systems is understanding the mechanisms of action of transcription factors and chromatic regulatory proteins. To achieve this, a range of *in vitro* and *in vivo* DNA binding assays, including genome-wide localization methods like ChIPseq, CUT&RUN, and CUT&Tag, are employed. These assays yield substantial datasets that must be integrated with information from various omics technologies such as GRO/PROseq, ATACseq, and bisulfite-sequencing for a comprehensive understanding. In 2021, we introduced the novel method `greenCUT&RUN` for genome-wide profiling of GFP-tagged proteins, known for its high sensitivity, resolution, accuracy, and reproducibility, ensuring specificity in mapping in vivo protein-DNA contacts[1]. This technique generates robust genomic profiles that are accurate and unbiased towards open chromatin, circumvating the dependency on protein-specific antibodies. The `greenPipes` pipeline is designed for the `greenCUT&RUN` technique, but it is also applicable to datasets obtained via CUT&RUN or other variations (e.g., CUT&Tag).

[1] Sheikh Nizamuddin, Stefanie Koidl, Tanja Bhuiyan, Tamara V Werner, Martin L Biniossek, Alexandre MJJ Bonvin, Silke Lassmann, HT Marc Timmers (2021). "Integrating quantitative proteomics with accurate genome profiling of transcription factors by greenCUT&RUN." Nucleic acids research 49(9): e49-e49.

Chapter 2

Set up of greenPipes

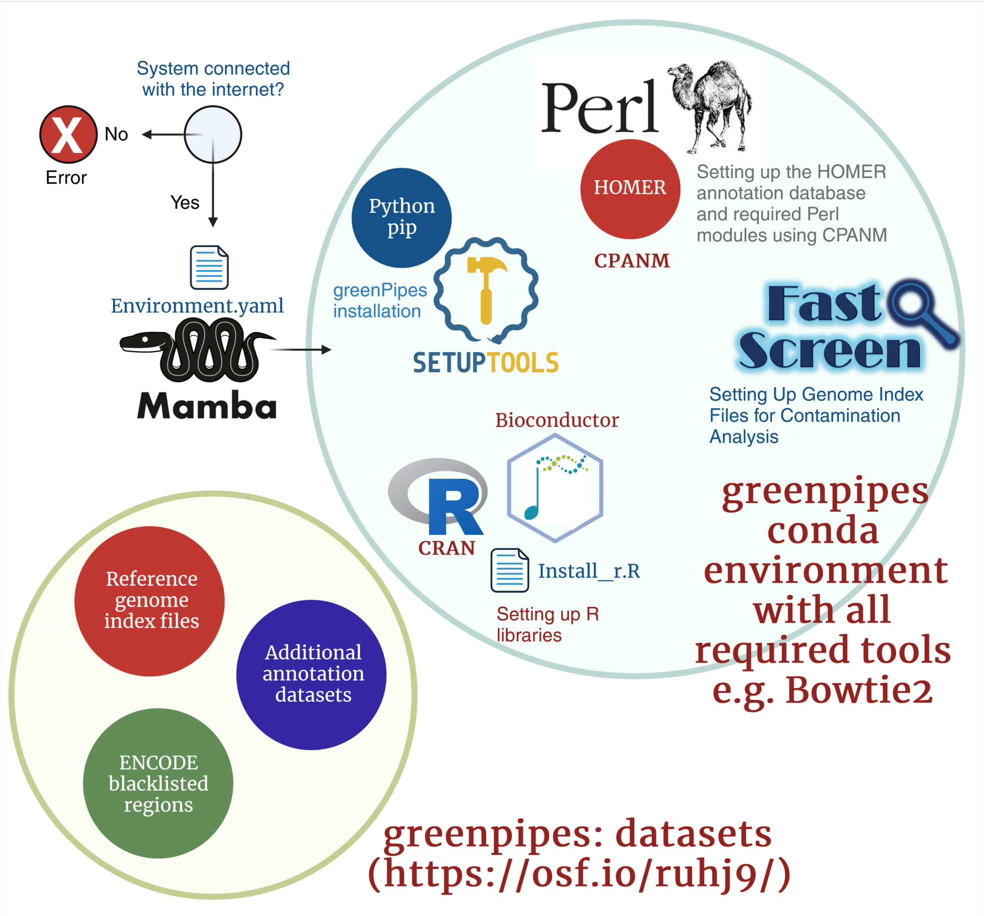

Figure 2.1: The installation of greenPipes involves the creation of a conda environment named "greenpipes". The user will install all the necessary dependencies, packages, and tools within this environment. Users have the option to download genome index files, additional annotation datasets, and ENCODE blacklisted regions from the 'greenPipes database' at https://osf.io/ruhj9/, or they can choose to use their own databases.

2.1 Automatic installation of greenPipes and its dependencies

greenPipes is a Python-based wrapper that utilizes its own functions written in Python, R, and Perl languages, as well as several external tools. Users can use `Install.sh` for the automatic installation. This file comes bundled with the greenPipes package. The setup process for greenPipes is outlined in detail in **Figure 2.1**.

For automatic installation, use shell script `Install.sh`, which is bundled with greenPipes pipeline. Mamba should be installed in the system. If not available, users can install it by installing `mambaforge` from https://github.com/conda-forge/miniforge for their operating system. Download greenPipes from the https://github.com/snizam001/greenPipe.git with the following command and navigate to the greenPipe directory before installation:

```
git clone https://github.com/snizam001/greenPipe.git
cd greenPipe
```

After downloading and navigating to the greenPipes folder, run `bash Install.sh` in the terminal. This script will automatically create a conda environment named as "greenpipes" and will install all dependencies in this environment. During the installation, it will ask to users to choose the genome of their interest from a list. After installation, user can activate this environment using command `conda activate greenpipes` and then run greenPipes. If the command `greenPipes --help` does not produce any errors, it indicates that greenPipes has been successfully installed.

2.2 Dependencies

Besides various packages and modules of R, PERL and PYTHON, greenPipes depend on following:

Fastqc https://www.bioinformatics.babraham.ac.uk/projects/fastqc/
fastq_screen https://www.bioinformatics.babraham.ac.uk/projects/fastq_screen/
bedtools https://bedtools.readthedocs.io/en/latest/
Trim galore https://www.bioinformatics.babraham.ac.uk/projects/trim_galore/
bowtie2 https://bowtie-bio.sourceforge.net/bowtie2/index.shtml
bwa https://bio-bwa.sourceforge.net/
nvBowtie https://github.com/NVlabs/nvbio
samtools http://www.htslib.org/
HOMER http://homer.ucsd.edu/homer/introduction/install.html
SEACR https://github.com/FredHutch/SEACR.
deepTools https://deeptools.readthedocs.io
subread https://subread.sourceforge.net/
sambamba https://lomereiter.github.io/sambamba/
seqtk https://github.com/lh3/seqtk
meme https://meme-suite.org/meme/

2.3 greenPipes: Datasets

2.3.1 Genome index files and black listed regions

To align reads with the reference genome, the pipeline requires genome index files. Pre-made Bowtie2 indexes (for paired-end reads) for the human and *Drosophila melanogaster* (spike-in) genomes can also be obtained from `https://osf.io/ruhj9`. If the user is working with a different organism, they can download the FASTA and GTF files for the genome of interest and generate Bowtie2 or BWA indexes. Follow the instructions provided at `https://bowtie-bio.sourceforge.net/bowtie2/manual.shtml#indexing-a-reference-genome` or `https://bio-bwa.sourceforge.net/bwa.shtml` for Bowtie2 and BWA, respectively. We recommend that the user visit `https://benlangmead.github.io/aws-indexes/bowtie` and `https://bowtie-bio.sourceforge.net/bowtie2/index.shtml`. These websites have bowtie2 reference genomes for many organisms. If the user wants to share index files of their reference genome of interest, we will be happy to upload it at `https://osf.io/ruhj9`. In this case the community working on the same model organism will benefit and the uploader will be acknowledged. Reference genome and its indexes must have `chr` syntax in the name of chromosomes.

Additionally, the indexed genome of *Escherichia coli* has been uploaded and can be utilized as a spike-in as suggested earlier (`https://doi.org/10.7554/eLife.46314`). However, it is recommended to initially evaluate the correlation of *E. coli* contamination with the actual externally added spike-in in the user's laboratory setup. In our own setup, we did not observe a correlation between the total number of *E. coli* reads and the total number of spike-in reads added externally (**Figure 2.2**). This discrepancy could be attributed to the low number of *E. coli* reads, leading to higher variations in our laboratory conditions.

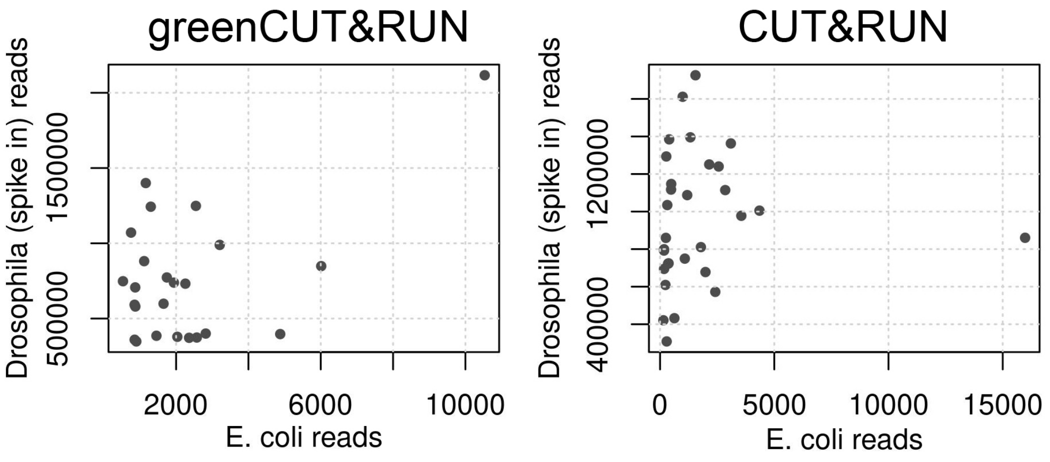

Figure 2.2: Correlation of the total number of *Drosophila melanogaster* (version BDGP5.75) reads with *E. coli* (*Escherichia coli* strain BL21, NCBI ID = CP010816.1) contaminations in greenCUT&RUN experiments. Each dots in figure represents single experiment. When the genome of *Escherichia coli* str. K-12 substr. MG1655 was employed for read alignment, we observed consistent results (data not shown).

The ENCODE blacklist comprises a thorough collection of regions in the human, mouse, worm, and fly genomes characterized by anomalous, unstructured, or elevated signals in next-generation sequencing experiments, irrespective of the cell line or experimental conditions.

Therefore, these regions should be excluded from the analysis. The blacklisted regions of the ENCODE for the human genome in BED format are provided in the *EncodeBlackListRegions* folder at https://osf.io/ruhj9. Users have the flexibility to generate their own list of blacklisted regions in a BED file.

2.3.2 Annotation dataset

The greenPipes database encompasses an extensive compilation of annotations stored in a BED file format. Within the database, user can find an annotation library developed by Kichaev, G et al (2017). This library was designed for the human genome version hg19 and is accessible for download at https://github.com/gkichaev/PAINTOR_V3.0/wiki/2b.-Overlapping-annotations[1]. For hg38, download data from folder Annotation available at https://osf.io/ruhj9. This dataset is generated by lifting over hg19 to hg38. Therefore, users may find less annotations in hg38 file compared to hg19. This dataset is summarized in **Table 2.1**.

Besides this, user can also use enhancer - gene interaction dataset for hg19 generated by Gao, T. et al (2019)[2]. This dataset contains enhancer - gene interaction identified in different cell lines and tissues: A375, A549, AML blast, Astrocyte, BJ, Bronchia epithelial, Caco2, CD14, CD19, CD20, CD34, CD36, CD4, CD8, Cerebellum, CUTLL1, DOHH2, ECC1, ESC neuron, Esophagus, Fetal heart, Fetal kidney, Fetal muscle leg, Fetal placenta, Fetal small intestine, Fetal spinal cord, Fetal stomach, Fetal thymus, FT246, FT33, GM10847, GM12878, GM12891, GM12892, GM18505, GM18526, GM18951, GM19099, GM19193, GM19238, GM19239, GM19240, H1, H9, HCC1954, HCT116, HEK293, HEK293T, HeLa, HeLaS3, HepG2, HFF, HL60, hMADS3, HMEC, hNCC, HSMM, HT1080, HT29, HUVEC, IMR90, Jurkat, K562, Calu3, Kasumi1, KB, Keratinocyte, Left ventricle, LHCNM2, Liver, LNCaPabl, LNCaP, Lung, MCF10A, MCF7, ME1, Melanocyte, melanoma, Mesendoderm, MS1, Myotube, Namalwa, NB4, NHDF, NHEK, NHLF, NKC, OCILy7, Osteoblast, Ovary, PANC1, Pancreas, Pancreatic islet, PBMC, PC3, PrEC, SGBS adipocyte, Skeletal muscle, SKNSH, SKNSH RA, Small intestine, Sperm, Spleen, T47D, T98G, th1, Thymus, U-2 OS, VCaP and ZR7530. Note that length of some of the enhancers in this databases is too high (> 2500 base pairs). For hg38, users can download data Enhancer-gene-interaction-hg38.tar.gz from folder Annotation available at https://osf.io/ruhj9. To generate this, all enhancers with size >2500 base pairs (bp) were removed. To download enhancer-gene interaction datasets for mouse and Drosophila, go to http://www.enhanceratlas.org/downloadv2.php. Users have the flexibility to generate their own files in a BED file, which can be used in the greenPipes pipeline for annotation.

In case that the users do not find enhancer-gene interaction dataset for cell line and tissue of their interest, follow the tutorial at chapter 16 section: Trans-elements/factors: Integration with transcriptomics.

2.3.3 Test dataset

The test dataset is available for download from the *Test data* directory, accessible at https://osf.io/ruhj9.

[1]Gleb Kichaev, Megan Roytman, Ruth Johnson, Eleazar Eskin, Sara Lindström, Peter Kraft, Bogdan Pasaniuc (2017). “Improved methods for multi-trait fine mapping of pleiotropic risk loci”. Bioinformatics, 33 (2), 248–255.

[2]Tianshun Gao and Jiang Qian (2019). “EnhancerAtlas 2.0: an updated resource with enhancer annotation in 586 tissue/cell types across nine species”. Nucleic Acids Res, 48 (D1), D58–D64.

Table 2.1: Comprehensive annotation library of Kichaev, G et al (2017) which can be used in UserAnnotation mode. Source of table is https://github.com/gkichaev/PAINTOR_V3.0/wiki/2b.-Overlapping-annotations. This dataset was developed to annotate single nucleotide polymorphism (SNPs) but it can also be used to annotate peaks

Collection	Description	Amount
FANTOM5	enhancer elements from fantom5 consortium	114 annotations
GeneElements Gencode	Gene Elements from GenCode	8 annotations
Hnisz Cell 2013 SuperEnhancer	Super Enhancers	88 annotations/cell types
Maurano Science 2012 DHS	DHS	352, some experimental replicates
RoadMap Assayed NarrowPeak	CHIP-seq peaks assayed in the RoadMap	1057 total annotations, 127 cell types, variable number of marks
Roadmap ChromeHMM 15 state	15 State CHROME-HMM model	1905 annotations, 15 states, 127 cell types
RoadMap Dyadic	Overlap of DHS with BivFlnk	111 annotations/cell types
RoadMap Enhancers	Overlap of DHS with EnhG, Enh, and EnhBiv for enhancers	111 annottions/cell types
RoadMap Imputed NarrowPeak	All functional marks imputed by Chrome-Impute	4061 annotations, 127 cell types, 32 functional marks
RoadMap Promoter	Overlap of DHS with TssA, TssAFlnk, and TssBiv	111 annotations/cell types
TFBS	Transcription factor binding sites	165 annotations
Thurman Nature 2012 DHS	DHS	54 annotations

Chapter 3

Modes and options overview, experiment-specific input files in greenPipes, quick start and help

3.1 Modes and options

The greenPipes pipeline encompasses several modes, including `qc`, `alignment`, `equalRead`, `qcExperiment`, `contamination`, `initPeakCalling`, `qcTagDirectories`, `callPeaks`, `idr`, `doughnut`, `PeakComparison`, `annotation`, `UserAnnotation`, `coverageTracks`, `cutfrequency`, `initHeatmap`, `heatmap`, and `piggyBack`. Each mode has its own set of associated options and parameters, which we will delve into in detail in the next chapters. Essentially, these options and arguments fall into three categories: (1) *Required arguments*, which are mandatory, (2) *Common arguments*, necessary for most modes, and (3) *Optional arguments*, specific to only a few modes.

Required arguments are mandatory and includes `--modes` and `--outputdir` options. More than one mode can be specified as comma-separated values e.g. `--modes qc,alignment`. These modes should be executed in a stepwise manner; for example, when performing alignment and peak calling, use the following command: `--modes alignment,initPeakCalling,callPeaks`. It is not permissible to instruct in the order of `--modes initPeakCalling,callPeaks,alignment`, because peaks cannot be called before aligning reads, and attempting to do so will result in an error. Users are not obligated to run all modes. They can skip certain modes based on their preferences. For instance, if the user does not wish to check contamination, utilize `qcExperiment`, or employ `doughnut mode`, these steps can be omitted. Another mandatory argument is `--outputdir`. Specify the full path of the folder, where you want to store all output files, tables, and figures. If the specified directory does not exist, the greenPipes pipeline will automatically create it. The pipeline autonomously generates folders in all modes.

Common arguments includes `--inputdir`, `--inputfile`, `--libraryType` and `--threads`. Specify the full path of the folder containing all fastq files in gz (gzip) format using `--inputdir` option. With `--inputfile` option, users can provide the path of the sample sheet. This sheet has the information of the experimental design. See section Sample sheet for experimental design for detail. The paired- and single-end libraries can be specified by argument *single* and *pair*, respectively using option `--libraryType`. Wherever necessary and applicable, greenPipes uses multi-threads. By default, it uses the total number of cpu or threads present in the computer minus 2. To decrease the compu-

tational burden, user can specify less number of threads using `--threads` command. To find the total number of cpu or threads in the computer (ubuntu) type `lscpu` in terminal. If user is using modes: `idr`, `doughnut`, `PeakComparison` or `UserAnnotation`, these *common arguments* are not required. But, if instruction is given to run other modes also like `--modes qc,alignment,initPeakCalling,callPeaks,PeakComparison`, then these arguments required.

3.2 Experiment specific files

3.2.1 Sample sheet for experimental design

The greenPipes pipeline requires a single input file or sample sheet containing information about the experimental design. This file should be a tab-separated text file with the following format: each line or row corresponds to a single experiment. The first two columns are reserved for controls, followed by the next two columns for the experimental conditions. The last column is designated for the experiment's name, which is chosen by the user. The experiment name should not contain spaces or special characters; users can use underscores ('_') instead of spaces. For paired-end reads, the left-hand reads (denoted as _1 or _R1) should occupy the first and third columns, while the right-hand reads should be in the second and fourth columns. An example input file named `SampleInfo.txt` is available at https://osf.io/ruhj9/ in the *Test data* folder. The option `--inputfile` is utilized to submit the `SampleInfo.txt` file. The input file should adhere to the format outlined in **Table 3.1** and is necessary for all modes except in `idr`, `doughnut`, `PeakComparison`, and `UserAnnotation`.

Table 3.1: Suppose you have a sample "S" undergoing two different conditions, 'm' and 'n.' In this case, you can denote experiments as Sm-exp and Sn-exp for conditions 'm' and 'n,' respectively. If there are controls, you can label controls as Sm-ctrl and Sn-ctrl for conditions 'm' and 'n,' respectively. Then format of the sample sheet for single and paired-end reads indicating independent experimental design will look like as given in table 3.1.

**Single end reads**				
Sm-ctrl.fastq.gz	<NA>	Sm-exp.fastq.gz	<NA>	Sm
Sn-ctrl.fastq.gz	<NA>	Sn-exp.fastq.gz	<NA>	Sn
**Paired-end reads**				
Sm-ctrl-R1.fastq.gz	Sm-ctrl-R2.fastq.gz	Sm-exp-R1.fastq.gz	Sm-exp-R2.fastq.gz	Sm
Sn-ctrl-R1.fastq.gz	Sn-ctrl-R2.fastq.gz	Sn-exp-R1.fastq.gz	Sn-exp-R2.fastq.gz	Sn

Users can play with this sample sheet for the analysis of a subset of samples. For instance, if a user has 10 samples, they can prepare the sheet for all samples similar to **Table 3.1** and perform quality control filtering and alignment. Later, the user may wish to explore cutfrequency around DNA motifs for only four samples. These four samples could represent a transcription factor, while the other 6 samples might be genome-wide histone modification profiles. In this case, the user can copy and paste the relevant four rows from the original sample sheet into a new text file. They can then use this new text file as input for `--inputfile` and run the `cutfrequency` mode. In this way, users can conduct the analysis only on a subset of samples.

3.2.2 Sheet for peak comparison among different experiments

To compare the peaks of individual experiments, the greenPipes pipelines needs an additional file. If user wants to compare sampleA to sampleB and sampleC, and sampleB with sampleC, then prepare a text file and specify it using `--compareInfile` option. The name of the samples should exactly match with last fifth column of sample sheet. Format of this text file should be as given in **Table 3.2**.

Table 3.2: Format of the sample sheet indicating design of comparison. This file will be used as input in –compareInfile option.

sampleA	sampleB
sampleA	sampleC
sampleB	sampleC

3.3 Quick start

After setting up greenPipes, users can initiate data analysis directly. First, they have to generate a sample sheet for experimental design (Samplesheet.txt) and a sheet for peak comparison (compare.txt), as discussed in the previous section. Assume the user wants to:

1. Align good-quality reads with the genome of interest and calculate spike-in reads (applicable modes = `qc` and `alignment`).
2. Select an equal number of aligned reads among experiments (mode = `equalRead`).
3. Obtain an initial overview of the datasets (mode = `qcExperiment`).
4. Call peaks (mode = `initPeakCalling` and `callPeaks`).
5. Find differential peaks (mode = `PeakComparison`).
6. Identify black and white peaks (mode = `PeakComparison`).
7. Distinguish bulky and non-bulky peaks (mode = `PeakComparison`).
8. Annotate peaks (mode = `annotation`).
9. Annotate peaks with the user's own list of annotations (mode = `UserAnnotation`).
10. Generate spike-in normalized coverage files for visualization in IGV (mode = `coverageTrack`).
11. Generate a heatmap on the list of peaks (mode = `heatmap`).
12. Integrate greenCUT&RUN or CUT&RUN peaks with proteomics data (mode = `piggyBack\verb`).
13. Generate cutfrequency around a DNA motif (mode = `cutfrequency`).

Let's assume that the experiment (greenCUT&RUN or CUT&RUN) is performed on human cells and includes *Drosophila* DNA as spike-in normalization controls. In this case, we require the bowtie2 reference genome, fasta file, and genome file of the human genome. Suppose that files are located at /Database/hg38/GenCode/GRCh38.p13, /Database/hg38/GenCode/GRCh38.p13.fa, and /Database/hg38/GRCh38.p13.genome, respectively. For the spike-in, we need the bowtie2 indexed reference genome of *Drosophila*. Suppose that file is located at /Database/Drosophila/Drosophila_melanogaster. The library is sequenced in paired-end mode and genome-wide profiling was performed for NF-YA subunit of the NF-Y complex, which binds to a CCAAT sequence. Its DNA motif is named on the JASPAR

website (http://jaspar.genereg.net/) as "MA0060." In the IP-MS (immunoprecipitation-based mass-spectrometry), the user have already found significant interactors of NF-YA. Protein IDs of these interacting proteins are stored in a file named piggyBack-lProtein.txt. In this case, the user wants to explore piggyback DNA binding events also. Besides this, the user wants to perform spike-in normalization at each possible step. Finally, suppose, BED file with location of the ENCODE blacklisted regions are present at /Database/hg38-blacklist.v2.bed; then, the user can use the following command to run the greenPipes pipeline:

```
greenPipes \
--modes qc,alignment,equalRead,qcExperiment,\
        initPeakCalling,callPeaks,PeakComparison,\
        annotation,UserAnnotation,coverageTrack,\
        heatmap,piggyBack
--inputdir $(pwd)/Fastq \
--inputfile ./Samplesheet.txt \
--compareInfile ./compare.txt \
--libraryType pair \
--outputdir $(pwd) \
--refgenome ~/Database/hg38/GenCode/GRCh38.p13 \
--spike-in ~/Database/Drosophila/Drosophila_melanogaster \
--blackListedRegions ~/Database/hg38-blacklist.v2.bed  \
--sFasta ~/Database/hg38/GenCode/GRCh38.p13.fa \
--annFiles $annDir/ATACseq.bed,\
            $annDir/H3K4me3.bed \
--annName   ATAC,H3K4me3 \
--genomeFile ~/Database/hg38/GRCh38.p13.genome \
--covSpike True
--covSpike_NormalizationFormula 2
--hCovComp coverage
--covExprType gCR
--cMotif True
--cMaN MA0060
--cGVersion hg38
--cCenter 8
--lProt piggyBack-lProtein.txt
--mPwm MA0060
--sProt CCAAT
--Species human
--gVer hg38
```

3.4 Getting help, error and log file

To seek assistance and understand the various options and arguments available in this pipeline, utilize the command `greenPipes --help`. Each option and argument will be succinctly explained. The pipeline encompasses different modes, including qc, contamination, alignment, equalRead, qcExperiment, initPeakCalling, qcTagDirectories, callPeaks, idr, doughnut, PeakComparison, annotation, UserAnnotation, coverageTracks, cutfrequency, initHeatmap, heatmap,

and piggyback. Each mode is equipped with its own set of options and arguments. When the `green --help` command is executed in the terminal, modes associated with options will be highlighted in green.

```
greenPipes --help

usage:

greenPipes pipeline (version 3.0): April, 2023

greenPipes [required arguments] [common arguments]
                                [optional arguments]

optional arguments:
-h, --help            show this help message and exit
--effectiveGenomeSize EFFECTIVEGENOMESIZE
                callPeaks, idr, coverageTracks, initHeatmap
                mode: effective genome size. human (2913022398:
                hg38, 2864785220: hg19), mouse (2652783500:
                GRCm38,
                2620345972: GRCm37) and fruitfly (142573017:dm6,
                162367812: dm3). (default is 2913022398 which is
                equivalent for the human genome)

.. cut ..


--dPeakfiles DPEAKFILES
                 doughnut mode: Path of the peak file for
                doughnut mode in bed format as comma separated
                values e.g. /home/xyz/exp1.Clean.bed,
                /home/xyz/exp2.Clean.bed.
--dNames DNAMES        doughnut mode: name of the experiment for
                each bed file given with --dPeakfiles e.g.
                exp1,exp2.

required arguments
_____________________:
--modes MODES   run mode. Multiple modes can be provided
                as comma-separated values e.g. --modes qc,
                alignment.
                Choices are: qc, contamination, alignment,
                equalRead, qcExperiment, initPeakCalling,
                qcTagDirectories, callPeaks, idr, doughnut,
                PeakComparison, annotation, UserAnnotation,
                coverageTracks, cutfrequency, initHeatmap,
                heatmap,
                piggyBack,
```

```
--outputdir OUTPUTDIR
                output directory (provide full path)

common arguments
_____________________:
--inputdir INPUTDIR   input directory having fastq files
                (provide full path)
--inputfile INPUTFILE
                        input file in .txt format
--libraryType single,pair
                        type of the library
--threads THREADS     number of threads (default is:
                        total CPU - 2)
```

See all options at the end of handbook.

The greenPipes pipeline generates a log file in the folder from where it is running (the current directory). Most errors, along with their causes and solutions, will be displayed on the terminal. If the information is not printed, refer to the log file to identify the cause of the error. While this document provides comprehensive information about the pipeline, if users have any further questions, they can contact us at `m.timmers@dkfz-heidelberg.de` or `n.sheikh@dkfz-heidelberg.de`. We will assist users. It is important to note that assisting with errors arising from dependencies may be challenging. In such cases, we recommend users to contact the developer of the dependencies. The likelihood of questions or problems increases when users employ greenPipes with genomes other than human.

Chapter 4

Quality control (qc)

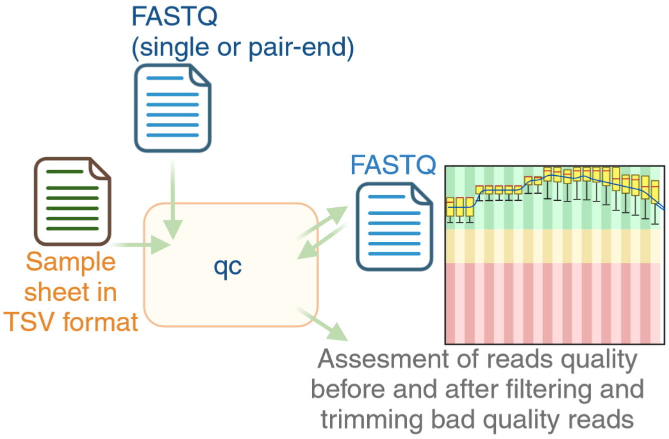

Figure 4.1: The workflow of the qc mode of the greenPipes.

This constitutes the initial stage of the pipeline and operates independently of other modes. The workflow of this mode is illustrated in **Figure 4.1**. During this process, it trims and eliminates low-quality nucleotides while discarding reads of poor quality. Subsequently, the pipeline generates several quality indices for both the initial and filtered FASTQ files. It employs the tool (1) trim_galore for trimming and filtering out poor-quality reads and (2) fastqc to assess the quality of the FASTQ files both before and after quality control filtering. The default parameters for both tools are applied. This mode does not entail any specific optional arguments. We suggest that users refer to `https://www.bioinformatics.babraham.ac.uk/projects/trim_galore/` and `https://www.bioinformatics.babraham.ac.uk/projects/fastqc/` for insights into the default parameters employed in the analysis and to gain a better understanding of the quality control indices. An illustrative command for paired-end reads is provided below:

```
greenPipea \
--inputdir $(pwd)/Fastq \
--inputfile ./Samplesheet.txt \
--libraryType pair \
--outputdir $(pwd) \
--modes qc
```

For single-end reads, users are required to specify `--libraryType single`. This mode will generate two distinct directories, namely *Trim_galore* and *FastqQC*. The former will contain

all trimmed and quality control-filtered reads, while the latter will store summary graphs, tables, and an HTML file from the FastQC tools.

```
output folder
|-- Trim_galore/
    |-- SampleA_control_R1.fastq.gz_trimming_report.txt
    |-- SampleA_control_R1_val_1.fq.gz
    |-- SampleA_control_R2.fastq.gz_trimming_report.txt
    |-- SampleA_control_R2_val_2.fq.gz
.. cut ..

|-- FastqQC
    |-- SampleA_control_R1_fastqc.html
    |-- SampleA_control_R1_fastqc.zip
    |-- SampleA_control_R1_val_1_fastqc.html
    |-- SampleA_control_R1_val_1_fastqc.zip
    |-- SampleA_control_R2_fastqc.html
    |-- SampleA_control_R2_fastqc.zip
.. cut ..
```

Chapter 5

Alignment and selection of equal reads

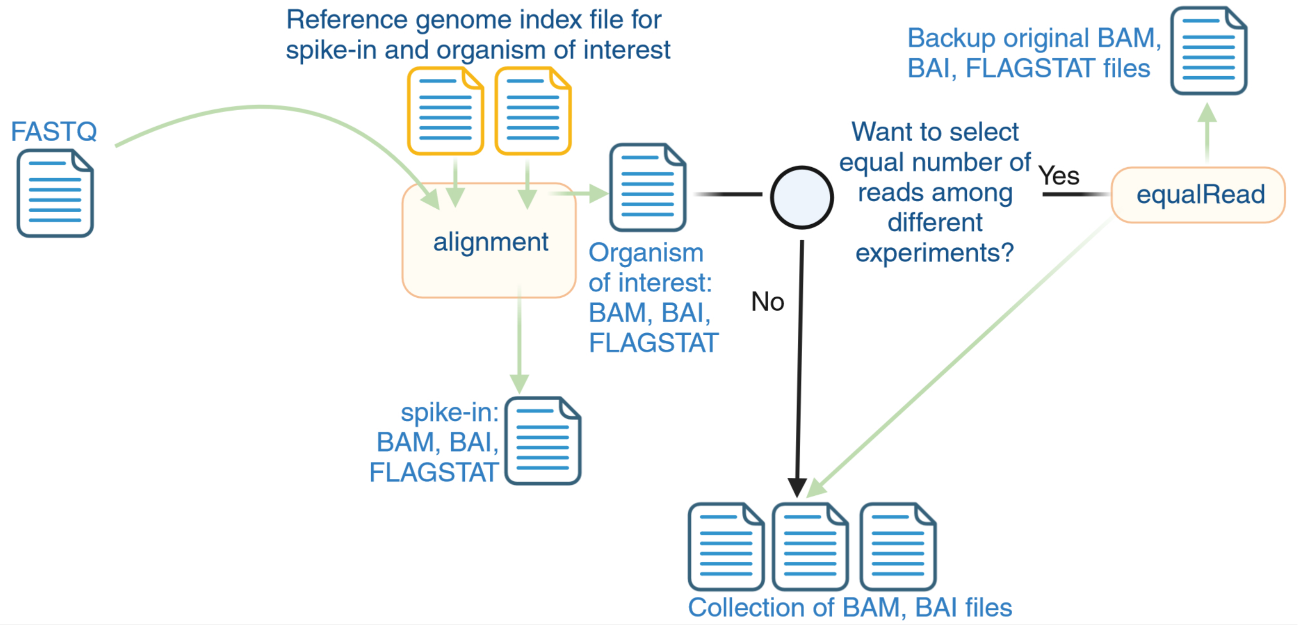

Figure 5.1: The workflow of the alignment and equalRead mode of the greenPipes.

5.1 alignment

After the quality control filtering, the reads undergo alignment to the genome of interest and the genome of the spike-in control. For paired-end and single-end reads, the greenPipes pipeline employs bowtie2 (`https://bowtie-bio.sourceforge.net/bowtie2/index.shtml`) and bwa (`https://github.com/lh3/bwa`), respectively. This mode relies on the output file from the quality control or `qc` mode. The workflow of this mode is illustrated in the **Figure 5.1**. Optional arguments associated with this mode include `--refgenome`, `--spike-in`, `--alignParam`, and `--gpu (True/False)`. Users can specify the bowtie2 or bwa index of the genome for the organism of interest and the spike-in reference genome through `--refgenome` and `--spike-in`, respectively. The default bowtie2 parameters in this pipeline are: `--dovetail --local --very-sensitive-local --no-unal --no-mixed --no-discordant -I 10 -X 700` (based on Meers et al. (2019))[1]. For single-end reads,

[1]Meers et al. (2019). "Improved CUT&RUN chromatin profiling tools" eLife 8:e46314

the default parameters of the bwa program were used. If users wish to modify alignment parameters, they can input the desired parameters as a series of comma-separated values enclosed in square brackets using the `--alignParam` option. For example, for bowtie2: `[--no-unal, --no-mixed, --no-discordant, -I, 0, -X, 1500]`. The greenPipes pipeline employs the BWA-MEM algorithm for alignment. Users are advised to modify only the parameters applicable to the bwa mem algorithm. By utilizing `--gpu` option, users can leverage the graphical processing unit (GPU) of their computer to expedite the alignment process. Faster alignment can be achieved by following the instructions provided at https://nvlabs.github.io/nvbio/nvbowtie_page.html. In this context, `--gpu` utilizes nvBowtie, and therefore genome indexes need to be compatible with nvBowtie. If `--gpu` is set to True and users wish to incorporate additional arguments specific to nvBowtie, they can use the same `--alignParam` option. Refer to the extra options of nvBowtie at https://nvlabs.github.io/nvbio/nvbowtie_page.html.

Some laboratories focus on ribosomal RNAs, which pose a challenge due to their repetitive nature and the absence of genomic locations in the current human genome version (hg38). To address this, collaborative efforts, such as the telomere2telomere project (https://www.genome.gov/about-genomics/telomere-to-telomere), have emerged. On March 31st, 2023, the group released the first gapless human genome sequence, including the sequences and genomic locations of ribosomal RNAs. We have made the telomere2telomere genome index available on https://osf.io/ruhj9/ in the "Reference genomes" folder, which users can utilize for aligning paired-end reads.

An example command for paired-end reads is as follows:

```
greenPipes \
--mode alignment\
--inputdir $(pwd)/Fastq \
--inputfile  ./SampleInfo.txt \
--libraryType pair \
--outputdir $(pwd) \
--refgenome ~/Database/hg38/GenCode/GRCh38.p13 \
--spike-in ~/Database/Drosophila/Drosophila_melanogaster \
--blackListedRegions ~/Database/hg38-blacklist.v2.bed
```

The pipeline offers flexibility to execute multiple modes in a single command. Users can utilize the `--mode` option and provide several modes as comma-separated values. For instance, if the user wish to run both the quality control (`qc`) and `alignment` modes simultaneously, an example command for paired-end reads would appear as follows:

```
greenPipes \
--mode qc,alignment \
--inputdir $(pwd)/Fastq \
--inputfile  ./SampleInfo.txt \
--libraryType pair \
--outputdir $(pwd) \
--refgenome ~/Database/hg38/GenCode/GRCh38.p13 \
--spike-in ~/Database/Drosophila/Drosophila_melanogaster \
--blackListedRegions ~/Database/hg38-blacklist.v2.bed
```

An example command for `qc` and `alignment` of single-end reads will look like this:

```
greenPipes \
--mode qc,alignment \
--inputdir $(pwd)/Fastq \
--inputfile  ./SampleInfo.txt \
--libraryType single \
--outputdir $(pwd) \
--refgenome ~/Database/hg38/GenCode/GRCh38.p13 \
--spike-in ~/Database/Drosophila/Drosophila_melanogaster \
--blackListedRegions ~/Database/hg38-blacklist.v2.bed
```

In this mode, two separate directories, namely *Bamfiles* and *spike-in*, will be generated. The alignment files for the genome of interest and spike-in will be stored in *Bamfiles* and *spike-in*, respectively. For each sample, three files will be generated: (1) an alignment file (bam file), (2) an alignment index file (bai file), and (3) an alignment summary (flagstat file).

```
output folder
|-- Bamfiles
    |-- SampleA_control.bam
    |-- SampleA_control.bam.bai
    |-- SampleA_control.Flagstats.txt

.. cut ..

|-- spike-in
    |-- SampleA_control.bam
    |-- SampleA_control.bam.bai
    |-- SampleA_control.Flagstats.txt

.. cut ..
```

5.2 equalRead

In theory, the deeper sequencing of libraries allows for the capture of highly dynamic and rare binding events. For instance, when comparing two experiments—one sequenced with 6 million reads and the other with 20 million reads—you might prefer to proceed with an equal number of reads across samples to ensure that observed differences are not solely due to technical variations. However, it is worth noting that this mode is applicable only when additional reads are not planned for the lower-depth samples.

This mode utilizes sambamba (https://lomereiter.github.io/sambamba/) for subsampling and relies on the output file of the alignment mode. This implies that the user should first run qc and alignment; only then can the user execute equalRead. Otherwise, the pipeline will report an error.

This mode comes with optional arguments, including `--SelectReads` and `--reverseName\_equalRead (True/False)`. By `--SelectReads`, users can specify the number of reads to be selected in each sample. It is essential to note that this number

represents an absolute count of paired-end reads (e.g., 5,000,000), not a proportion or frequency. If no number is specified, the pipeline will automatically identify the experiment with the minimum number of reads among all samples. Subsequently, it will subsample reads to ensure that all samples have the same number of reads as the identified minimum. The pipeline calculates the proportion of reads decreased in each sample and then subsamples reads in the control and all spike-in bamfiles accordingly. Additionally, if a set of samples has a minimum read number (N_{min}) in a sample X, users cannot specify an equal number of reads greater than N_{min}; the pipeline will report an error. This limitation is both biologically and technically sound, as users should avoid increasing the number of reads beyond the minimum, akin to amplifying an artifact like PCR duplicates. During this step, new files (*bam, *bai, and *Flagstats.txt) will be generated in the *Bamfiles* and *spike-in* folders, while the old files will be renamed (with an added *original* extension).

If a user has already selected equal reads in all samples and attempts to repeat the process, the program will report an error, reminding the user that an equal number of reads has already been selected. If the user genuinely wants to choose an equal number of reads again, they should use the `--reverseName\_equalRead` option first. This option removes the subsampled samples and restores the original files (*bam, *bai, and *Flagstats.txt). Afterwards, the user can subsample reads using `--SelectReads`. The pipeline is designed in this way to prevent loss of the original bam files and avoid running the alignment mode again and again.

An example command for selecting equal number of reads among all samples is as follows:

```
greenPipes \
--mode equalRead \
--inputdir $(pwd)/Fastq \
--inputfile  ./SampleInfo.txt \
--libraryType pair \
--outputdir $(pwd)
```

An example command for selecting 5,000,000 reads among all samples is as follows:

```
greenPipse \
--mode equalRead \
--inputdir $(pwd)/Fastq \
--inputfile  ./SampleInfo.txt \
--libraryType pair \
--outputdir $(pwd) \
--SelectReads 5000000
```

If user have already selected 5,000,000 reads, but now want to select 6,000,000 reads, then they can proceed as follows:

```
greenPipes \
--mode equalRead \
--inputdir $(pwd)/Fastq \
--inputfile  ./SampleInfo.txt \
--libraryType pair \
--outputdir $(pwd) \
--reverseName_equalRead True
```

```
greenPipes \
--mode equalRead \
--inputdir $(pwd)/Fastq \
--inputfile  ./SampleInfo.txt \
--libraryType pair \
--outputdir $(pwd) \
--SelectReads 6000000
```

New files will be generated in *Bamfiles* and *spike-in* folder. *original* are old files, while other files are new with equal number of reads.

```
output folder
|-- Bamfiles
    |-- SampleA_control.bam
    |-- SampleA_control.bam.bai
    |-- SampleA_control.Flagstats.txt
    |-- SampleA_controloriginal.bam
    |-- SampleA_controloriginal.bam.bai
    |-- SampleA_controloriginal.Flagstats.txt

.. cut ..

|-- spike-in
    |-- SampleA_control.bam
    |-- SampleA_control.bam.bai
    |-- SampleA_control.Flagstats.txt
    |-- SampleA_controloriginal.bam
    |-- SampleA_controloriginal.bam.bai
    |-- SampleA_controloriginal.Flagstats.txt

.. cut ..
```

Chapter 6

Quality control of the experiment

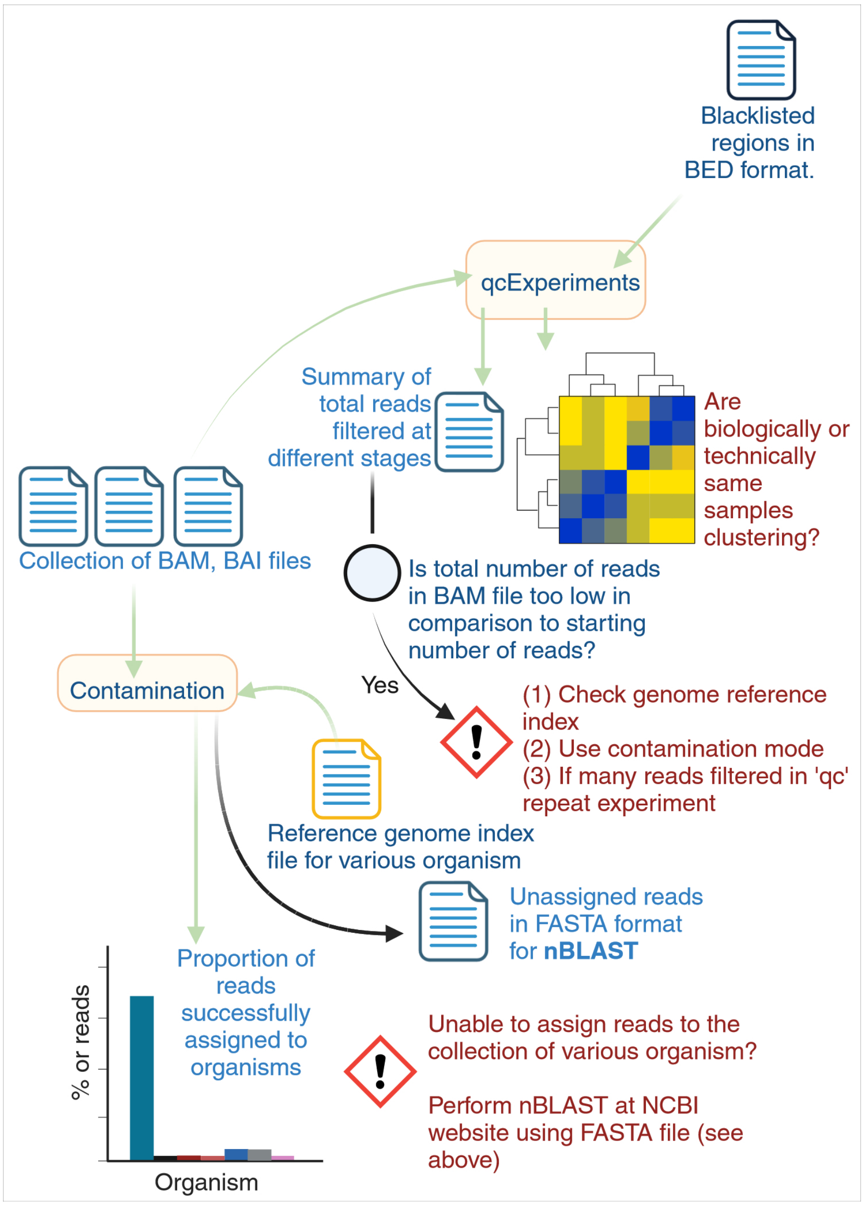

Figure 6.1: The workflow of the qcExperiments and contamination mode of the greenPipes.

6.1 qcExperiment

After quality control filtering and alignment, users can assess the quality of the experiments. This mode relies on the output file of the alignment mode.

Optional arguments associated with this mode include `--blackListedRegions`. In this parameter, users need to provide blacklisted regions in BED format relevant to their genome of interest. If the genome of interest is human and the version is hg38, users can utilize the hg38-blacklist-v2.bed file, which is available for download at https://osf.io/ruhj9/. If a similar file is not available for the genome of interest, users can either generate a BED file for the blacklisted regions or provide a dummy file to the pipeline. To create a dummy file, use the following command:

```
echo "chr\t0\t1" > dummyBlackListedregions.bed
```

An example command for paired-end reads to execute this mode is:

```
greenPipes \
--inputdir $(pwd)/Fastq \
--inputfile ./Samplesheet.txt \
--libraryType pair \
--outputdir $(pwd) \
--modes qcExperiment \
--blackListedRegions ~/Database/hg38-blacklist.v2.bed
```

A single directory *Bamfiles_QC* will be generated in this mode. In total, five files will be generated.

```
output folder
|-- Bamfiles\_QC/
    |-- Correlation.gz
    |-- Correlation.jpeg
    |-- Correlation-mat.tab
    |-- CorrelationRawCounts.txt
    |-- ReadCounts.txt
```

The ReadCounts.txt file provides a summary of the total number of reads that passed each step (refer to the example in **Table 6.1**). Users can review the count of reads at different stages, including the initial number from the FASTQ files, the quantity after quality control filtering, and the number of successfully aligned reads to both the genome of interest and the spike-in genome.

This file is very helpful for fine-tuning the design of experiment and analysis. In our lab, we observe ≈5% spike-in reads in both the control and experimental greenCUT&RUN setups. Conversely, in the CUT&RUN setup, we sometimes observe ≈10% and ≈15% spike-in reads for the experiment and control, respectively. If the number of spike-ins is either very high or very low, users can adjust their concentration in the experiment. If users notice a very low number of reads in the aligned BAM files, they should check whether the correct index file for the reference genome of interest was used. If the correct index file was used, the next step is to use the *contamination* mode to check for contamination in the samples. It is possible that the pool might

contain a higher number of mycoplasma reads. Besides this, *Pseudomonas putida* is often found in contaminated water and can be source of contamination. It is recommended for users to verify the count of reads that have successfully passed through the quality control mode. If the read count is excessively low, it is advisable to regenerate the raw data.

The ReadCounts.txt file has eleven columns. The first column contains the name of experiment, matching the fifth column of the sample sheet. The second and third columns show the total number of reads in the original/starting FASTQ files for the experiment and control, respectively. The fourth and fifth columns display the number of reads in FASTQ files after quality control filtering for the experiment and control, respectively. The sixth and eighth columns present the number of aligned reads to the reference genome of interest for the experiment and control, while the seventh and ninth columns show aligned reads to spike-in. The last two columns can be ignored.

Table 6.1: An example ReadCounts.txt file is provided. In the table, "FQ" and "Align" are abbreviations for FASTQ and BAM files, respectively, while "Expr" and "Ctrl" are abbreviations for Experiment and Control.

Name	Original Expr (FQ)	Original Ctrl (FQ)	Filter Expr (FQ)	Filter Ctrl (FQ)	Org Expr (Align)	Spike Expr (Align)	OrgCtrl (Align)	Spike Ctrl (Align)	For Expr	For Ctrl
SampleA	1045658.0	743696.0	1045570.0	743615.0	991693	54134	689140	58418	-	-
SampleB	1039702.0	660940.0	1039627.0	660613.0	991277	59240	618160	56823	-	-

The other Correlation* files are generated by the *multiBamSummary* function of deeptools (`deeptools.readthedocs.io/`). In these files, you can find the correlations among the experiments. The program uses Pearson correlation coefficients and removes outliers during the preparation of the figure. This step is especially helpful when using replicates, as replicates should exhibit a high correlation and cluster together in hierarchical clustering, as shown in **Figure 6.2 A**. If you are working with different mutants of the same protein, various CRISPR knockouts of the same protein, or different proteins altogether, you can check whether the effects are similar at the genomic level. If the effects are similar, the samples will cluster together, as illustrated in **Figure 6.2 B**. This step serves as a good starting point for the analysis of your dataset/s.

6.2 contamination

If a significant decrease in the number of reads in the alignment files (BAM files) compared to the initial FASTQ files is observed during the qcExperiment mode, It is essential to investigate the possibility of sample contamination. While other factors, such as using an incorrectly indexed genome, could contribute to the reduced read count, It is crucial to consider the potential for contamination. In some pipelines, PCR duplicates are typically removed from the dataset, leading users to suspect that a lower number of reads could be attributed to PCR duplicates. However, It is important to highlight that MNase, in certain instances, can generate very small fragments, causing reads from different cells to exhibit characteristics resembling PCR duplicates. As a result, the pipeline retains reads that might resemble PCR duplicates throughout its various stages. Therefore, the reduced read count in BAM files is not a consequence of PCR duplicate removal. Nevertheless, when users initiated their experiment with a limited quantity of DNA and utilized a high number of PCR cycles, it is advisable to assess the proportion of PCR duplicates using the picard tool (available at `https://broadinstitute.github.io/picard/`).

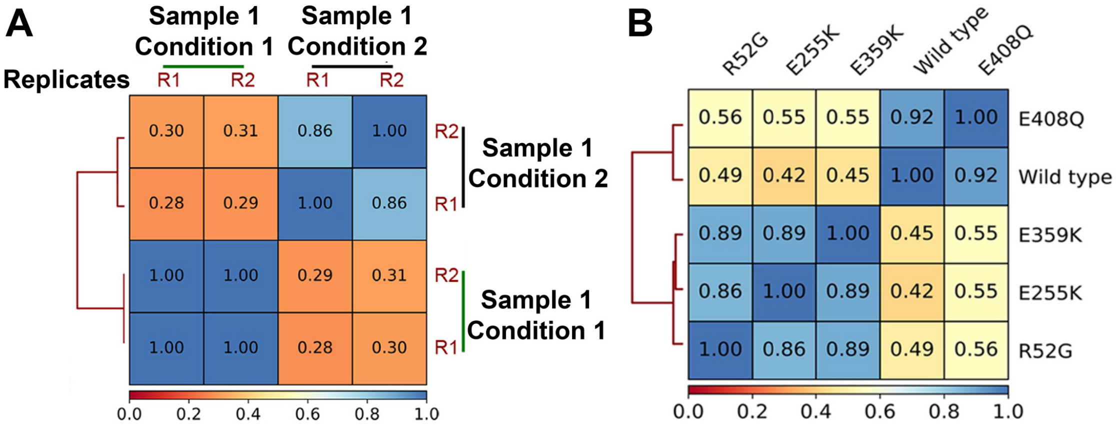

Figure 6.2: This image is produced in the qcExperiment mode. Panel A demonstrates the reproducibility of biological replicates, as determined by Pearson correlation coefficients. In Panel B, you can observe the similarities and differences in the genome-wide profiles of different mutants and the WildType of the same protein (MEN1 or Menin). Broadly, the R52G, E255K, and E359K mutations affect the genome-wide binding profile of MEN1 on chromatin, while there is little to no effect of the E408Q mutation in MEN1 in comparison to the WildType, as evident in the hierarchical clustering. Notice that the correlation coefficient among mutants E259K, E255K, and R52G is only 0.89, suggesting that the effect of distinct mutations might be different. To explore differences among mutants in detail, user can proceed to call peaks and identify differential peaks using the next modes (initPeakCalling, callPeaks, and PeakComparison) of greenPipes. Source of panel A is Nizamuddin et al (2021)[1] and B is Dreijerink et al (2022)[2]. Panel A is modified from its original source.

This mode relies on the output files from the alignment mode and utilizes the *fastq_screen* tool (https://www.bioinformatics.babraham.ac.uk/projects/fastq_screen/). Essentially, *fastq_screen* randomly selects 100,000 reads from each FASTQ file and aligns them with various genomes, which were downloaded and built during the installation of the *fastq_screen* tool (as illustrated in the **Figure 2.1**). This mode does not have any optional arguments. An example command for paired-end reads is provided below:

```
greenPipes \
--inputdir $(pwd)/Fastq \
--inputfile ./Samplesheet.txt \
--libraryType pair \
--outputdir $(pwd) \
--modes contamination
```

A single directory *Contamination* and sub-directories for each samples will be generated in this mode.

[1]Sheikh Nizamuddin, Stefanie Koidl, Tanja Bhuiyan, Tamara V Werner, Martin L Biniossek, Alexandre MJJ Bonvin, Silke Lassmann, HT Marc Timmers (2021). "Integrating quantitative proteomics with accurate genome profiling of transcription factors by greenCUT&RUN." Nucleic acids research 49(9): e49-e49.

[2]Koen Dreijerink, Ezgi Ozyerli-Goknar, Stefanie Koidl, Ewoud J van der Lelij, Priscilla van den Heuvel, Jeffrey J Kooijman, Martin L Biniossek, Kees W Rodenburg, Sheikh Nizamuddin, HT Marc Timmers. "Multi omics analyses of MEN1 missense mutations identify disruption of menin-MLL and menin JunD interactions as critical requirements for molecular pathogenicity". Epigenetics & Chromatin 15, 29 (2022).

```
output folder
|-- Contamination/
    |-- SampleA
        |-- SampleA_control\_R1_screen.html
        |-- SampleA_control\_R1_screen.txt
        |-- SampleA_control\_R1.tagged.fastq.gz
        |-- SampleA_control\_R1.tagged\_filter.fa
        |-- SampleA_control\_R1.tagged\_filter.fastq.gz
.. cut ..

    |-- SampleB
        |-- SampleB_control\_R1_screen.html
        |-- SampleB_control\_R1_screen.txt
        |-- SampleB_control\_R1.tagged.fastq.gz
        |-- SampleB_control\_R1.tagged\_filter.fa
        |-- SampleB_control\_R1.tagged\_filter.fastq.gz

.. cut ..
```

In the *html file, users can examine the reads aligning with different organisms. If the organism of interest is human, but there are numerous mouse or rabbit reads, users should check whether a mouse or rabbit antibody is being used. It is possible that a significant number of mouse or rabbit reads originate from the antibody solution, indicating the need for antibody purification. This scenario is specific to CUT&RUN or CUT&Tag experiments employing mouse or rabbit antibodies.

When nothing seems suspicious in the *html file, and users do still suspect contamination, they can inspect the *txt file generated for each sample in this mode. The last line of this file contains *%Hit_no_genomes*, revealing the percentage of reads not aligning with the genomes downloaded by *fastq_screen*. A high percentage suggests potential DNA contamination. In such cases, use the *filter.fa file generated by the pipeline and upload it to the NCBI Nucleotide BLAST tool (https://blast.ncbi.nlm.nih.gov/Blast.cgi). This file includes reads that did not align with genomes of *fastq_screen*. When selecting the program on the BLAST website, choose *Somewhat similar sequences (blastn)* and run the analysis. In the subsequent BLAST webpage, users will identify reads aligning with other organisms, helping to identify the source of the DNA contamination.

Chapter 7

Peak calling

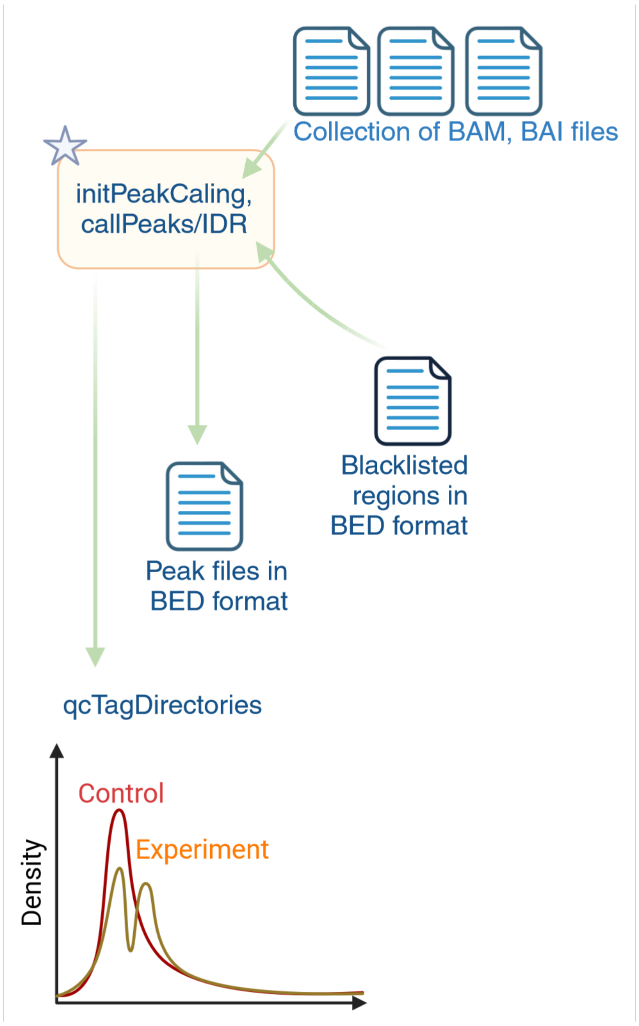

Figure 7.1: A simple workflow diagram illustrating initPeakCalling, qcTagDirectories, callPeaks and idr mode of the greenPipes.

One of the primary objectives in genome-wide profiling method like greenCUT&RUN is to determine the genomic locations of peaks. Various tools, such as HOMER (`http://homer.ucsd.edu/homer/`), MACS2 (`https://pypi.org/project/MACS2/`), and SEACR (`https://github.com/FredHutch/SEACR`), are available for peak calling. SEACR is specifically designed for CUT&RUN and CUT&Tag experiments. However, in our laboratory

setup, we observed suboptimal performance by SEACR in comparison to MACS2 and HOMER[1].

We noted that SEACR called either a lower number of peaks compared to other algorithms or identified a smaller proportion of peaks with the expected DNA motif in comparison to other common peak callers (**Table 7.1**). The identification of a higher number of peaks without CCAAT DNA motifs raises concerns about potential false-positive peaks by SEACR. Additionally, MACS2 exhibited fewer identified peaks, particularly in techniques other than greenCUT&RUN, and a lower percentage of peaks with CCAAT DNA motifs compared to HOMER. Overall, these observations suggest that HOMER is the preferred tool for peak calling, with MACS2 as the second-best option. This is the primary rationale behind the incorporation of HOMER support in the pipeline. Besides this, the use of MACS2 or MACS3 as a peak caller does not allow spike-in normalization as easy as HOMER. Some of the functions required for spike-in normalization like '–ratio' function of 'callpeak' mode are not supported in the newer versions of MACS (MACS2 or MACS3). I hypothesize that the challenges encountered by SEACR in our laboratory may be attributed to a lower and non-uniform background in the control samples. However, this is a speculative notion without solid analysis to substantiate it.

Table 7.1: Performance evaluation of peak calling tools in a genome-wide profiling experiment of NFYA. Source of this table is https://doi.org/10.1093/nar/gkab038. See supplementary table 2)

Technique	Total peaks (HOMER)	Peaks with Motifs (HOMER)	Total peaks (MACS2)	Peaks with Motifs (MACS2)	Total peaks (SEACR)	Peaks with Motifs (SEACR)
greenCUT &RUN	7433	6453 (86.82%)	11482	8263 (71.96%)	5253	3651 (68.2%)
CUT&RUN	5654	4953 (87.60%)	5326	4703 (88.30%)	43062	11380 (26.43%)
CUT&RUN (GFP)	3186	2721 (85.40%)	2860	2258 (78.95%)	1620	1092 (67.41%)

7.1 initPeakCalling

This mode depends on the output of the alignment mode. If HOMER is chosen, this step needs to be executed before peak calling. During this step, the pipeline generates tagDirectories for all samples, including controls and experiments. To understand the format of this directory, reference the documentation available here: `http://homer.ucsd.edu/homer/ngs/tagDir.html`.

The tagDirectory includes a file named *tagInfo.txt*, containing information about the total number of reads. The total number of reads can be adjusted using the `--totalReads` option in HOMER to meet specific requirements. The pipeline utilizes this option to generate spike-in-normalized total numbers of human reads for the control sample, ensuring that both the control and its associated experiment have the same level of spike-in. The total number of reads in the tagInfo file is employed to divide reads calculated in a bin. It is important to note that when the

[1] Sheikh Nizamuddin, Stefanie Koidl, Tanja Bhuiyan, Tamara V Werner, Martin L Biniossek, Alexandre MJJ Bonvin, Silke Lassmann, HT Marc Timmers (2021). "Integrating quantitative proteomics with accurate genome profiling of transcription factors by greenCUT&RUN." Nucleic acids research 49(9): e49-e49.

control sample has a higher number of spike-in compared to the experiment, the total number of reads in the tagInfo file will be increased at the same level as the spike-in.

Initially, when sequencing both the control and experiment at equal sequencing depth, the total number of normalized reads in the control for normalization can be calculated as follows. Assume that the total number of human (or of the organism of interest) and spike-in reads in experiment are E_h and E_S while in control are C_h and C_S, total spike-in normalized reads for experiment (E_T) and control (C_T) can be calculated as given in equation 1.

$$C_T = \frac{C_h}{E_S} \times C_S$$

There is no need to normalize (E_T) after normalizing control and thus value will be:

$$E_T = E_h$$

...**equation 1**

Equation 1 is same as given in greenCUT&RUN article[2]. Currently, we are sequencing controls at the 1/3 or 1/4 depth of experiments [Note that we never sequenced the control ≤4 million reads for control]. This practice does not compromise the quality of the experimental results and is also cost-effective. Here, mathematical formula for normalization was changed which is as follows:

$$C_T = C_h \times \left(\frac{C_S}{C_S + C_h}\right) \times \left(\frac{E_S + E_h}{E_S}\right)$$

There is no need to normalize (E_T) after normalizing control and thus the value will be:

$$E_T = E_h$$

...**equation 2**

Equation 2 is equivalent to Equation 1 when the experiment and control have equal reads; therefore, only Equation 2 is implemented in the pipeline. Equation 2 can be written as follows:

$$C_T = C_h \times \left(\frac{C_S}{E_S}\right) \times \left(\frac{E_S + E_h}{C_S + C_h}\right)$$

if,

$$C_S + C_h = E_S + E_h$$

then,

$$C_T = C_h \times \left(\frac{C_S}{E_S}\right) \times 1$$

or,

$$C_T = \frac{C_h}{E_S} \times C_S$$

which is equivalent to equation 1.

[2] Sheikh Nizamuddin, Stefanie Koidl, Tanja Bhuiyan, Tamara V Werner, Martin L Biniossek, Alexandre MJJ Bonvin, Silke Lassmann, HT Marc Timmers (2021). "Integrating quantitative proteomics with accurate genome profiling of transcription factors by greenCUT&RUN." Nucleic acids research 49(9): e49-e49.

Pipeline will generate a *Homer_normalization.txt* file in folder *Peaks* (an example is given in **Table 7.2**). User can track the read numbers in this file. This file has ten columns. The first column is the name of experiment. It matches with fifth column of sample sheet. The second and third column contains total number of reads in aligned bam files for experiment (E_h) and control (C_h), respectively. The fourth and fifth column contains the number of spike-in reads in experiment (E_S) and control (C_S), respectively. Ignore the sixth and seventh column. the eight and ninth column is equal to $\left(\frac{C_S}{C_S+C_h}\right)$ and $\left(\frac{E_S}{E_S+E_h}\right)$, respectively. The last column is equal to the spike-in normalized total control reads C_T.

Table 7.2: Example of Homer_normalization.txt

Experiment	Read number expr	Read number control	Read number spike-in expr	Read number spike-in control	spike-in-ratio	spike-in normalized controlRead-sOld	perReadsSpike expr	perReadsSpike ctrl	spike-in normalized controlRead-sNew
SampleA	991693	689140	54134	58418	0.93	743676.44	0.052	0.078	1040396.74
SampleB	991277	618160	59240	56823	1.04	592938.99	0.056	0.084	922826.93

We usually observe that $\left(\frac{C_S}{C_S+C_h}\right) > \left(\frac{E_S}{E_S+E_h}\right)$, which means controls always have higher amount of spike-in compared to experiments. If this condition is not satisfied in given dataset/s, pipeline will report an error like this: `spike-in reads per true-reads are less in control compared to your experiment. Consider peak calling without control (--Control\_homer False) or inititate peakcalling (init PeakCalling) with --spikeNormPeak False`. This condition is commonly observed when an experiment fails, although it is not a universal rule. The definition of 'failed' in this context is, when no peaks or less peaks are observed in the callPeaks mode. Experiment failure can occur for various reasons, such as a lack of sensitivity to capture binding events in CUT&RUN, antibody epitopes are buried inside a complex, or in both CUT&RUN and greenCUT&RUN, the protein of interest is highly dynamic or distant from DNA. Other factors may also contribute to experiment failure. In such cases, users have the option to disable spike-in normalization or call peaks without a control. However, It is important to be cautious, as this may lead to the identification of false-positive peaks in the samples.

Only one optional argument `--spikeNormPeak (True,False)` is associated with this mode. If you do not want to use spike-in normalization in peak calling, turn this off by specifying `--spikeNormPeak False`. By default, spike-in normalization is True. An example command for paired-end reads is as follows:

```
greenPipes \
--inputdir $(pwd)/Fastq \
--inputfile ./Samplesheet.txt \
--libraryType pair \
--outputdir $(pwd) \
--modes initpeakcalling
```

A single directory Tagdirectories and sub-directories for each samples will be generated in this mode.

```
output folder
|-- Tagdirectories/
    |-- SampleA_control
        |-- chr1.tags.tsv
        |-- chr2.tags.tsv

        .. cut ..

        |-- tagAutocorrelation.txt
        |-- tagCountDistribution.txt
        |-- tagInfo.txt

.. cut ..
```

7.2 qcTagDirectories

This mode depend on the output of initPeakCalling. It generate files and figures to assess the quality of tag directories. Please go through http://homer.ucsd.edu/homer/ngs/tagDir.html to understand the format of this folder.

An example command for paired-end reads is as follows:

```
greenPipes \
--inputdir $(pwd)/Fastq \
--inputfile ./Samplesheet.txt \
--libraryType pair \
--outputdir $(pwd) \
--modes qcTagDirectories
```

A single directory Tagdirectories_qualities will be generated in this mode.

```
output folder
|-- Tagdirectories_qualities/
    |-- SampleA_expr.jpeg
    |-- SampleA_expr.gCov.bed
    |-- SampleA_control.gCov.bed
    |-- SampleA_control.gCov-normalized.bed

.. cut ..
```

All *bed are intermediate files. The *sorted.bed file contains the genomic locations of all reads, *sorted.fragments.bed contains the genomic locations of fragments, *gCov.bed provides genomic coverage information, and *.gCov-normalized.bed contains details of spike-in-normalized coverage.

To gain basic information about tagDirectories, It is recommended to examine the *jpeg file. An illustrative example is presented in **Figure 7.2**. The upper two panels depict the number of tags per unique genomic position. In the alignment, we configured parameters to ensure that a

single fragment aligns only to the best genomic position. If duplicate fragments exist in the data, a higher *frequency of tags per position* will be observed for *Tag position* > 1.

It was predicted that greenCUT&RUN and CUT&RUN might produce a higher number of PCR duplicates like reads originating from different cells due to their smaller fragment size. However, in this sample, duplicates are observed to be minimal. If you notice a higher number of duplicates in your dataset, our recommendation is to repeat the same experiment using Unique Molecular Identifiers (UMIs) in library preparation. Check for the presence of PCR duplicates, and if none are observed, duplicates appearing in the upper panel are likely from different cells and they should not be removed from the data. Both middle panel shows the relative distance of forward and reverse reads. The lower panel shows the distribution of fragment length in experiment and control.

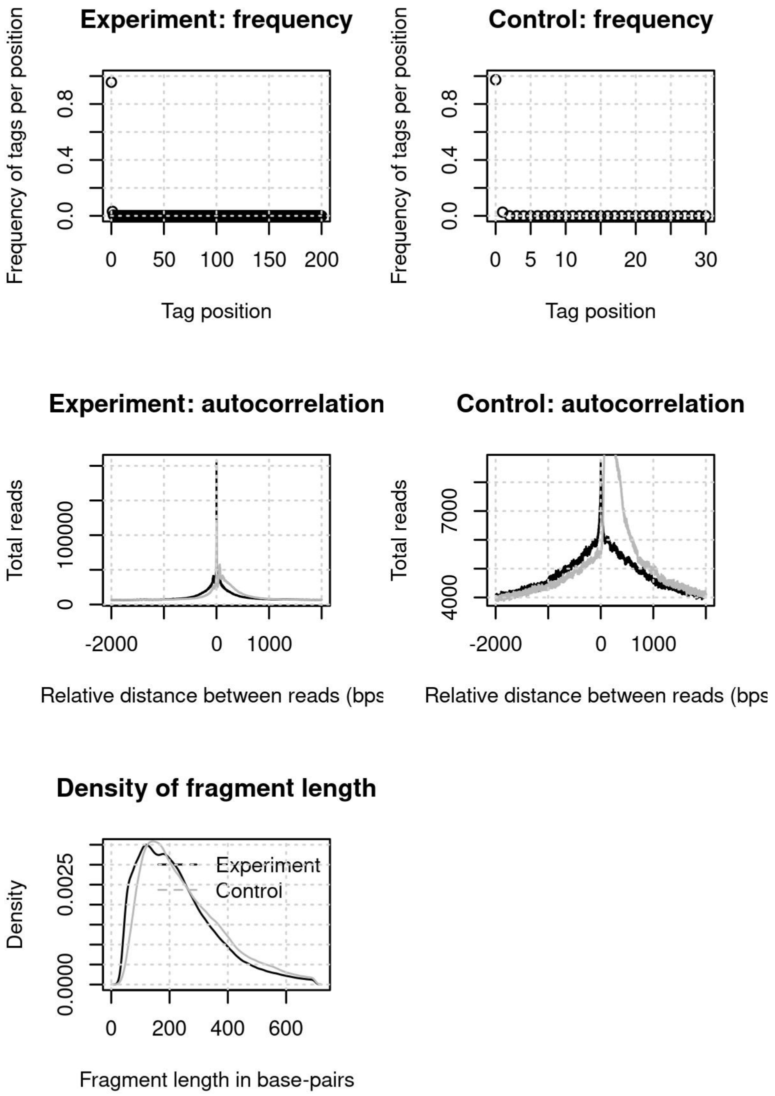

Figure 7.2: This image is output of qcTagDirectories mode.

There are likely three sources of origin of fragments in greenCUT&RUN and CUT&RUN experiments: (1) random breaking of DNA fragments, (2) MNase cutting around histone proteins, and (3) MNase cutting around the protein of interest. For these sources, the fragment size can

vary, ranging from $\geq$ 150 base-pairs to $\leq$ 120 base-pairs, as indicated by Meers et al. (2019)[3]. In the lower panel of the **Figure 7.2**, a bimodal distribution ($\geq$ 150 base-pairs and $\leq$ 120 base-pairs)is observed for the experiment, while there is a continuous distribution for the control. The continuous distribution may be attributed to a higher number of reads resulting from random breakage.

7.3 callPeaks

The greenPipes pipeline accommodates the HOMER and SEACR peak calling algorithms[4,5]. However, the pipeline does not support MACS2. By default, the pipeline utilizes HOMER for peak calling instead of SEACR for reasons outlined above.

In brief, HOMER utilizes tagDirectories, while SEACR employs BED files as input for peak calling (for a detailed understanding of BED file format, refer to `http://genome.ucsc.edu/FAQ/FAQformat`). Consequently, when using HOMER, this mode will depend on `initPeakCalling`. Otherwise, it will rely solely on the `alignment`. If users have employed the default parameters of the `initPeakCalling` mode, then reads are already normalized using spike-in reads number. The removal of possible PCR duplicates is turned off in HOMER by using option `--C 0` of findPeaks. The "findPeaks" is the tool of HOMER and is incorporated in the pipeline. greenPipes is implemented in a way that none of the reads will be removed during BED file generation for peak calling. For SEACR, the pipeline will generate normalized BED files from alignment (BAM) files. For this, it converts BAM files to a fragment file, and then it generates coverage BED files for both the control and experiment. The coverage in control samples will be multiplied by $\left(\frac{C_{\mathrm{S}}}{C_{\mathrm{S}}+C_{\mathrm{h}}}\right) \times \left(\frac{E_{\mathrm{S}}+E_{\mathrm{h}}}{E_{\mathrm{S}}}\right)$ for spike-in normalization. Here, total number of human (or organism of interest) and spike-in reads in experiment are $\mathrm{E_h}$ and $\mathrm{E_S}$ while in control are $\mathrm{C_h}$ and $\mathrm{C_S}$. Following peak calling, any peaks located within the blacklisted regions identified by ENCODE will be excluded from further analysis.

Optional arguments associated with this mode includes `--blackListedRegions`, `--pMethod (homer, seacr)`, `--pStyle (narrow, broad, both)`, `--pFdrHomer`, `--pPvalueHomer`, `--pFcHomer`, `--pDistHomer (fdr, poisson)`, `--pControl (True, False)`, `--pSpike (True, False)`, `--pSeacrMode (stringent, relaxed)`, `--pSeacrThreshold`, `--pOpts` and `--genomeFile`.

Optional arguments associated with this mode include `--blackListedRegions`, `--pMethod (homer, seacr)`, `--pStyle (narrow, broad, both)`, `--pFdrHomer`, `--pPvalueHomer`, `--pFcHomer`, `--pDistHomer (fdr, poisson)`, `--pControl (True, False)`, `--pSpike (True, False)`, `--pSeacrMode (stringent, relaxed)`, `--pSeacrThreshold`, `--pOpts`, and `--genomeFile`. With `--blackListedRegions`, the user can provide the path of a file containing information on ENCODE blacklisted regions in BED format. For users working on the human genome aligned to version hg38, the file hg38-blacklist.v2.bed is available for download at `https://osf.io/ruhj9/`.

Using `--pMethod`, the user can change the peak calling algorithm (HOMER or SEACR). `--pStyle` allows the user to call narrow, broad, or all peaks. Narrow peaks can be called for

[3] Meers et al. (2019). "Improved CUT&RUN chromatin profiling tools" eLife 8:e46314

[4] `http://homer.ucsd.edu/homer/ngs/peaks.html`

[5] Meers, M.P., Tenenbaum, D., Henikoff, S. Peak calling by Sparse Enrichment Analysis for CUT&RUN chromatin profiling. Epigenetics & Chromatin 12, 42 (2019)

transcription factors or histone marks (e.g., H3K4me3, H3K27ac), and broad peaks can be called for certain histone marks (e.g., H3K27me3). The argument *all* identifies both narrow and broad peaks and then merges them together. If the user wishes to modify the peak calling parameters of HOMER (e.g., FDR threshold, p-value, fold changes, type of distribution), options such as `--pFdrHomer`, `--pPvalueHomer`, `--pFcHomer`, and `--pDistHomer` are available. Detailed information on these parameters can be found at http://homer.ucsd.edu/homer/ngs/peaks.html. By default, the greenPipes uses 10^{-3}, 10^{-4}, 4.0, and FDR for FDR threshold, p-value, fold changes, and type of distribution, respectively, which are the default parameters of the HOMER peak calling algorithm.

The `--pControl` is a logical parameter allowing the user to specify whether they want to use the control samples for peak calling. The `--pSpike` is also a logical parameter applicable only when the `--pMethod seacr` is chosen. This option directs the pipeline to perform spike-in normalization during peak calling. Using `--pSeacrMode` and `--pSeacrThreshold`, the user can choose SEACR peak calling mode (relaxed or stringent) and set the threshold for true positive peaks, respectively. The `--pSeacrThreshold` is only crucial when the user is not using control in peak calling. SEACR requires the length of chromosomes (*genome file), which can be downloaded from https://hgdownload.soe.ucsc.edu/downloads.html. For the human genome (version hg38), the user can download the genome file from https://osf.io/ruhj9. We recommend that the user consult the SEACR tutorial at https://github.com/FredHutch/SEACR/ to understand different parameters.

With `--pOpts`, the user can change other parameters of HOMER. If the *genome file is not available for the user's genome of interest, it can be generated using the following method.

```
samtools faidx <input.fa>
cat <input.fa.fai> | cut -f1,2 > <input.genome>

Example:

If path of genome fasta file is /home/sheikh/organism.fa, then:

samtools faidx /home/sheikh/organism.fa

cat /home/sheikh/organism.fa.fai | \
cut -f1,2 > /home/sheikh/organism.genome
```

An example command is as follows:

```
greenPipes \
--inputdir $(pwd)/Fastq \
--inputfile ./Samplesheet.txt \
--libraryType pair \
--outputdir $(pwd) \
--modes callPeak
--blackListedRegions ~/Database/hg38-blacklist.v2.bed
```

In this mode, a single directory named *Peaks* will be created. Peaks related to narrow and broad categories will have the syntax *narrow* and *broad*, respectively. If the user employs the

argument `--pStyle` all, both broad and narrow files will be merged, and files with syntax *all* will be generated. Within the file name syntax, the user can identify the name of the peak calling algorithm. Files *-homer or *-seacr represent the output of the respective calling algorithm. The files *-homer.bed and *-seacr.bed both provide the locations of peaks.

After removing peaks within blacklisted regions, the pipeline generates *removed.bed files. Subsequently, the pipeline removes peaks present within unassembled contigs, generating *Clean.bed files. Throughout all downstream analyses, the *Clean.bed files will be utilized.

```
output folder
|-- Peaks/
    |-- SampleA_all-homer.Clean.bed
    |-- SampleA_broad-homer
    |-- SampleA_broad-homer.bed
    |-- SampleA_broad-homer.Clean.bed
    |-- SampleA_broad-homer.removed.bed
    |-- SampleA_narrow-homer
    |-- SampleA_narrow-homer.bed
    |-- SampleA_narrow-homer.Clean.bed
    |-- SampleA_narrow-homer.removed.bed

.. cut ..
```

7.4 idr

This mode utilizes the pipeline developed by Dr. Karmel Allison, available at http://github.com/karmel/homer-idr. To comprehend IDR or "Irreproducible Discovery Rate," refer to the article published by Li, Qunhua et al (2011)[6][7]. This mode depends on output of the initPeakCalling mode.

In the callPeak mode, by default peaks were called by comparing coverage of experiment with control and background ofthe experiment itself. Here, the threshold of fold changes (default value is equal to 4) and p-values (default value is equal to 0.0001) are set in a way that only true-positive peaks are identified, while noise or false positive peaks can be filtered out. Let's assume that a peak has a fold changes of 3.9, this peak will be filtered out, although it is a true peak. Users can decrease the threshold value to include peaks with low fold changes by using options `--pPvalueHomer` and `--pFcHomer`, but it will also increase the number of false-positive peaks. In this scenario, users can use this (IDR) mode if they have replicates of the samples, because IDR framework will measure the reproducibility of peaks among replicates and provide highly stable thresholds based on reproducibility (http://github.com/karmel/homer-idr).

Options associated with this mode includes: `--idrExprs`, `--idrCtrl`, `--idrName`, `--idrExprSpike`, `--idrCtrlSpike`, `--idrControl (True,False)`, `--idrSpike (True,False)`, `--idrStyle (factor,histone)`, `--idrOutput`, `--idrMethod (homer)`.

With `--idrExprs` and `--idrCtrl`, users can give the path of tagDirectories of replicates as comma-separated values for experiment and control samples, respectively. The user

[6] https://statistics.berkeley.edu/sites/default/files/tech-reports/790.pdf

[7] Qunhua Li, James B. Brown, Haiyan Huang, Peter J. Bickel, "Measuring reproducibility of high-throughput experiments," Ann. Appl. Stat. 5 (3) 1752 - 1779, 2011

can separate different experiments with ";". For example, suppose that the user has two replicates (rep1 and rep2) for two different experiments (exp1 and exp2), and associated controls (ctrl1 and ctrl2). The user aims to find IDR peaks for exp1 as well as exp2. Then, they can provide experimental tagDirectories by `--idrExprs tagDir/exp1\_rep1, tagDir/exp1\_rep2; tagDir/exp2\_rep1, tagDir/exp2\_rep2` and control tagDirectories by `--idrControl tagDir/ctrl1\_rep1, tagDir/ctrl1\_rep2; tagDir/ctrl2\_rep1, tagDir/ctrl2\_rep2`. With `--idrName` the user can provide the name of the experiments e.g. `--idrName exprIDR1;exprIDR2\verb`.

In case when user does not want to normalize IDR peak finding with spike-in, they can be switch off normalization by using `--idrSpike False`. Similarly, when the user do not want to use control, they can switch it off by using `--idrControl False`. By default, both options are active, indicating that the pipeline will call IDR peaks against control and perform spike-in normalization. If `--idrSpike` is true, provide bam files of spike-in for experiment and control with `--idrExprSpike` and `--idrCtrlSpike`, respectively. These files are present in the spike-in folder and they are generated during `alignment` mode. greenPipes pipeline will use this file to perform normalization using equation 2.

Narrow and broad IDR peaks can be called using option: `--idrStyle`. If for experiment1 you want to call narrow peaks but for experiment 2 broad, then specify as follows: `--idrStyle factor;histone`. Specify prefix of output IDR peak file by using `--idrOutput exprIDR1;exprIDR2`. With `--idrMethod`, the user can specify peak calling method e.g. HOMER or MACS2. Ignore this option as MACS2 is not supported by greenPipes. This mode also needs information of the ENCODE black listed regions also. Specify this by using `--blackListedRegions`.

```
greenPipes \
--libraryType pair \
--outputdir $(pwd) \
--modes idr \
--blackListedRegions  ~/Database/hg38-blacklist.v2.bed \
--idrExprs   tagDir/exp1\_rep1,tagDir/exp1\_rep2;\
             tagDir/exp2\_rep1,tagDir/exp2\_rep2\
--idrControl tagDir/ctrl1\_rep1,tagDir/ctrl1\_rep2;\
             tagDir/ctrl2\_rep1,tagDir/ctrl2\_rep2 \
--idrSpike True \
--idrControl   True \
--idrStyle factor;histone \
--idrOutput exprIDR1;exprIDR2 \
--idrMethod homer \
--idrExprSpike spike-in/exp1\_repl1.bam,spike-in/exp1\_repl2.bam;\
               spike-in/exp2\_repl1.bam,spike-in/exp2\_repl2.bam \
--idrCtrlSpike spike-in/ctrl1\_repl1.bam,spike-in/ctrl1\_repl2.bam;\
               spike-in/ctrl2\_repl1.bam,spike-in/ctrl2\_repl2.bam
```

A single directory *idr_homer* will be generated in this mode. For each experiment separate directories will exist within idr_homer. File TotalExperiment.peaks-top-set.txt is output of the IDR calling algorithm. This file includes different values associated with the IDR peaks. Please go to `http://github.com/karmel/homer-idr` to understand these values in detail. The TotalExperiment.peaks-top-set.bed contains the same information but format of file is BED. After

removing peaks falling within blacklisted regions, greenPipes generates TotalExperiment.peaks-top-set.removed.bed files. In the next step, peaks present within unassembled contigs are removed by greenPipes and TotalExperiment.peaks-top-set.Clean.bed are generated. In all downstream analysis, TotalExperiment.peaks-top-set.Clean.bed will be used. All intermediate files and images will be present in the folder narrowpeaks, replicate_comparisons, pseudorep_comparisons and pooled_comparisons. To understand files in this folder and to understand images present in plots folder, please visit https://github.com/karmel/homer-idr.

```
output folder
|-- idr_homer/
    |-- exprIDR1
       |-- plots
           |-- Replicate_comparison-plot.ps
           |-- Pseudorep_comparison-plot.ps
           |-- Pooled_pseudorep_comparison-plot.ps
       |-- narrowpeaks/
       |-- replicate_comparisons/
       |-- pseudorep_comparisons/
       |-- pooled_comparisons/
       |-- TotalExperiment.peaks-top-set.txt
       |-- TotalExperiment.peaks-top-set.bed
       |-- TotalExperiment.peaks-top-set.removed.bed
       |-- TotalExperiment.peaks-top-set.Clean.bed
    |-- exprIDR2
       |-- plots
       |-- narrowpeaks/
       |-- replicate_comparisons/
       |-- pseudorep_comparisons/
       |-- pooled_comparisons/
       |-- TotalExperiment.peaks-top-set.txt
       |-- TotalExperiment.peaks-top-set.bed
       |-- TotalExperiment.peaks-top-set.removed.bed
       |-- TotalExperiment.peaks-top-set.Clean.bed

.. cut ..
```

Chapter 8

Annotation

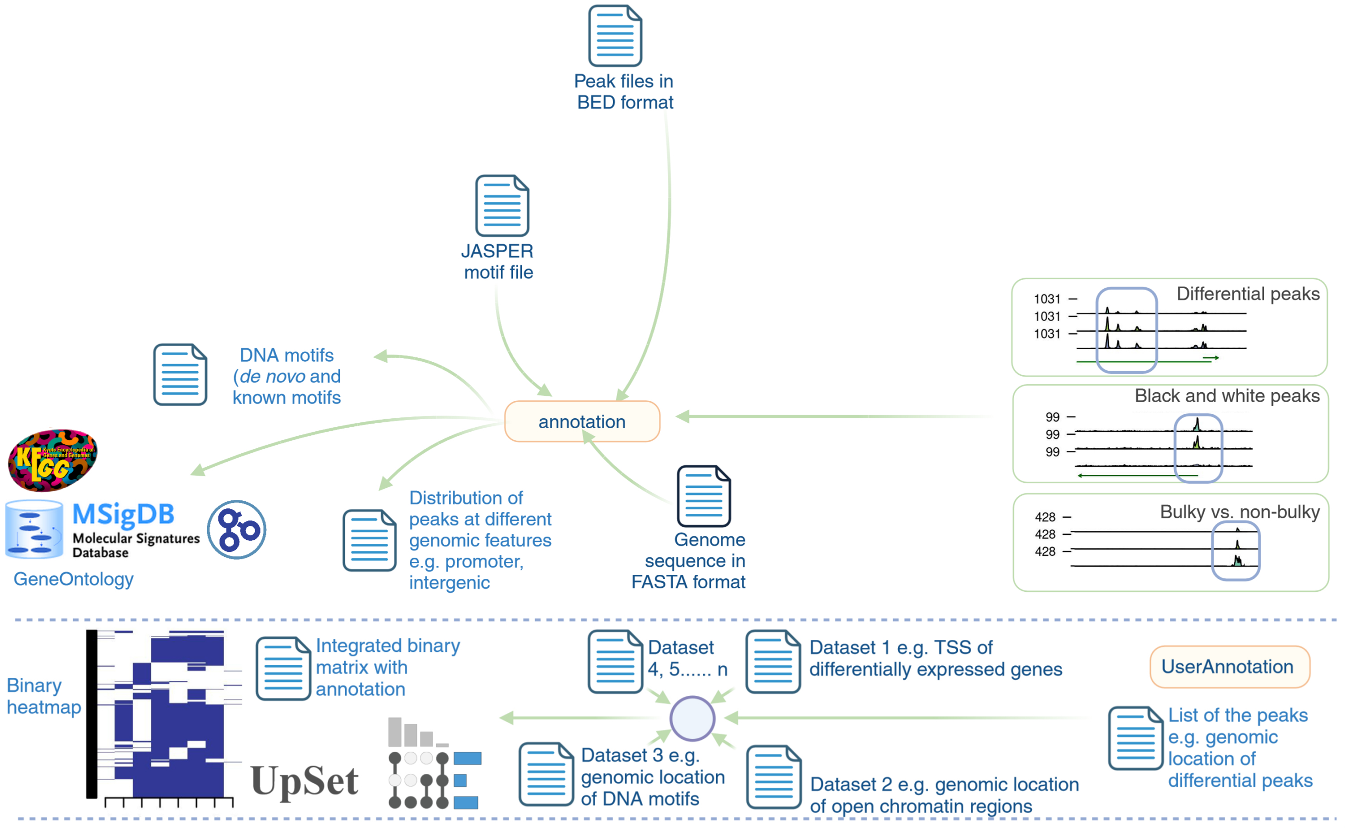

Figure 8.1: The workflow of the annotation and UserAnnotation mode of the greenPipes.

8.1 annotation

After peak calling, the next logical step is annotation. The pipeline calculates the enrichment of known motifs within peaks, performs gene ontology analysis, and conducts genomic annotations. Additionally, it engages in motif discovery using MEME, which is independent of known motifs.

Optional arguments associated with this mode include `--annpeakFiles`, `--annPrefix`, `--sFasta`, and `--cGVersion`.

greenPipes offers users the flexibility to manually provide peak files in BED format using the `--annpeakFiles` option. Alternatively, the pipeline will automatically search for *.Clean.bed

files in the Peaks and idr_homer folders. If user chooses to provide the BED file manually via the `--annpeakFiles` option, it should be supplied by the output file prefixes as comma-separated values using the `--annPrefix` option.

greenPipes extracts sequences using peak location information and searches for DNA motif sequences. For this, it requires information about the genome version and the DNA sequence file, which users can provide using `--cGVersion` and `--sFasta`. The `--cGVersion` option requires the genome version, and `--sFasta` needs the genome sequence file in the FASTA format. By default, the pipeline uses the hg38 genome version and supports various genomes, including human (hg18, hg19, hg38), mouse (mm8, mm9, mm10), rat (rn4, rn5, rn6), frog (xenTro2, xenTro3), zebrafish (danRer7), drosophila (dm3), *C. elegans* (ce6, ce10), *Saccharomyces cerevisiae* (sacCer2, sacCer3), *Schizosaccharomyces pombe* (ASM294v1), arabidopsis (tair10), and rice (msu6). For genomic annotations and gene ontology, the pipeline requires information about the genome version.

The annotation libraries for one of the supported genomes has been already set-up during installation of pipeline. When users have their own reference genome, they can follow the tutorial at http://homer.ucsd.edu/homer/introduction/update.html to add the annotation library for their genome of interest in the greenpipes conda environment. This library will be used by HOMER tools which are integrated in the greenPipes. For annotation, the pipeline utilizes the `annotatePeaks.pl` function of HOMER. Refer to http://homer.ucsd.edu/homer/ngs/annotation.html and http://homer.ucsd.edu/homer/ngs/advancedAnnotation.html to gain a detailed understanding of this function.

An example command for paired-end reads is as follows:

```
greenPipes \
--inputdir $(pwd)/Fastq \
--inputfile ./Samplesheet.txt \
--libraryType pair \
--outputdir $(pwd) \
--modes annotation \
--sFasta ~/Database/hg38/GenCode/GRCh38.p13.fa
```

The user can also provide their own peak files (in BED format):

```
greenPipes \
--inputdir $(pwd)/Fastq \
--inputfile ./Samplesheet.txt \
--libraryType pair \
--outputdir $(pwd) \
--modes annotation \
--sFasta ~/Database/hg38/GenCode/GRCh38.p13.fa \
--annpeakFiles $(pwd)/Peaks/SampleA_narrow-homer.Clean.bed,\
       $(pwd)/Peaks/SampleB_narrow-homer.Clean.bed \
--annPrefix SampleA,SampleB
```

Three different directories: (1) Annotation, (2) GeneOntology and (3) Motifs will be generated in this mode.

```
output folder
|-- Annotation/
    |-- SampleA_narrow-homer-annotation.txt
    |-- SampleA_narrow-homer-annStats.txt
.. cut ..

|-- GeneOntology/
    |-- SampleA_narrow-homer_GO/
        |-- biocyc.txt
        |-- biological_process.txt
        |-- cellular_component.txt
        |-- chromosome.txt
        |-- cosmic.txt
        |-- gene3d.txt
        |-- geneOntology.html
        |-- gwas.txt
        |-- interactions.txt
        |-- interpro.txt
        |-- kegg.txt
        |-- lipidmaps.txt
        |-- molecular_function.txt
        |-- msigdb.txt
        |-- pathwayInteractionDB.txt
        |-- pfam.txt
        |-- prints.txt
        |-- prosite.txt
        |-- reactome.txt
        |-- smart.txt
        |-- smpdb.txt
        |-- wikipathways.txt
.. cut ..

|-- Motifs/
    |-- Homer/
        |-- SampleA_narrow-homer/
            |-- knownResults.html
            |-- knownResults.txt
.. cut ..

            |-- knownResults/
                |-- known1.logo.svg
                |-- known1.motif
.. cut ..

    |-- Meme/
        |-- SampleA_narrow-homer.fa
        |-- SampleA_narrow-homer_meme/
            |-- meme.txt
            |-- meme.html
```

```
            |-- meme.xml
            |-- logo1.eps
            |-- logo1.png
.. cut ..

        |-- SampleA_narrow-homer_tomtom
            |-- tomtom.html
            |-- tomtom.tsv
            |-- tomtom.xml
```

In the Annotation folder, the user will find two files associated with one peak file. The *-annotation.txt file includes genome annotation (TSS or transcription start site [-1 kb to +100bp], TTS or transcription termination site [-100 bp to +1 kb], CDS or coding sequence Exons, UTR or untranslated regions, Introns and Intergenic) and *annStats.txt file includes annotation enrichment result.

In the GeneOntology folder, the user will find a *_GO folder for each peak file. HOMER performs analysis for different ontologies. Among different ontologies, we prefer msigdb because most of our studies is to characterize disease-associated mutations. For example when a mutation is located in a protein-protein interaction domain, in a simple scenario users will observe loss of peaks in mutants. The subsequent step involves identifying interacting proteins, which can be achieved by comparing motif enrichment between wild type and mutant proteins, with information available in the Motifs folder. However, although an important step, the observation of DNA motifs of several transcription factors may not be very helpful in narrowing down potential 'interacting proteins'. The msigdb database, developed by the Broad Institute, contains lists of genes that are either upregulated or downregulated upon the knockout or knockin of other genes. Utilizing msigdb can help to narrow down potential 'interacting proteins' based on real data, even if it originates from different cell lines or tissues. Or, users can perform IP-based mass spectrometry to identify interacting proteins. Note that p-values reported in the gene ontology analysis are not multiple-test corrected.

greenPipes identifies enriched DNA motifs using two different tools: HOMER http://homer.ucsd.edu/homer/ngs/peakMotifs.html and MEME https://meme-suite.org/meme/doc/meme.html?man_type=web, and stores the results in the Homer and Meme folders, respectively. greenPipes searches for DNA motifs within 200 base pairs around the center of peak and uses default parameters in case of HOMER. In case of MEME, greenPipes find motifs within the genomic location of peak. All default parameters are used in MEME, except it calls (1) 10 motifs within 6 to 15 base-pairs, (2) uses zoops mod for motif distribution and (3) consider both the given strand and the reverse complement strand when searching for motifs in a complementable alphabet (i.e. DNA). After finding motifs, greenPipes uses tomtom tool of MEME suite to find whether identified motifs is already reported or not. For this, pipline use JASPAR 2018 CORE datasets which has non-redundant DNA motifs. Path of this file is *greenPipe/data/JASPAR2018_CORE_non-redundant.meme.

8.2 UserAnnotation

In some cases, users may have an interest in annotating peaks using their own annotation datasets. For example, in order to determine whether peaks overlap with active histone marks like H3K4me3, H3K27ac, or open chromatin regions. This mode is specifically designed to ad-

dress such scenarios. Users can utilize this mode to annotate peaks using various annotation files, either from the provided collection at https://osf.io/ruhj9/ or their custom annotation BED files.

Options associated with this mode are: --annpeakFiles, --annFiles, --annSize, --annName and --annPrefix.

Through --annpeakFiles the user can provide peaks in BED format which should be annotated. Multiple peak files can be given as comma-separated values e.g. --annpeakFiles /path/peakFile1.bed,/path/peakFile2.bed. If user did not manually provide the peak files in BED format using --annpeakFiles, greenPipes will automatically search for *.Clean.bed file in Peaks and idr_homer folder.

With --annFiles one can provide bed files of annotation e.g. genomic location of active histone mark H3K4me3, cJUN/FOS motif location etc. The user can provide more than one annotation bed files as comma-separated values e.g. f1.bed,f2.bed.

With --annSize, users can specify the maximum distance between the boundary of a peak (--annpeakFiles) and its associated annotation (--annFiles). Users have the flexibility to provide distinct maximum distances for each annotation file. For instance, when a user wishes to set a maximum distance of 500 base pairs for H3K4me3 and 100 base pairs for cJUN/FOS, they can use the format --annSize 500,100. When the user provides only one argument for --annSize e.g. --annSize 200 for more than one annotation file, then greenPipes automatically assigns a 200 base pair threshold for each annotation file.

With --annName one can provide name of the annotation e.g. if user are using --annFiles /path/H3K4me3.bed,/path/JUN.bed, then they should use --annName H3K4me3,JUN.

The --annPrefix is neccessary if user is providing their own peak files. This will be used to generate output files. When greenPipes automatically collect *.Clean.bed file from the Peaks and idr_homer folder, then it will automatically determine the prefix of the output files.

In the field of epigenomics, many groups want to correlate the active or inactive histone marks e.g. H3K27ac or H3K9me3 or histone modifiers e.g. KDM6A or KDM6C or trancription factors e.g. SMAD4 with gene expression levels. Sufficient number of peaks of these tagets are present at enhancers. One can call peaks for histone marks or histone modifiers or transcription factors using greenCUT&RUN or CUT&RUN technique and correlate this to mRNA expression level. However, this only works well when the peak(s) are close to the transcription start site of a gene. To find correlation of the long range effect of these factors on mRNA expression level, users need long range interaction information. One can use enhancer-gene interaction data set (uploaded to https://osf.io/ruhj9/) as a proxy if long range interaction datasets is not available for their cell or tissue of interest.

An example command for paired-end reads is as follows:

```
annDir="~/Annotation/"

greenPipes \
--inputfile ./Samplesheet.txt \
--outputdir $(pwd) \
--modes UserAnnotation \
--annFiles $annDir/GSE121840.bed,\
           $annDir/ENCFF392EDT.bed,\
           $annDir/ENCFF862LUQ.bed,\
           $annDir/ENCFF510LKP.bed,\
           $annDir/Enhancer.bed,\
           $annDir/ENCFF636PWI.bed \
```

```
    --annSize   300,300,400 \
    --annName   ATAC,H3K27ac,H3K4me3,H3K9ac,Enhancers,PolII
```

A single directory UserAnnotation will be generated in output folder. For each sample four files *.totalOutput.txt, *totalMatrix.txt, *Distribution.jpeg, *UpSetPlot.jpeg will be generated. The *.totalOutput.txt contains information of overlap of peaks in a flat contingency table format. The last two columns are counts while other columns shows presence (denoted as 1) or absent of factor (0). The *totalMatrix.txt will contain information of peaks and a binary matrix in which "1" means true for overlap while "0" means false for the overlap. *Distribution.jpeg and *UpSetPlot.jpeg is pictorial image of binary matrix of *totalMatrix.txt and table of *.totalOutput.txt, respectively (**Figure 8.2**).

```
output folder
|-- UserAnnotation/
    |-- SampleA_narrow-homer.txt.Distribution.jpeg
    |-- SampleA_narrow-homer.txt.totalMatrix.txt
    |-- SampleA_narrow-homer.txt.UpSetPlot.jpeg

.. cut ..
```

Table 8.1: Format of *.totalMatrix.txt

seqnames	start	end	width	strand	ATAC	H3K27ac	H3K4me3	H3K9ac	Enhancers	PolII	..
chr17	47895954	47896147	194	*	1	1	1	1	1	1	..
chr19	11374690	11374883	194	*	1	0	1	1	1	1	..
chr9	137241039	137241232	194	*	1	1	1	0	1	1	..
chr17	40417912	40418105	194	*	1	1	1	1	1	0	..
..	..	..	..	..	..	..	..	..	..	..	..
if overlap then value is equal to 1 else 0											

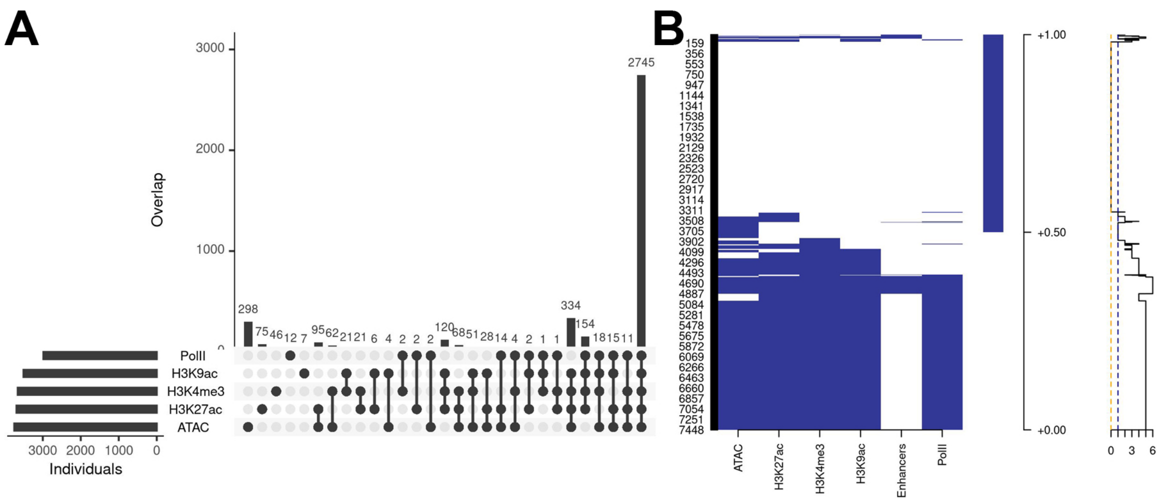

Figure 8.2: A. UpSetPlot to show the overlap of different annotations of user provided peaks. Most of the SampleA peaks overlap with active histone marks, bound by pol II and are open chromatin regions. B. Image of *totalMatrix.txt file. Hierarchical clustering will be performed by greenPipes to cluster the peaks on basis of their annotation. On the right side, the line graph shows how many factors are overlapping with the peaks. The dashed black line is at 1 while orange line is at 0. This figure suggests that ≈40% peaks are not overlapping with any factors. Here, the user can use the annotation datasets available at https://osf.io/ruhj9/ to identify other factors overlapping with these ≈40% peaks to obtain novel insights.

Table 8.2: Flat contingency table format of *.totalMatrix.txt. For example first line shows that 12 peaks of SampleA are as such that they overlap with PolII but not with any other factors.

Overlap = 1; Nonoverlap = 0						Count	
						Pol II	
..	ATAC	H3K27ac	H3K4me3	H3K9ac	Enhancers	0	1
..	0	0	0	0	0	3204	12
..	-	-	-	-	1	25	0
..	-	-	-	1	0	7	0
..	-	-	-	-	1	0	0
..	-	-	1	0	0	44	2
..	-	-	-	-	1	2	0
..	-	-	-	1	0	18	1
..	-	-	-	-	1	3	0
..	-	1	0	0	0	71	2
..	-	-	-	-	1	4	0
..	-	-	-	1	0	5	2
..	-	-	-	-	1	1	0
..	-	-	1	0	0	21	1
..	-	-	-	-	1	0	0
..	-	-	-	1	0	111	135
..	-	-	-	-	1	9	19
..	1	0	0	0	0	290	2
..	-	-	-	-	1	8	0
..	-	-	-	1	0	4	0
..	-	-	-	-	1	0	0
..	-	-	1	0	0	60	3
..	-	-	-	-	1	2	1
..	-	-	-	1	0	48	15
..	-	-	-	-	1	3	3
..	-	1	0	0	0	92	13
..	-	-	-	-	1	3	1
..	-	-	-	1	0	27	15
..	-	-	-	-	1	1	0
..	-	-	1	0	0	67	11
..	-	-	-	-	1	1	0
..	-	-	-	1	0	310	2431
..	-	-	-	-	1	24	314
..	..	..	..	..	..	..	..

Chapter 9

Comparison of peaks

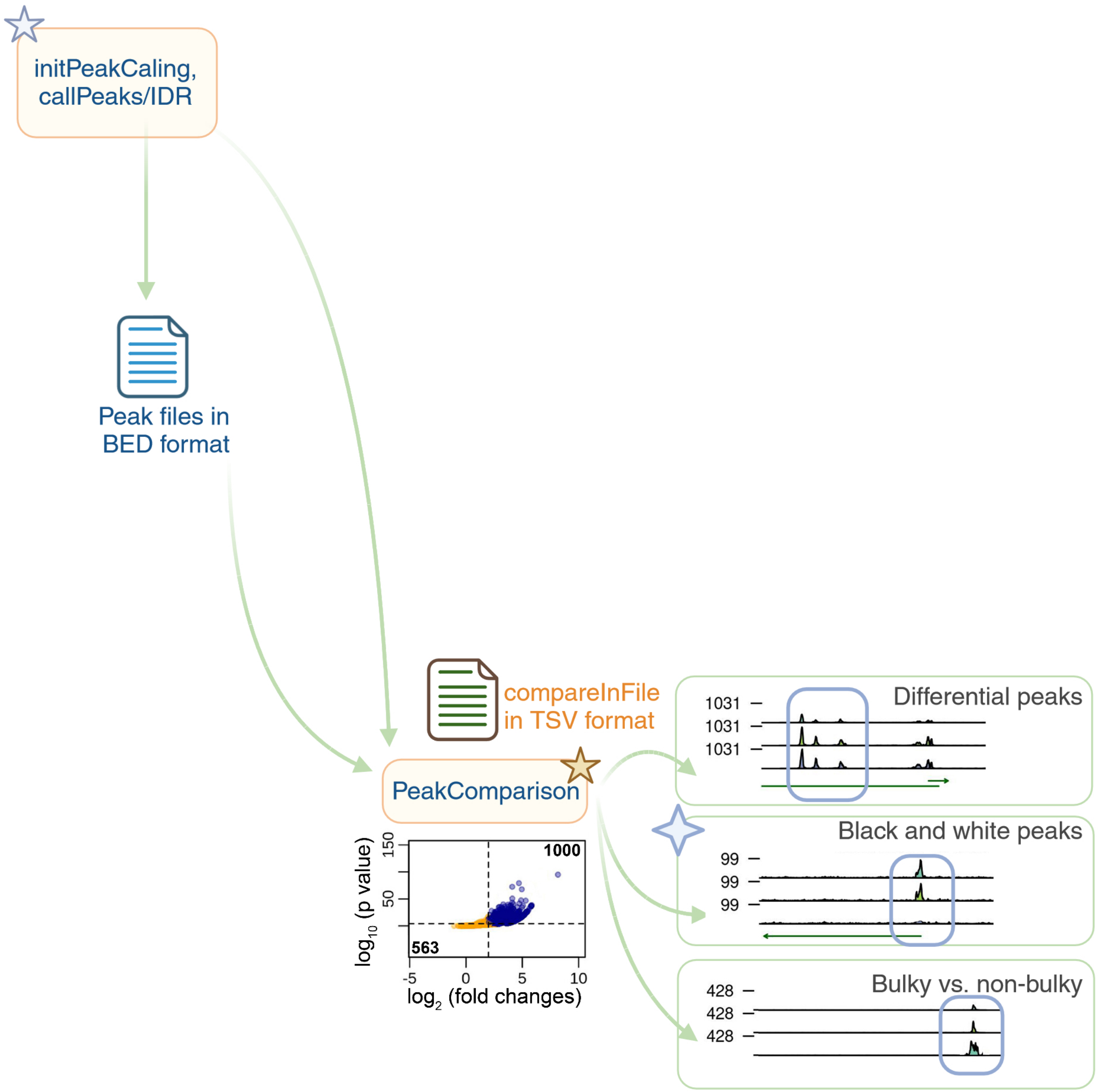

Figure 9.1: The workflow of the PeakComparison mode of the greenPipes.

In this mode, greenPipes will (1) find differential peaks, (2) generate V-plot, (3) identify black and white peaks after simulation, (4) identify bulky and non bulky peaks (this will be an interesting

analysis if user is working with large protein complexes) and (5) annotate peaks. The user must create a file following the instructions outlined in the section Sheet for peak comparison among different experiments. In the next subsection, we will discuss about the different analysis performed in this mode, and will explain the output files. In last, we will describe the options available in this mode. All files and folders associated with this mode will be stored in folder *comparePeaks* in output folder mentioned by `--outputdir`.

9.1 Differential peaks

A common practice is to compare peaks among experiments. Many articles opt for Venn diagrams as the primary tool to illustrate overlapping and non-overlapping peaks among experiments. However, this approach can be biased until IDR-based peak calling is performed. The bias stems from the fact that numerous peak calling tools, including greenPipes, adhere to stringent criteria for defining peaks. For instance, using the default parameters of HOMER, only regions with peak intensity greater than 4 compared to controls and a p-value < 0.0001 are considered peaks. Consequently, peaks with a fold change of 3.99 or lower are excluded from the final list due to these strict criteria. It is important to emphasize that the presence of a peak may not be accurately represented in a Venn diagram when it was filtered out during peak calling. Therefore, relying on a Venn diagram to depict overlapping and non-overlapping peaks may not be the most reliable approach.

Rather than utilizing a Venn diagram, greenPipes employs differential peak calling to identify the true positive number of peaks that differ among experiments. This process involves the use of the HOMER tool, with the tagDirectories of the experiments serving as inputs. So this mode will depend on the successful completion of the `initPeakCalling` mode. Before initiating the calling of differential peaks, greenPipes performs normalization on tagDirectories based on the spike-in.

Assume that the total number of human (or the organism of interest) and the spike-in reads in experiment a are Ea_h and Ea_s while in experiment b are Eb_h and Eb_s. Then, total spike-in normalized reads for experiment a (Ea_T) will be equal to $Ea_h \times \left(\frac{Ea_s}{Ea_s+Ea_h}\right) \times \left(\frac{Eb_s+Eb_h}{Eb_s}\right)$ and experiment b (Eb_T) will be equal to Eb_h. The user also has the option to generate a Venn diagram (refer to the options provided at the end of this chapter).

greenPipes will create *Peaks* folder in *comparePeaks* and will generate two files for a single experiment. The *-vs-*-{p-value}-{fold change}.txt file contains information about differential peaks (p-value and fold change in name of the file equal to 0.0001 and 4.0 by default or equal to values provided by `--rdPvalue` and `--rdFoldChange`). The *-vs-*-allPeaks.txt file contains fold change and p-value for all given peaks. If user is not satisfied with the default `--rdPvalue` and `--rdFoldChange`, then there is no need to run greenPipes again, they can open *-vs-*-allPeaks.txt in excel or in R, and filter peaks on basis of their choice of p-value and fold changes. greenPipes generate *all* file for this purpose. See the heading *Differentially Bound Peaks* at `http://homer.ucsd.edu/homer/ngs/mergePeaks.html` to understand format of output files.

```
output folder
|-- comparePeaks/
    .. cut ..
    |-- Peaks/
        |-- SampleA-vs-SampleB-allPeaks.txt
        |-- SampleA-vs-SampleB-0.0001-4.0.txt
```

```
.. cut ..
```

After finding true positive differential peaks, pipeline will generate a V plot plot similar to **Figure 9.2**. Image file for each samples will be stored in folder comparePeaks/ImageBhujangasanaPlot.

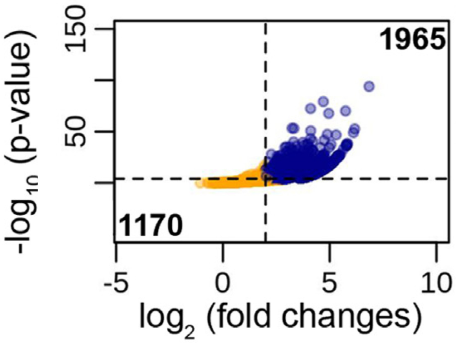

Figure 9.2: An example of V plot generated by greenPipes. The up-right number 1965 denotes the number of true positive peaks different in one experiment compared to the other.

9.2 Black and white

greenPipes facilitates the identification of experiment-specific peak gains, where a peak is exclusively observed in a particular experiment. We call these black and white (BW) peaks. greenPipes categorizes a peak as differential BW peak when the coverage at this location equals the maximum achievable background coverage in one experiment and is $\geq$4 in another experiment.

To find the maximum achievable coverage in the background, greenPipes performs simulation and assume that coverage will follow a normal distribution. It selects $\approx$40,000 genomic regions of same length as of the peaks and extracts spike-in normalized coverage. Then, it calculates median and median absolute deviation of coverage. For examples, suppose the user has $1...N$ random location and associated coverage is $x_1...x_N$. The median of this distribution is denoted as $\tilde{x}$. To use median absolute deviation as a consistent estimator, greenPipes uses following formula:

$$\frac{1}{\phi^{-}1 \times \frac{3}{4}} \times median_{(n=1..N)}(|x_n - \tilde{x}|)$$

...equation 3

The value $\frac{1}{\phi^{-}1 \times \frac{3}{4}}$ is $\approx$1.482602. Now greenPipes finds the upper cut-off value for the background coverage. To cover 99% area of the distribution, greenPipes multiplies equation 3 with 3 and adds median value to find the upper cut-off value of the background coverage. In lay man language, if we bin all genome location in the peak size, 99% locations will be having coverage $\leq$ cut-off value (bw_{cutoff}). When the peak has a coverage $\leq bw_{cutoff}$ in one experiment and $\geq (4 \times bw_{cutoff})$ in another experiment, it is denoted as a BW peak.

greenPipes will generate a single folder *BlackAndWhite* in *comparePeaks folder*. For each sample, it will generate three files. File random-*.bed will contain all random location, randomReadCount-*.txt will contain spike-in normalized reads and *-vs-*-{p-value}-{fold

change}.txt will contain information about BW peaks (p-value and fold change in name of the file equal to 0.0001 and 4.0 by default or equal to values provided by `--rdPvalue` and `--rdFoldChange`).

```
output folder
|-- comparePeaks/
    .. cut ..
    |-- BlackAndWhite/
        |-- random-SampleA.bed
        |-- randomReadCount-SampleA.txt
        |-- SampleA-vs-SampleB-0.0001-4.0.txt

.. cut ..
```

9.3 Bulky *vs.* nonBulky

For researchers focusing on large protein complexes, the analysis of Bulky *vs.* nonBulky is quite valuable. Consider a scenario where a user is studying a DNA-binding protein within a complex that potentially interacts with histone molecules. Initially, the DNA-binding protein is recruited, followed by the recruitment of other components of the complex. Now, when the mutant protein can still bind to DNA but is unable to recruit its complex partners, differential peaks may not be apparent between mutant and wild type protein. It is because protein can bind to DNA with or without the mutation. Nevertheless, theoretically, the size of the fragments resulting from the binding of mutant proteins will differ from those of wild-type proteins due to their distinct interactions with other proteins.

Therefore, by segregating reads into those with a length of $\leq$ 150 bps and those with a length of $>$ 150 bps, calculating coverage, and subsequently conducting a differential peak analysis, we can identify these additional differential peaks. In this context, peaks with more reads having a fragment size of $\leq$ 150 bps are referred as nonBulky, while peaks with a fragment size of $>$ 150 bps are refered as Bulky.

greenPipes identifies these peaks and annotates them using `annotation` mode. A single folder `BulkyvsNonBulky` will be generated in `comparePeaks` folder. For each comparison, 12 files and 4 folders will be generated. The *150S* files and folders are related to fragment size $\leq$ 150 bps while *150L* files and folders are related to fragment size $>$ 150 bps. TagDir is tagDirectories containing reads and coverage information after spike-in normalization. The SampleA-vs-SampleB-{fragment size}-{p-value}-{fold changes}.txt and SampleA-vs-SampleB-{fragment size}-{p-value}-{fold changes}.bed contains differential peaks information. These are intermediate files. The user should focus on the *-nonBulky-0.0001-4.0.bed and *-Bulky-0.0001-4.0.bed files. These files encompass details about peaks that were not previously identified when considering reads of all fragment sizes. These peaks differ in terms of the distribution of fragment sizes among experiments, while their coverage remains consistent.

```
output folder
|-- comparePeaks/
    .. cut ..
    |-- BulkyvsNonBulky/
        |-- TagDir_150S_SampleA
```

```
            |-- TagDir_150L_SampleA
            |-- TagDir_150S_SampleB
            |-- TagDir_150L_SampleB
            |-- SampleA-vs-SampleB-150S-0.0001-4.0.txt
            |-- SampleB-vs-SampleA-150S-0.0001-4.0.txt
            |-- SampleA-vs-SampleB-150L-0.0001-4.0.txt
            |-- SampleB-vs-SampleA-150L-0.0001-4.0.txt
            |-- SampleA-vs-SampleB-150S-0.0001-4.0.bed
            |-- SampleB-vs-SampleA-150S-0.0001-4.0.bed
            |-- SampleA-vs-SampleB-150L-0.0001-4.0.bed
            |-- SampleB-vs-SampleA-150L-0.0001-4.0.bed
            |-- SampleA-vs-SampleB-nonBulky-0.0001-4.0.bed
            |-- SampleA-vs-SampleB-Bulky-0.0001-4.0.bed
            |-- SampleB-vs-SampleA-nonBulky-0.0001-4.0.bed
            |-- SampleB-vs-SampleA-Bulky-0.0001-4.0.bed

    .. cut ..
```

9.4 Motifs, annotation and gene ontology

All differential and black-and-white peaks will be automatically annotated, and corresponding files and folders will be generated following the same pattern as in the Annotation mode. If user want to annotate these differential peaks by using their own annotation files in BED format, they can use `UserAnnotation` mode.

9.5 Options

Options associated with this mode includes: `--overFiles`, `--overDist`, `--compareInfile`, `--rdPvalue`, `--rdFoldChange`, `--rdSize`, `--rdPeak`, `--rdOther`, `--cGVersion`, `--sFasta` and `--genomeFile`.

Options –overFiles and –overDist are associated with generation of a Venn-diagram. greenPipes uses a script available at `https://github.com/stevekm/Bioinformatics/blob/master/HOMER_mergePeaks_pipeline/HOMER_mergePeaks_multiVenn/multi_peaks_Venn.R` with slight modification for generating venn-diagram pictures.

With `--overFiles`, the user can provide their own peak files in BED format as comma-separated values e.g dir1/dir2/peak1.bed,dir1/dir2/peak2.bed. Give the full path of file. If not given, greenPipes automatically finds the IDR peaks or peaks from the output folder.

With `--overDist`, the user can specify distance from center of peak, which will be used to find overlap.

With `--compareInfile`, the user can provide file which is prepared as instructed in the section Sheet for peak comparison among different experiments.

With `--rdPvalue` and `--rdFoldChange`, the user can specify p-value and fold changes to find differential peaks. Default values are 0.0001 and 4, respectively.

With `--rdSize`, the user can specify the size from the center of peak. This size will be used to compare two experiments. For transcription factor analysis, we suggest utilizing a region

size of 1000 base pairs, whereas for histone modifications, a region size of 3000 base pairs is recommended.

With `--rdPeak`, the user can provide a list of the peak files for which differential peaks will be calculated. In fact, greenPipes searches the peak file automatically in the output folder. If user want to provide their own peak file, then, for each line of `--compareInfile`, the user can provide `--rdPeak` file as like this: sample1_condition1:sample1_condition2,sample2_condition1:sample2_condition2. If user want to compare only for same peak file then they can use: peak1:peak2.

With `--rdOther`, user can add additional parameters in peak comparison. Provide it as comma-separated value in large bracket e.g [-xyz,xx,-yy,kk]. To find additional option type: `getDifferentialPeaks --help` in your computer's terminal or go to HOMER website http://homer.ucsd.edu/homer/ngs/mergePeaks.html and see commands in header *Command Line options for getDifferentialPeaks*.

For the annotations, greenPipes needs the genome version, fasta file and genome file. Specify this with `--cGVersion`, `--sFasta` and `--genomeFile`, respectively. For human genome (version hg38), use *genomeFile file uploaded to https://osf.io/ruhj9. If *genome file is not available for the genome of interest of user, it can be generated using following command in terminal:

```
samtools faidx <input.fa>
cat <input.fa.fai> | cut -f1,2 > <input.genome>

Example:

If path of genome fasta file is /home/sheikh/organism.fa, then:

samtools faidx /home/sheikh/organism.fa

cat /home/sheikh/organism.fa.fai | \
cut -f1,2 > /home/sheikh/organism.genome
```

An example command to run this mode is as follows:

```
greenPipes \
--inputfile ./Samplesheet.txt \
--inputdir $(pwd)/Fastq \
--outputdir $(pwd) \
--modes PeakComparison \
--blackListedRegions ~/Database/hg38-blacklist.v2.bed \
--sFasta ~/Database/hg38/GenCode/GRCh38.p13.fa \
--genomeFile ~/Database/hg38/GRCh38.p13.genome \
--cGVersion hg38
--compareInfile ./compare.txt
```

Chapter 10

Visualization of peaks

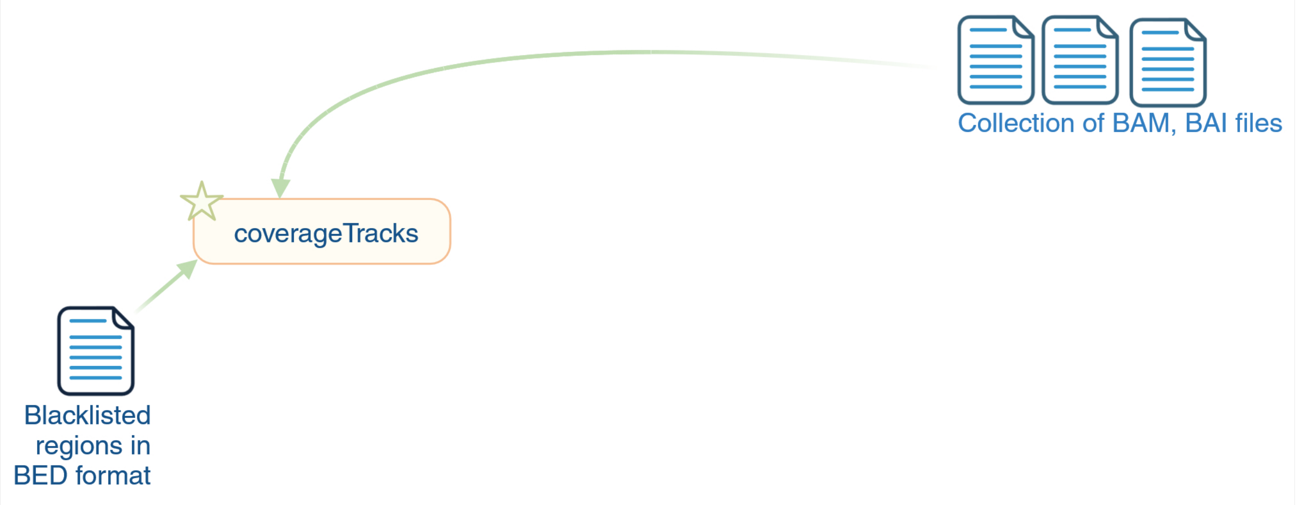

Figure 10.1: The workflow of the coverageTracks mode of the greenPipes.

10.1 Distribution of spike-in

In the following section we used a constant for normalizing coverage on basis of spike-in. To find this constant, we calculated the total number of the spike-in reads the past and ongoing projects (unpublished) of our lab. In total, 152 experiment and 76 controls were found. Of which, 94 samples were of greenCUT&RUN while 134 were of CUT&RUN. Seven researchers (or handlers) performed individual experiments on their protein of interest (either transcription factors or histone modifications or proteins which are part of large complex e.g. MLL1, TBP, TAF1 etc.).

We observed that controls consistently had a higher proportion of spike-in compared to experiments in both greenCUT&RUN and CUT&RUN. As a result, a warning system is implemented into greenPipes. If any experiment shows a higher number of spike-in compared to its control, greenPipes will issue a warning. CUT&RUN had higher number of spike-in compared to the greenCUT&RUN. The higher amount of spike-in in CUT&RUN experiments and experiments involving histone marks are due to higher initial amount of spike-in added. In the experiment and control of greenCUT&RUN, 4.38$\pm$3.65% and 4.85$\pm$4.97% of total reads were spike-in while of CUT&RUN 8.32$\pm$7.09% and 16.08$\pm$9.00% of total reads were spike-in, respectively. The percentage of spike-in is also different depending on the protein target. If target has a high number

of the peaks, the spike-in ratio is smaller. On an average, we expect ≈5% and ≈10% spike-in in greenCUT&RUN and CUT&RUN experiments. We usually see this percentage when the amount of spike-in added in solution is 20 pg per sample in greenCUT&RUN and 100 pg per sample in CUT&RUN. Based on this observation, we selected 0.05 and 0.10 as constants for spike-in normalization in greenCUT&RUN and CUT&RUN, respectively.

10.2 coverageTracks

Following peak calling and/or identification of differential peaks, the logical next step is to visualize these peaks and to generate figures of genomic tracks suitable for publication. Users can achieve this visualization through the Integrative Genomics Viewer (IGV) (https://software.broadinstitute.org/software/igv/download). However, to do so, they need coverage data for their samples. greenPipes utilizes the bamCoverage function of deepTools to generate coverage files in bigWig format (format details available at https://genome.ucsc.edu/FAQ/FAQformat.html). Note that the inputs for this function are bam files aligned with the reference genome of interest, not with spike-in. This mode depends on the successfull completion of alignment mode. Given that users may wish to visualize and to compare coverage of differential peaks among experiments in IGV, spike-in normalized coverage should be generated for this purpose.

There are two different ways to normalize coverage in greenPipes. For example, assume that the user performed N number of experiments and n^{th} experiment can be denoted as $expr_n$. For $expr_n$ experiment, assume that the total number of human (or organism of interest) and spike-in reads in experiment are $E_{expr_n;h}$ and $E_{expr_n;S}$. If coverage of $expr_n$ within a bin is $Coverage_{expr_n}$, then normalized coverage $normCoverage_{expr_n}$ can be either calculated as follows:

$$spike - inratio_{expr_n} = \left(\frac{E_{expr_n;S}}{E_{expr_n;h} + E_{expr_n;S}}\right)$$

$$normCoverage_{expr_n} = Coverage_{expr_n} \times \left[\frac{min_{n \in N}\left(spike - inratio_{expr_n}\right)}{spike - inratio_{expr_n}}\right]$$

. . . equation 4

Or, this can be calculated as follows:
For greenCUT&RUN:

$$normCoverage_{expr_n} = Coverage_{expr_n} \times \left[\frac{0.05}{spike - inratio_{expr_n}}\right]$$

. . . equation 5

For CUT&RUN:

$$normCoverage_{expr_n} = Coverage_{expr_n} \times \left[\frac{0.1}{spike - inratio_{expr_n}}\right]$$

. . . equation 6

Following normalization, upload this (*bw) file to IGV and inspect the background coverage. All samples related to the same type of experiment should ideally exhibit similar background coverage. When there is considerable variation in background coverage, this may indicate issues

with the spike-in normalization. For instance, assume that user has three samples for SampleA and three samples for H3K4me3, the background coverage (not peak) among the SampleA samples should appear roughly equal. Similarly, background coverage for H3K4me3 should also appear consistent. It is acceptable if the background coverage between SampleA and H3K4me3 experiments differs.

This mode has three options: `--covSpike (True,False)`, `--covSpike_NormalizationFormula (1,2)`, `--covOtherOptions` and `--covExprType`.

By utilizing `--covSpike_NormalizationFormula`, the user has the flexibility to select between equations 4 and 4/5. Specifically, with `--covSpike_NormalizationFormula 1`, the user opts for equation 4, while with `--covSpike_NormalizationFormula 2`, the user selects equation 5 for normalization. It is essential to note that when using equation 4, the user can only compare coverage within a given set of samples. If the user intends to compare new samples, they would need to generate a spike-in normalized coverage file once again. Therefore, our recommendation is to use equation 5.

With `--covExprType`, user can specify the experiment type. Use gCR for greenCUT&RUN and CR for CUT&RUN. greenPipes will choose equation 5 or 6 on basis of this. It is already mentioned in the above section "Distribution of spike-in" that we usually see 5% and 10% spike-in in greenCUT&RUN and CUT&RUN, respectively. This was the reason, we used 0.05 and 0.1 on the basis of its high likelihood. Less likely, but we expect that this number might change in users laboratory set up. Therefore, we recommend users to run few experiments and check the proportion of spike-in in their dataset, and then change this number in the equation.

For this, open covTrack.py file present in the source greenPipes folder in any text editors. The user might have already downloaded this folder using command `git clone https://github.com/snizam001/greenPipe.git` (see chapter 2). Search and edit following line, and then reinstall greenPipes using command `pip install ./`:

```
#-------------------------------------------------
# you can change the 0.05 and 0.1 number
# according to your laboratory set up
#-------------------------------------------------
elif covExprType == "gCR":
    SpikeVal = [ 0.05 / SpikeRatio for SpikeRatio in SpikeRatios]
elif covExprType == "CR":
    SpikeVal = [ 0.1 / SpikeRatio for SpikeRatio in SpikeRatios]
#-------------------------------------------------
#
#-------------------------------------------------
```

Using `--covSpike` option, the user can specify whether they want to stop spike-in normalization during preparation of the coverage file.

greenPipes uses all default options of bamCoverage https://deeptools.readthedocs.io/en/develop/content/tools/bamCoverage.html, but if user wants to make changes besides `--bl`, `--effectiveGenomeSize` and `-p` option of bamCoverage, the other options can be provided as comma-separated values in large bracket using option `--covOtherOptions`. This mode also needs `--effectiveGenomeSize` and `--blackListedRegions`.

An example command for paired-end reads can be as follows:

```
greenPipes \
--inputfile ./Samplesheet.txt \
--inputdir $(pwd)/Fastq \
--outputdir $(pwd) \
--modes coverageTracks \
--blackListedRegions ~/Database/hg38-blacklist.v2.bed \
--covSpike True \
--covSpike_NormalizationFormula 2 \
--covExprType gCR \
--covOtherOptions [-bs,30,--minMappingQuality,30]
```

A single directory bamcoverage will be generated in the output folder. For each sample *bw files will be generated. For the experiment *_expr.bw and for the control *_control.bw will be generated. When user direct greenPipes to use spike-in normalization, the name of bamcoverage output file will be *_expr.Scaledspike-in.bw. As user can note in the name of file, spike-in normalized coverage file will be generated only for experiments not for controls. These *bw files can be uploaded directly to IGV for Visualization.

```
output folder
|-- bamcoverage/
    |-- SampleA_control.bw
    |-- SampleA_expr.bw
    |-- SampleA_expr.Scaledspike-in.bw
    |-- Normalization32.txt

.. cut ..
```

Besides these, a summary file Normalization{random number}.txt will be generated. The format of this file is given in **Table 10.1**. The first column is name of the experiment. This matches with the fifth column of sample sheet. The second and third column contains the total number of reads of spike-in and human reads in experiment, respectively. The fourth column is equal to spike-in ratio$_{\mathrm{expr_n}}$ and fifth column is equal to $\left[\frac{min_{n\in N}\left(spike-inratio_{\mathrm{expr_n}}\right)}{spike-inratio_{\mathrm{expr_n}}}\right]$ or $\left[\frac{0.1\ or\ 0.05}{spike-inratio_{\mathrm{expr_n}}}\right]$. The user can use this file to track how normalization was performed within greenPipes.

Table 10.1: Format of Normalization{random number}.txt

Name	Spike	Experiment	Ratio of Spike	Normalization
SampleA	54134	991693	0.052	0.97
SampleB	59240	991277	0.056	0.89
..	..	..	..	..

Chapter 11

Footprinting

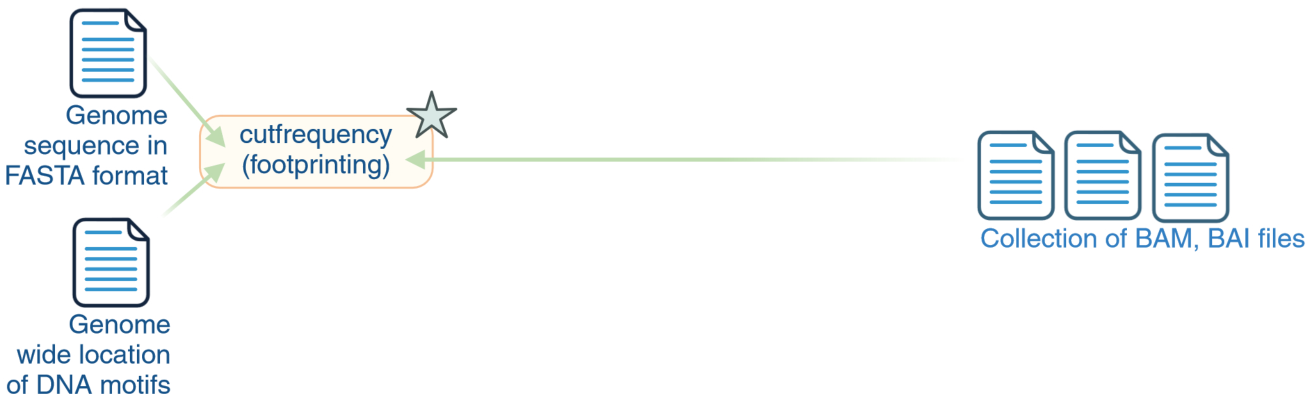

Figure 11.1: The workflow of the alignment and equalRead mode of the greenPipes.

11.1 cutfrequency

greenCUT&RUN and CUT&RUN provide footprinting information at base-pair resolution. These footprints are unique to the transcription factors as we have already observed in case of NFYA and JUN protein[1]. The footprint of NFYA is shown in **Figure 11.2**.

Several tools exist for calculating cut frequency around DNA motifs, but greenPipes stands out due to its unique approach to normalization and bias removal. Unlike other tools, greenPipes normalizes cut frequency against both control and spike-in. This comprehensive normalization accounts for preferential DNA cleavage at nucleotides, ensuring more accurate and unbiased results.

$$
\begin{aligned}
Cf_j &= 10^6 \times \frac{1}{N_{motifs}} \\
&\times \sum_{i\,=\,1}^{i\,=\,N_{motifs}} \left\{ \left(\frac{R_{expr_i}}{N_{expr}} \right) - \left(\frac{R_{ctrl_i}}{N_{ctrl}} \times \frac{E_s}{C_s} \right) \right\}
\end{aligned}
$$

[1] Sheikh Nizamuddin, Stefanie Koidl, Tanja Bhuiyan, Tamara V Werner, Martin L Biniossek, Alexandre MJJ Bonvin, Silke Lassmann, HT Marc Timmers (2021). "Integrating quantitative proteomics with accurate genome profiling of transcription factors by greenCUT&RUN." Nucleic acids research 49(9): e49-e49.

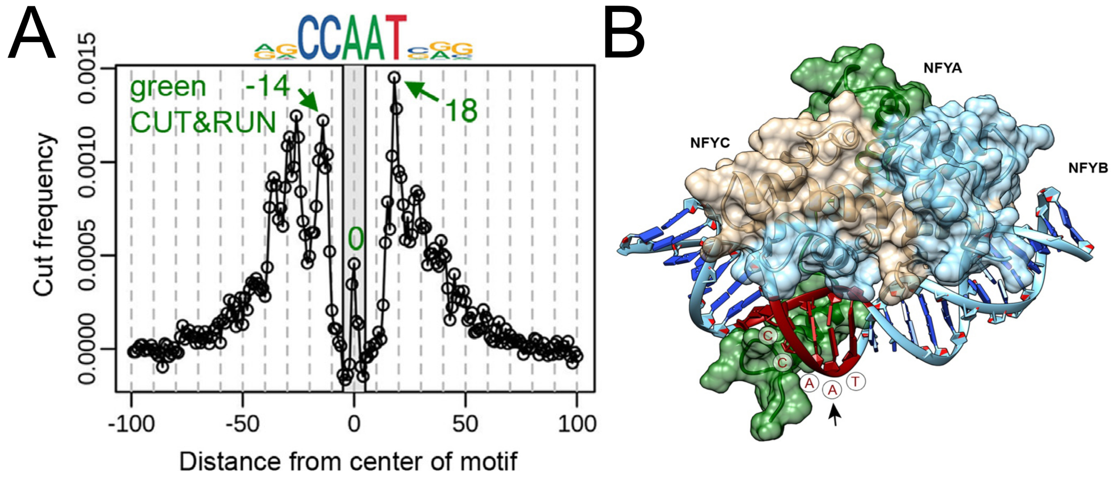

Figure 11.2: Panel A shows normalized cut frequency around NFYA DNA motif while panel B shows the crystallographic structure of NFY complex bound to DNA (PDB ID: 4AWL). Arrow points at 0th nucleotide. Observation in cutfrequency highly correlates with the crystallographical structure. This figure is reproduced from Nizamuddin, S. and coworker, NAR (2021)[1]

Where (i) N_{motifs} is total number of whole-genome motifs, (ii) N_{expr} and N_{ctrl} are total number of reads in experiment and control, (iii) R_{expr_i} and R_{ctrl_i} are number of read ends at j^{th} position from i^{th} motif and (iv) E_s and C_s are total number of spike-in reads in experiment and control, respectively.

This mode has five options: `--cMotif (True,False)`, `--cMotifFile`, `--cMaN`, `--cGVersion` and `--cCenter`.

This mode requires the genomic locations of motifs in the whole genome in HOMER BED format (note that this file format is slightly different than UCSC; check here: http://homer.ucsd.edu/homer/ngs/formats.html). If the user already has this file, they can provide it using the `--cMotifFile` option, specifying the full path of the file. When this file is not available, the user can instruct greenPipes to create this during calculation of cut frequency by using `--cMotif True`. During the preparation of the HOMER BED file and the calculation of cut frequency, users should provide the MA number of the JASPER motif (visit http://jaspar.genereg.net/ to find this number; for example, the MA number for TP53 is MA0106) and also provide the `--cGVersion`.

Options for `--cGVersion` is: human (hg18, hg19, hg38), mouse (mm8, mm9, mm10), rat (rn4, rn5, rn6), frog (xenTro2, xenTro3), zebrafish (danRer7), drosophila (dm3), *C. elegans* (ce6, ce10), *Saccharomyces cerevisiae* (sacCer2, sacCer3), *Schizosaccharomyces pombe* (ASM294v1), arabidopsis (tair10), and rice (msu6). The annotation libraries for one of the supported genomes has been already set-up during installation of greenPipes. When the user has their own reference genome, they can follow the tutorial at http://homer.ucsd.edu/homer/introduction/update.html to add the annotation library for their genome of interest in the greenpipes conda environment.

With the `--cCenter` option, the user can specify the center of motifs around which cut frequency will be calculated. For example, if the motif is NNNCCAATN and the user wants to center it at NNNCC[A]ATN, they should provide `--cCenter 6`.

An example command (when user do not have BED file for motifs) is as follows:

```
greenPipes \
--outputdir $(pwd) \
--modes cutfrequency \
```

```
--cMotif True \
--cMaN MA0060 \
--cGVersion hg38 \
--inputfile ./SampleInfo.txt \
--inputdir $(pwd)/Fastq \
--cCenter 8
```

The example command, assuming the user has a HOMER BED file for motifs, is as follows. In this command, the user should specify that the library type is paired-end, as cut frequency only works for paired-end libraries:

```
greenPipes \
--outputdir $(pwd) \
--modes cutfrequency \
--cMotif False \
--cMotifFile ./motifDatabase/MA0060.bed \
--cMaN  MA0060 \
--cGVersion hg38 \
--cCenter 8 \
--libraryType pair
```

A single directory named *cut_frequency* will be generated in the output folder. If the user has specified `--cMotif True`, then another folder named *motifDatabase* will also be generated. This folder will contain HOMER BED file. The format of this file is given in the **Table 11.1**. Additionally, this mode will generate *in files in the *Tagdirectories_qualities* folder, which will contain information about reads overlapping with the motifs.

Table 11.1: Format of cMotifFile.

Chr	start	end	name	score	strand
chr1	14421	14431	MA0060.3:::NFYA	8.021483	-
chr1	25657	25667	MA0060.3:::NFYA	8.187014	-
chr1	31182	31192	MA0060.3:::NFYA	7.484397	-
chr1	35315	35325	MA0060.3:::NFYA	8.487435	-
chr1	48478	48488	MA0060.3:::NFYA	10.503611	+
chr1	54586	54596	MA0060.3:::NFYA	7.921064	+
chr1	67142	67152	MA0060.3:::NFYA	7.046773	+
..	..	..	..	..	..

```
output folder
|-- motifDatabase/
    |-- MA0060.bed
.. cut ..

|-- cut_frequency/
    |-- SampleA.counts.txt
    |-- SampleA.cutfrequency.txt
```

```
    |-- SampleA.jpeg
    |-- SampleA.normalize.txt

.. cut ..
```

Four files will be generated for each samples. *counts.txt will contain count of the end of reads overlapping with distance from the center of motif. greenPipes calculates cut frequency from -100 to 100 base-pairs only. *normalize.txt will contain normalized cut frequency (Cf_j). *cutfrequency.txt will contain sum of the Cf_j per base pair. An example format of these files are given in Table 11.2, 11.3 and 11.4.

Table 11.2: Format of *count.txt.

Motifs location	Count of the ends of reads at each base pair location											
	-100	-99	-98	-97	-95	..	95	96	97	98	99	100
chr1:1000184-1000194<br>..	0<br>..	0<br>..	0<br>..	0<br>..	0<br>..	..<br>..	0<br>..	0<br>..	0<br>..	0<br>..	0<br>..	0<br>..

Table 11.3: Format of *normalize.txt.

Motifs location	Normalized count of the ends of reads at each base pair location											
	-100	-99	-98	-97	-95	..	95	96	97	98	99	100
chr1:1000184-1000194<br>..	0<br>..	0<br>..	0<br>..	0<br>..	0<br>..	..<br>..	1.3×10^{-7}<br>..	0<br>..	0<br>..	0<br>..	0<br>..	0<br>..

Table 11.4: Format of *cutfrequency.txt.

Distance from center of motif	sum of the Cf_j
-100	0.0004
-99	0.0003
-98	0.0002
-97	0.0002
-95	0.0003
..	..
95	0.0003
96	0.0003
97	0.0006
98	0.0002
99	0.0003
100	0.0003

Chapter 12

Heatmap

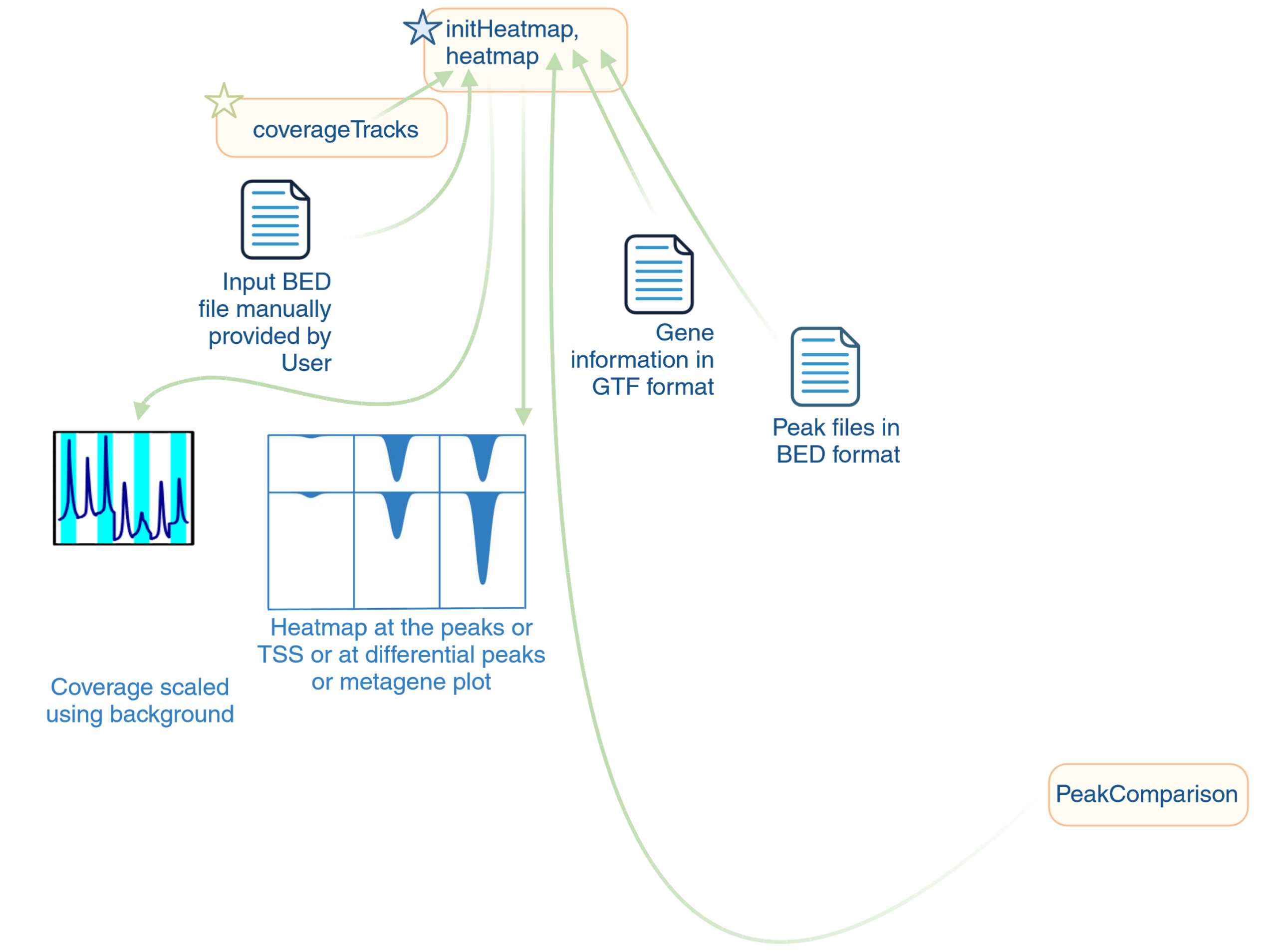

Figure 12.1: The workflow of the initHeatmap and heatmap mode of the greenPipes.

A heatmap is a way to visualize and compare the intensities of all peaks among different samples. greenPipes uses the computeMatrix and plotHeatmap function of deeptools and other internal functions in this mode. There are different ways to generate heatmap. Users can either use bamCompare files or bamCoverage files to generate heatmaps. The bamCompare files can be generated by comparing coverage in experiment *vs.* control in a spike-in normalization manner using initHeatmap mode. The bamCoverage files normalized or non-normalized by spike-in will be generated in `coverageTracks` mode.

12.1 initHeatmap

Using this mode, the user can generate bamCompare files. This uses the bamCompare function of deeptools (https://deeptools.readthedocs.io/en/develop/content/tools/bamCompare.html).

Options available for this mode are: `--initHeatmapOtherOptions` and `--hSpike (True,False)`.

With `--initHeatmapOtherOptions`, the user can change default parameters of bamCompare (deepTools) except `--bl`, `--effectiveGenomeSize` and `-p`. The user should provide various parameters as a comma-separated values in a large bracket. This mode also needs `--blackListedRegions`, so that falsly enriched regions can be removed from the files. It will also needs library type (use option `--libraryType`). If user want to switch off the spike-in normalization, they can do it by using `--hSpike False`. The example command is as follows:

```
greenPipes \
--outputdir $(pwd) \
--modes initHeatmap \
--inputfile ./SampleInfo.txt \
--inputdir $(pwd)/Fastq \
--libraryType pair \
--hSpike True \
--blackListedRegions ~/Database/hg38-blacklist.v2.bed \
--initHeatmapOtherOptions [-bs,20,--minMappingQuality,15]
```

greenPipes automatically normalized the coverage by spike-in using `--scaleFactors` option of bamCoverage tool. The format of argument for `--scaleFactors` is F_{expr}:$F_{control}$. This will cause the first BAM file to be multiplied by F_{expr}, while second BAM file multiplied by $F_{control}$. greenPipes always uses F_{expr} = 1. For $F_{control}$, it uses normalization factor equal to $1/\left\{\left(\frac{C_S}{C_S+C_h}\right) \times \left(\frac{E_S+E_h}{E_S}\right)\right\}$. Here, total number of human (or organism of interest) and spike-in reads in experiment are E_h and E_S while in control are C_h and C_S. A single directory bamcompare will be generated in output folder. For each samples *bw file will exist in this folder.

```
output folder
|-- bamcompare/
    |-- SampleA.bw
    |-- SampleB.bw

.. cut ..
```

12.2 heatmap

This is the main mode of this chapter. There are different ways to generate normalized heatmap. Most of them are supported here.

Options associated with this mode are: `--hMOpt`, `--hPOpt`, `--hCovComp (compare,coverage)`, `--hCovMethod (1,2)`, `--hInCounts`, `--hInFiles`, `--hInNames`, `--hRegionMode (tss,metagene,bed,peaks)`, `--hGtf`, `--hBed`, `--hPeakType`

(narrow-homer,broad-homer,all-homer,seacr.stringent,seacr.relaxed) and --hDiffPeaks (True,False). This mode also needs --blackListedRegions.

greenPipes uses default parameters of the computeMatrix (https://deeptools.readthedocs.io/en/develop/content/tools/computeMatrix.html) and plotHeatmap (https://deeptools.readthedocs.io/en/develop/content/tools/plotHeatmap.html). If user wants to change any parameter of computeMatrix beside --missingDataAsZero, -bl, --smartLabels, -p and --metagene, they can specify this using --hMOpt as comma-separated values in a large bracket ([ ]). Similary, if user wants to change parameters for plotHeatmap except --refPointLabel and --dpi, they can use option --hPOpt and specify arguments as comma-separated values in a large bracket ([ ]). By default, greenPipes uses --colorMap GnBu to generate attractive heatmap of publication quality.

With --hCovComp, the user can specify whether they want to use bamCompare or coverage file. When the user is using coverage files to generate the heatmap, there are two ways to use this and it can be specified by --hCovMethod.

When using --hCovMethod 1, the user can utilize a pre-prepared spike-in normalized bamcoverage file, created using the coverageTracks mode. Alternatively, with --hCovMethod 2, the greenPipes employs a coverage file without normalization as input for computeMatrix and performs normalization as described below. This option is provided for users who possess non-normalized coverage files and wish to utilize the heatmap mode of the greenPipes.

Let's assume that computeMatrix generates a matrix for a sample containing E_S reads of spike-in in an experiment using a non-normalized coverage file. This matrix is generated for L locations, which can represent TSS, peaks, etc. As outlined in the deeptools manual, computeMatrix divides the region into n bins from the center of the location and calculates the number of reads overlapping within each bin at every base. Based on this, it computes the sum of nucleotides present within the bin if --averageTypeBins sum is specified as a computeMatrix option. The pipeline utilizes the same option. Suppose the sum of nucleotides in a bin is R, and $_{\text{nr}}R$ is the normalized sum; the normalization process is as follows:

$$\begin{pmatrix} _{\text{nr}}R_{1,1} & \cdots & _{\text{nr}}R_{n,1} \\ _{\text{nr}}R_{1,2} & \cdots & _{\text{nr}}R_{n,2} \\ _{\text{nr}}R_{1,3} & \cdots & _{\text{nr}}R_{n,3} \\ \vdots & \ddots & \vdots \\ _{\text{nr}}R_{1,L} & \cdots & _{\text{nr}}R_{n,L} \end{pmatrix} = \begin{pmatrix} R_{1,1} & \cdots & R_{n,1} \\ R_{1,2} & \cdots & R_{n,2} \\ R_{1,3} & \cdots & R_{n,3} \\ \vdots & \ddots & \vdots \\ R_{1,L} & \cdots & R_{n,L} \end{pmatrix} / \frac{E_S}{10000}$$

Note that if users are utilizing bamCompare files, they cannot directly compare peak intensity across experiments but can compare the presence or absence of peaks. However, if users are working with normalized coverage files, they can compare peak intensity. This holds true even when using a non-normalized coverage file, provided it is normalized using --hCovMethod 2. It is important to highlight that comparing peak intensity for a new sample is not feasible when using coverage files normalized by equation 4.

greenPipes automatically searches coverage or compare files in the output folder. If the user prefers to provide their own input file, they can provide it by using option -hInFiles as comma-separated values (csv). Then, they need to specify the name of experiments also using --hInNames as csv.

When the user provides their own coverage files using --hInFiles and wish to normalize them using custom normalization counts, they can specify these counts for each sample using --hInCounts in a comma-separated value format. Otherwise, if --hInFiles is provided without --hInCounts, greenPipes will by default use the total number of the reads sequenced in each experiment for the normalization.

By default, greenPipes searches narrow peak files generated by HOMER in output directory and uses it to generate heatmap. The user can change this behaviour. If user wants to generate heatmap on giving his own peak location file in BED format or want to generated heatmap on TSS (transcription start sites) or want to generate metagene plot, they can use `--hRegionMode bed` or `--hRegionMode tss` or `--hRegionMode metagene`, respectively. With `--hBed`, user can provide path of the peak location file. If TSS (transcription start site) or metagene specified, then the user needs to provide the GTF file also by using `--hGtf` option. Please give full path of files. For human genome version hg38, download gtf file from https://osf.io/ruhj9/. Format of GTF file is explained at https://www.ensembl.org/info/website/upload/gff.html.

If user wants to use other peak files (*narrow-homer, *broad-homer, *all-homer, *seacr.stringent or *seacr.relaxed) to generate heatmaps, they can specify it by using `--hPeakType`. If user wants that greenPipes generates heatmaps for differential peaks also, then they should specify `--hDiffPeaks True`. It will automatically search these files otherwise users need to provide it as follows: `--hRegionMode bed` and `--hBed file1.bed,file2.bed`. The example command is as follows:

```
bedfiles="Peaks/MEN1.h3k4me3JunDMLL1.bed,Peaks/\
MEN1.JunDMLL1.bed,Peaks/MEN1.h3k4me3JunD.bed,Peaks/\
MEN1.onlyJunD.bed,Peaks/MEN1.onlyMLL1.bed,Peaks/\
MEN1.onlyh3k4me3.bed,Peaks/MEN1.h3k4me3MLL1.bed,Peaks/\
MEN1.unknown.bed"

inFiles="./bamcompare/MEN1.bw,./bamcompare/MLL1.bw,./\
bamcompare/JunD.bw,./bamcompare/\
ENCFF063XTI.rep1.filtered.bamcompare.bw,./bamcompare/\
ENCFF241VRU.rep1.filtered.bamcompare.bw,./bamcompare/\
ENCFF617YCQ.rep1.filtered.bamcompare.bw,./bamcompare/\
ENCFF113QJM.rep1.filtered.bamcompare.bw,./bamcompare/\
ENCFF725TAB.rep1.filtered.bamcompare.bw,./bamcompare/\
PolII-RPB1.bw"

name="MEN1,MLL1,JunD,H3k4me3,H3k4me2,H3k4me1,H3k27ac,\
H3k9ac,PolII(RPB1)"

greenPipes \
--outputdir $(pwd) \
--modes heatmap \
--inputfile ./SampleInfo.txt \
--inputdir $(pwd)/Fastq \
--libraryType pair \
--blackListedRegions ~/Database/hg38-blacklist.v2.bed  \
--hRegionMode bed \
--hBed $bedfiles \
--hInFiles $inFiles \
--hInNames $name \
--hPOpt [--zMin,-2,--zMax,5]
```

The above command will generate a image like illustrated in the **Figure 12.3**.
Another example is:

```
greenPipes \
--outputdir $(pwd) \
--modes heatmap \
--inputfile ./SampleInfo.txt \
--inputdir $(pwd)/Fastq \
--libraryType pair \
--blackListedRegions ~/Database/hg38-blacklist.v2.bed \
--hCovComp coverage \
--hCovMethod 2 \
--hRegionMode peaks \
--hDiffPeaks False
```

In the output folder, a dedicated directory named *Heatmap* will be created. Within this folder, six files will be generated for each sample. The *normalized files will contain information post-normalization. If the input file itself is a normalized file, then all files will contain normalized information. All *gz files represent the matrix of coverage, except for *curve.gz files. The *curve files contain profile information, differing from deeptools' plotProfile in two ways: (1) each sample is plotted separately in different panels, and (2) backgrounds are set to zero. An examples is illustrated in **Figure 12.2**. To set the background to zero, the matrix is scaled as follows:

$$\begin{pmatrix} \text{nr}R_{1,1} & \cdots & \text{nr}R_{n,1} \\ \text{nr}R_{1,2} & \cdots & \text{nr}R_{n,2} \\ \text{nr}R_{1,3} & \cdots & \text{nr}R_{n,3} \\ \vdots & \ddots & \vdots \\ \text{nr}R_{1,L} & \cdots & \text{nr}R_{n,L} \end{pmatrix}$$

$$\Downarrow column\ means$$

$$\begin{pmatrix} \frac{\sum_{i=1}^{L} \text{nr}R_{1,i}}{L} & \cdots & \frac{\sum_{i=1}^{L} \text{nr}R_{n,i}}{L} \end{pmatrix}$$

$$\Downarrow scaling$$

$$\begin{pmatrix} \frac{\sum_{i=1}^{L} \text{nr}R_{1,i}}{L} & \cdots & \frac{\sum_{i=1}^{L} \text{nr}R_{n,i}}{L} \end{pmatrix} - min \begin{pmatrix} \frac{\sum_{i=1}^{L} \text{nr}R_{1,i}}{L} & \cdots & \frac{\sum_{i=1}^{L} \text{nr}R_{n,i}}{L} \end{pmatrix}$$

Scaling is beneficial for comparison purposes. In certain experiments, such as those involving the transcription factor TP53 and H3K27me3, users may observe elevated background levels compared to JUN/FOS and H3K4me3, respectively. In such cases, opting for scaling before comparing coverage among histone marks or transcription factors can be advantageous. This approach ensures that users are comparing the true coverage of the peaks. It is important to note that if the user observes higher background levels in some samples compared to others for the same protein of interest, this could indicate an issue with spike-in normalization. This may be attributed to the addition of an insufficient amount of spike-in in the experiment, leading to a high standard deviation, or the experiment itself may have encountered issues (which can be verified by checking the coverage in IGV).

```
output folder
|-- HeatMaps/
    |-- heatmap-coverage-peaks.gz
    |-- heatmap-coverage-peaks.jpeg
    |-- heatmap-coverage-peaks-normalized-curve.gz
```

```
|-- heatmap-coverage-peaks-normalized-curve.jpeg
|-- heatmap-coverage-peaks-normalized.gz
|-- heatmap-coverage-peaks-normalized.jpeg

.. cut ..
```

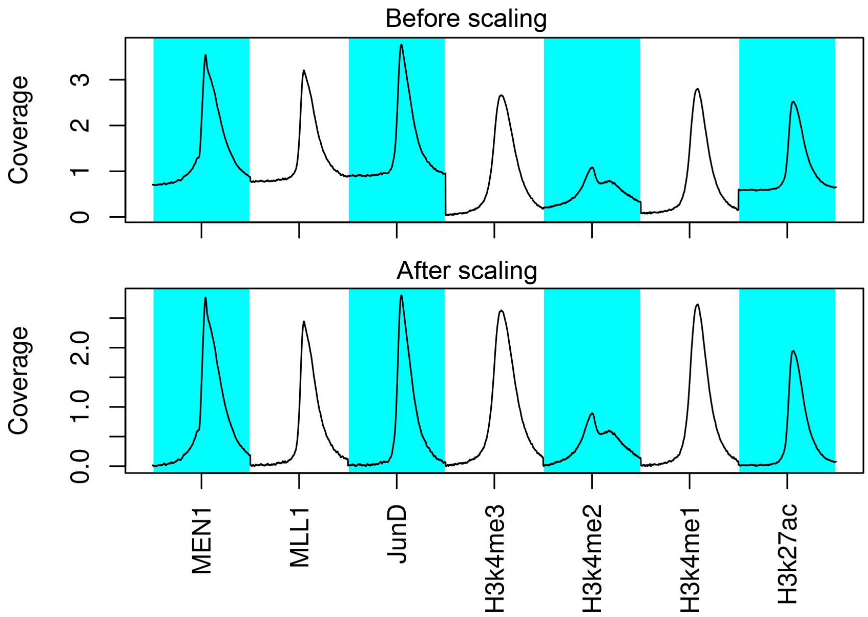

Figure 12.2: Coverage of heatmaps before and after scaling. The background coverage was different among samples. It is because the first three samples are coverage extracted from the coverage file while other last found samples are bamCompare file, which means background reads are already extracted out from the experiments.

[1] Koen Dreijerink, Ezgi Ozyerli-Goknar, Stefanie Koidl, Ewoud J van der Lelij, Priscilla van den Heuvel, Jeffrey J Kooijman, Martin L Biniossek, Kees W Rodenburg, Sheikh Nizamuddin, HT Marc Timmers. "Multi omics analyses of MEN1 missense mutations identify disruption of menin-MLL and menin JunD interactions as critical requirements for molecular pathogenicity". Epigenetics & Chromatin 15, 29 (2022).

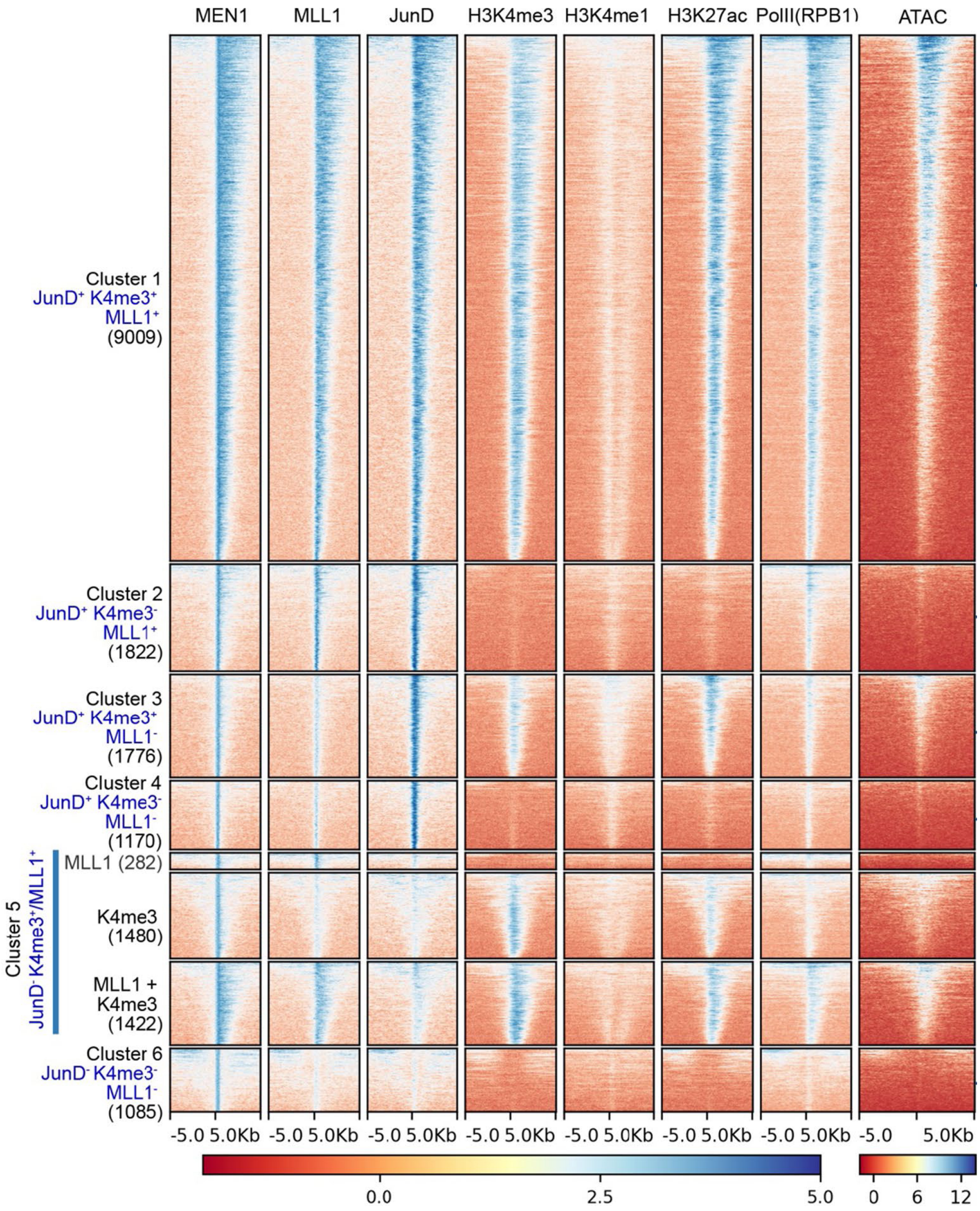

Figure 12.3: An example of heatmap that can be generated using greenPipes. The name of the panels on the left side and ATAC panel were added in Photoshop. Source of figure is Dreijerink et al (2022)[1].

Chapter 13

Integration and comparison with other -omics datasets

13.1 Integration with IP-based mass spectrometry

13.1.1 piggyBack

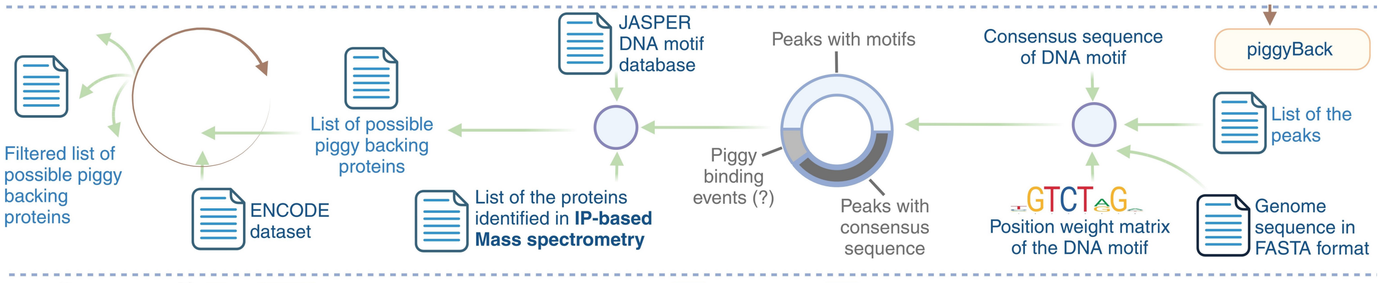

Figure 13.1: The workflow of the piggyBack mode of the greenPipes.

Numerous laboratories engaged in studies of DNA or chromatin-binding protein complexes often conduct immunoprecipitation (IP)-based mass spectrometry and integrate the results with genomic localization techniques, such as greenCUT&RUN, CUT&RUN, ChIPseq, etc. This mode is developed to integrate proteomic with genomic datasets. On a related note, we anticipate that combining GFP-based IP-MS with greenCUT&RUN will result in decreased heterogeneity, as both methods employ the same nanobody recognizing GFP. The workflow of this mode is illustrated in **Figure 13.1**.

We observed the piggyback binding event for the NFY-complex. A considerable proportion of NFYA peaks lacked the CAAT-box but contained SP1 motifs. In IP-MS, we observed NFYA interacting with SP1, and in other ENCODE datasets, we noted the binding of both SP1 and NFYA at these sites. This strongly indicates a piggyback binding event of the protein (**Figure 13.2**). The next logical step is to delete SP1 from the cells and perform an NFYA greenCUT&RUN experiment.

In brief, the workflow is outlined as follows. From the peak list generated in the analysis of greenCUT&RUN and CUT&RUN data, greenPipes initially distinguishes peaks that lack both DNA motifs and the DNA binding sequence of the protein of interest. Please note that greenPipes allows users to include ambiguous nucleotide codes when providing the DNA binding sequence of the motif. Within these motif- or sequence-lacking peaks, greenPipes proceeds to identify significant "known" DNA motifs. Subsequently, it identifies proteins in the IP-based

mass spectrometry dataset that share the same "known" DNA motifs observed suggesting potential piggybacking proteins. To address ambiguity in protein names, the pipeline converts IDs to Entrez IDs. The next step involves searching for peak lists of piggybacking proteins in the ENCODE datasets and reporting any overlap with motif- or sequence-lacking peaks. The expectation is that true-positive piggybacking proteins will exhibit more overlap. Given the availability of numerous tools for analyzing proteomics data, such as MAXQUANT (https://maxquant.net/maxquant/) and PERSEUS (https://maxquant.net/perseus/), and the fact that these tools have already undergone benchmarking[3], greenPipes focuses on integration rather than conducting proteomics data analysis from scratch. It is important to be aware that this mode comes with limitations. Specifically, it can only predict piggybacking proteins that bind to DNA (not on histone modifications) and have a known position weight matrix (PWM) for their DNA motifs.

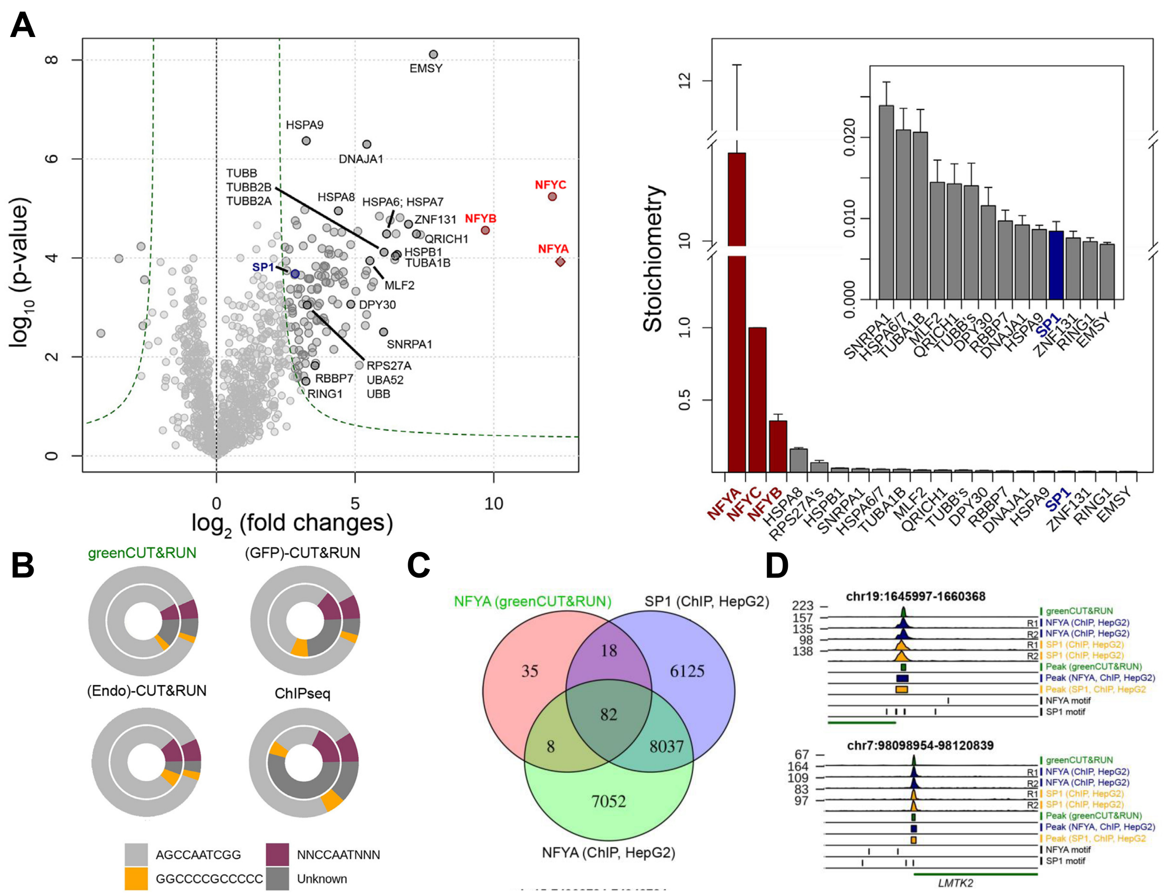

Figure 13.2: Integration of greenCUT&RUN with proteomics. Panel A shows the interacting protein of NFYA (on the left side). On the right side stochiometry of the interacting proteins are shown in form of bar graph. Note that SP1 is and interacting partner of the NFYA. Both Volcano and barplot is generated using IP-MS dataset. Panel B shows the distribution of motifs within the peaks. Peaks with SP1 motifs without CAAT-box motif or sequence is shown in orange color. Panel C shows overlap of the peaks of SP1 with NFYA and panel D shows two example tracks. SP1 data were obtained from the ENCODE repository. The tracks indicate that NFYA motif is not present, but SP1 DNA motif is present at these binding sites. Note that both NFYA and SP1 are binding to DNA. This suggests that SP1 is piggy backing NFYA at selected genomic locations. Source to this image is Nizamuddin, S and coworkers (2019) [1]

Options related to this mode include `--lProt`, `--mPwm`, `--sProt`, `--mPeak`, `--distPiggy`, `--Species (human, mouse, rat, fruitfly, nematode, zebrafish, thale-cress, frog, pig)`, `--sFasta`, `--gVer`, `--mPvalue`, `--sDist`, `--mPrefix`, `--dPeakfiles`, and `--dNames`.

For `--lProt`, provide the full path of the file containing names of proteins per line. These are proteins significantly interacting with your protein of interest in the mass spectrometry experiment. The user can obtain this list based on the FDR value in the PERSEUS tool. This file should not contain any special characters such as ; or - or :. With `--mPwm` and `--sProt`, provide the MA number and DNA-binding sequence of your protein of interest so that peaks containing motifs or sequences will be filtered. For example, for NFYA, the user can provide MA0060 and CCAAT. The user can also provide the IUPAC code of nucleotides in the sequence (e.g., for AP1, user can provide TGANTCA). Visit http://jaspar.genereg.net/ to find the MA number. With `--mPeak`, provide the path of the peak file in BED format. It is optional; if not given, the tool will automatically search for the peak file based on `--outputdir` and `--inputfile` options. Please give the full path of peaks as comma-separated values (e.g., /home/xyz/exp1.Clean.bed, /home/xyz/exp2.Clean.bed). If the user provides peak file manually, they should also provide the output prefix using `--mPrefix`. With `--mDist` and `--sDist`, provide the distance in base pairs. greenPipes will search the PWM and DNA sequence of motifs from this distance from the center of peaks, respectively. The default for `--mDist` is 400 base pairs (200 base pairs left and 200 base pairs right from the center of the peak). For `--sDist`, the default value is 400 base pairs and this is used to search the DNA sequence of the motif of protein of interest. Peaks without motifs and sequences will be filtered for further analysis. For piggybacking events, provide the distance with `--distPiggy`. It is possible that piggybacking events are binding farther from the center of peaks. The default value is 600 base pairs, and within this distance, piggyback binding will be searched. Specify the species using `--Species`. This is necessary to generate random background and find the p-value of finding PWM of motifs within the peaks. Provide the fasta file of the genome with `--sFasta`. The pipeline will use this file to find motif sequences. With `--gVer`, provide the version of your genome. This is needed for HOMER, and the default is hg38. greenPipes aims to find true positive PWM of motifs within the peaks and uses cutoff p-value of 0.05. If user wants to change it, they can use `--mPvalue` option of greenPipes. An example command is as follows:

```
greenPipes \
--outputdir $(pwd) \
--modes piggyBack \
--outputdir $(pwd) \
--lProt $(pwd)/piggyBack-lProtein.txt \
--mPeak $(pwd)/Peaks/SampleA_narrow-homer.Clean.bed \
--mPrefix SampleA \
--mPwm MA0060 \
--sProt CCAAT \
--Species human \
```

[1]Sheikh Nizamuddin, Stefanie Koidl, Tanja Bhuiyan, Tamara V Werner, Martin L Biniossek, Alexandre MJJ Bonvin, Silke Lassmann, HT Marc Timmers (2021). “Integrating quantitative proteomics with accurate genome profiling of transcription factors by greenCUT&RUN.” Nucleic acids research 49(9): e49-e49.

[2]Zhu J, Sammons MA, Donahue G, Dou Z, Vedadi M, Getlik M, Barsyte-Lovejoy D, Al-awar R, Katona BW, Shilatifard A, Huang J, Hua X, Arrowsmith CH, Berger SL. Gain-of-function p53 mutants co-opt chromatin pathways to drive cancer growth. 525(7568):206-11. Nature. 2015.

[3]Miao-Hsia Lin and others, Benchmarking differential expression, imputation and quantification methods for proteomics data, Briefings in Bioinformatics, 23 (3), 2022, bbac138.

```
--sFasta ~/Database/GRCh38.p13.fa \
--gVer hg38 \
--inputfile $(pwd)/SampleInfo.txt \
--inputdir $(pwd)/Fastq
```

Certain functions, such as the conversion of symbols to Entrez IDs and the download of ENCODE data, requires connection to the internet. The following error indicates that the computer is not currently connected to the internet.

```
.. cut ..
File "/home/sheikh/anaconda3/ \
lib/python3.9/site-packages/\
greenPipe/massSpectro.py", line 106, in massHugo2Entrez
mgout=mg.query(d.iloc[j,0],

.. cut ..
```

greenPipes will provide a summary during execution. For instance, in the terminal, the user will observe the following line when initiating the example dataset run. This line indicates that the total number of peaks provided to the pipeline was 5880, out of which 2143 had either a motif or sequence, and 195 remained unexplained representing potential sites for piggyback binding. This information can be utilized to generate figures, similar to **Figure 13.2 B**.

```
.. cut ..
Total number of the peaks in the *.Clean.bed is :5880

Total number of the peaks after filtering peaks containing \
pwmProteinOfInterest motifs :2143

Total number of the peaks after filtering peaks containing \
pwmProteinOfInterest motifs + sequenceProteinOfInterest :195

.. cut ..
```

A dedicated directory named "MassSpectrometry" will be created in the output folder. The file *.motifs_pwm.txt will contain list of motifs enriched in all given peaks. *motifs_pwm.txt will contain a list of the peaks with motifs of your protein of interest. *.peaks_WithoutPwmSeq.txt is the list of peaks without motifs and sequence of your protein of interest. The file *.peaks_WithoutPwmSeq/knownResults.txt will contain motifs called in those peaks which were neither having motifs nor sequence of protein of interest. The file *.WithoutmPwmWithoutSequence.txt is similar to *.peaks_WithoutPwmSeq/knownResults.txt, but having a p-value of motifs < (value specified by `--mPvalue`). Transcription factors in file *.WithoutmPwmWithoutSequence.txt will be checked in the IP-MS protein list (provided by `--lProt`). For each transcription factor matched in IP-MS, ENCODE datasets will be downloaded. Files with names like "ENCFF*bed" are obtained from the ENCODE. Details about the ENCODE datasets can be obtained at https://www.encodeproject.org/ using accession ID (ENCFF*). Besides this, the user can also obtain information of these accession IDs in file

*.peaks_WithoutPwmSeq_{*Entrez ID*}_SelectedDataSetsEncode.txt, were "Entrez ID" is for the transcription factor which is enriched in peaks without motif and sequence of your protein of interest. The file *.peaks_WithoutPwmSeqOnly{*symbol of gene*}.txt will contain list of peaks where transcription factor or putative piggy back's motifs will be present. After downloading ENCODE data, peaks present in ENCODE will be overlapped with peaks without motifs and sequence of your protein of your interest. VennDiagrams/*txt will contain information of overlapping peaks while VennDiagrams/*pdf will contain the Venn diagram of overlap.

```
output folder
|-- MassSpectrometry/
    |-- ENCFF038AVV.bed
    |-- ENCFF171NEU.bed
    |-- ENCFF284JVS.bed
    |-- ENCFF553GPK.bed
    |-- ENCFF600MKH.bed
    |-- ENCFF806TIM.bed
    |-- SampleA.motifs_pwm.txt
    |-- SampleA.peaks_WithoutPwmSeq
        |-- knownResults
            |-- known10.logo.svg

            .. cut ..

            |-- knownResults.html
            |-- knownResults.txt
            |-- motifFindingParameters.txt
            |-- seq.autonorm.tsv
    |-- SampleA.peaks_WithoutPwmSeq_6667_SelectedDataSetsEncode.txt
    |-- SampleA.peaks_WithoutPwmSeqOnlySP1.txt
    |-- SampleA.peaks_WithoutPwmSeq.txt
    |-- SampleA.totalPeaks.txt
    |-- SampleA.WithoutmPwmWithoutSequence.txt
    |-- temporary.pwm
    |-- VennDiagrams
        |-- SampleAvsSP1-GM12878ENCFF038AVV.Comparison.txt
        |-- SampleAvsSP1-GM12878ENCFF038AVV.Comparison_venn.pdf
        |-- SampleAvsSP1-H1ENCFF284JVS.Comparison.txt
        |-- SampleAvsSP1-H1ENCFF284JVS.Comparison_venn.pdf
        |-- SampleAvsSP1-HepG2ENCFF333SWC.Comparison.txt
        |-- SampleAvsSP1-HepG2ENCFF333SWC.Comparison_venn.pdf

        .. cut ..
.. cut ..
```

13.1.2 doughnut

If user only want to check the distribution of motifs in given peaks like **Figure 13.2.B**, then this mode can be used. Options associated with this mode include: `--mPwm`, `--sProt`, `--mDist`,

`--distPiggy`, `--Species`, `--sFasta`, `--gVer`, `--sDist`, `--dPeakfiles` and `--dNames`. Everything is similar to the mode piggyBack except `--dPeakfiles` and `--dNames`. Through `--dPeakfiles` provide peak file of your protein of interest in BED file format and `--dNames` provide prefix of output. The user can provide peak for more that one protein of interest as comma-separated values. An example command is as follows:

```
greenPipes \
--outputdir $(pwd) \
--modes doughnut \
--dPeakfiles input.bed \
--dNames SampleA \
--mPwm MA0060 \
--sProt CCAAT \
--Species human \
--sFasta ~/Database/GRCh38.p13.fa \
--gVer hg38
```

A single directory doughnut will be generated in output folder. Output files are similar to the piggyBack mode. We recommend to use piggyBack mode as that is more robust.

13.2 Comparison with other -omics technology

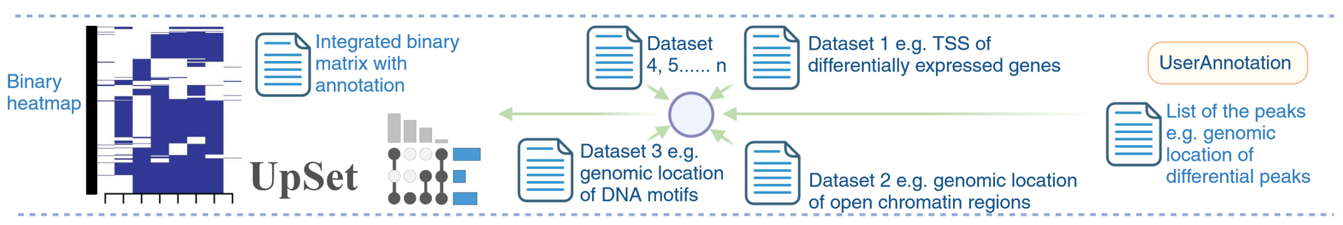

Figure 13.3: The workflow for the comparison of the other -omics datasets with CUT&RUN and greenCUT&RUN datasets in greenPipes pipeline.

13.2.1 Cis-elements/factors: Comparison with ATACseq and other omics technologies

To compare cis-acting elements such as open chromatin regions (ATAC-seq peaks), other histone modification peaks, transcription factor binding sites, DNA motifs, hyper/hypo-methylated regions, etc., users can utilize the UserAnnotation mode. This mode allows for the comparison of differential open chromatin regions, active/inactive histone markers, etc., with differential peaks identified in greenCUTRUN and CUTRUN experiments.

13.2.2 Trans-elements/factors: Comparison with transcriptomics

If user wishes to compare information about up/down-regulated genes with the intensity of up/down-regulated peaks on cis-acting elements, they can utilize the same UserAnnotation

mode. For the comparison of trans-acting elements, check whether enhancer-gene interaction datasets are available. A comprehensive interaction dataset for human cells is uploaded at https://osf.io/ruhj9 (Enhancer-gene-interaction-hg38.tar.gz). If user has Hi-C data, they can prepare a BED file of interactions and can use the UserAnnotation mode for comparison.

If enhancer-gene interaction data is not available, it becomes challenging. One option is to predict enhancer-gene interactions using the "IM-PET" algorithm, based on Random Forest and developed by the laboratory of Kai Tan (http://tanlab4generegulation.org). However, please note that this method is only applicable to the human genome version hg19. According to an email exchange with the developer of IM-PET, IM-PET for the mouse genome version mm9 are also available with developers. Users can contact the authors directly for information about the mouse genome. After generating enhancer-gene interactions, users can employ the UserAnnotation mode.

Chapter 14

Appendix

```
greenPipes --help
usage:

greenPipes pipeline (version 3.0): April, 2023

greenPipes [required arguments] [common arguments] [optional arguments]
greenPipes --help

options:
-h, --help            show this help message and
                        exit
--effectiveGenomeSize EFFECTIVEGENOMESIZE
                        callPeaks, idr,
                        coverageTracks, initHeatmap
                        mode: effective genome
                        size. human (2913022398:
                        hg38, 2864785220: hg19),
                        mouse (2652783500: GRCm38,
                        2620345972: GRCm37) and
                        fruitfly (142573017:dm6,
                        162367812: dm3). (default is
                        2913022398 which is
                        equivalent for the human
                        genome)
--refgenome REFGENOME
                        alignment mode:
                        bowtie2 index file of
                        reference genome. For
                        example: if bowtie2 indexes
                        is present in folder
                        /home/databases/genomes and
                        name of indexes are:
                        GRCh38.p13.1.bt2,
                        GRCh38.p13.2.bt2 etc; then
                        give path /home/databases/gen
```

```
                        omes/GRCh38.p13
  --spike-in spike-in   alignment mode:
                        bowtie2 index file of
                        spike-in (Drosophilla). Give
                        path of index as suggested
                        for --referenceGenome
  --alignParam ALIGNPARAM
                        alignment mode:
                        The alignment parameters
                        of the bowtie2/bwa. The
                        default parameter of bowtie2
                        in this pipeline is:
                        --dovetail --local --very-
                        sensitive-local --no-unal
                        --no-mixed --no-discordant -I
                        10 -X 700 (based on Meers et
                        al. (2019)). For single-end
                        default parameters of the
                        program of bwa was used.If
                        you want to change it, give
                        parameter as comma separated
                        values in large bracket e.g.
                        for bowtie2 [--no-unal,--no-
                        mixed,--no-
                        discordant,-I,0,-X,1500] and
                        for bwa see the manual of bwa
                        (for mem). If --gpu is True,
                        then see the options of the
                        nvBowtie: https://nvlabs.gith
                        ub.io/nvbio/nvbowtie_page.htm
                        l
  --gpu {True,False}  If you have good source of
                        the GPU. Use this option to
                        activate. Wherever neccessary
                        tool automatically recognize
                        and use the GPU source.
  --SelectReads SELECTREADS
                        equalRead mode:
                        How many reads should be
                        use randomly? If not given
                        minimum number of read will
                        be choosed using all
                        experimental files
  --spikeNormPeak {True,False}
                        initpeakcalling
                        mode: Should include
                        spike-in normalizationin peak
                        calling ? (default is True)
  --reverseName_equalRead {True,False}
```

```
                        equalRead mode:
                        If the bamfile folder
                        already contains
                        *.original.bam files and you
                        are selecting random reads,
                        then mode equalRead will
                        denote error. Therefore
                        either manually rename
                        *.original.bam to *.bam or
                        use this function to
                        automatically rename files.
--blackListedRegions BLACKLISTEDREGIONS
                        qcExperiment,
                        qcBamfiles, callPeaks, idr,
                        coverageTracks, initHeatmap,
                        heatmap mode: regions
                        which are black listed by
                        ENCODE in bed format.
--pMethod {homer,seacr}
                        callPeaks mode:
                        peak calling algorithm.
                        Default is homer
--pStyle narrow,broad,both
                        callPeaks mode:
                        style of the peaks
                        (narrow/broad/both). Default
                        is the narrow. For each
                        samples users can provide
                        comma separated stylese.g.
                        both,narrow,broad
--pFdrHomer PFDRHOMER
                        callPeaks mode:
                        FDR/poisson in the homer
                        peak calling. Default 0.001
--pPvalueHomer PPVALUEHOMER
                        callPeaks mode:
                        p-value in the homer peak
                        calling. Default is 0.0001
--pFcHomer PFCHOMER   callPeaks mode:
                        Fold changes in the homer
                        peak calling. Default is 4.0
--pDistHomer {fdr,poisson}
                        callPeaks mode:
                        Distribution (FDR or
                        poisson)
--pControl {True,False}
                        callPeaks mode:
                        Include control in the
                        homer peak calling? Default
```

```
                               is True
  --pSpike {True,False}
                               callPeaks mode:
                               Normalize reads by spike-
                               in in the homer peak calling.
                               Default is True
  --pSeacrMode {stringent,relaxed}
                               callPeaks mode:
                               Mode when calling peaks
                               using SEACR. Default is
                               strigent
  --pSeacrThreshold PSEACRTHRESHOLD
                               callPeaks mode:
                               Threshold value if
                               control is not used in peak
                               calling using SEACR. Default
                               is 0.01
  --pOpts POPTS              callPeaks mode:
                               Pipeline uses all default
                               parameters. If you want to
                               change something, in the
                               peakcalling from homer and
                               macs2 you can give here as
                               comma-separated values in
                               bracket.
  --genomeFile GENOMEFILE
                               callPeaks and
                               PeakComparison mode: With
                               SEACR mode --genomeFile is
                               require which contains the
                               length of each chromosome.
                               These files can be downloaded
                               from https://genome.ucsc.edu/
                               goldenpath/help/hg19.chrom.si
                               zes.Preferebly generate your
                               own genome file from fasta
                               (used in the alignment) by
                               using samtools faidx
                               genome.fa && cut -f1,2
                               genome.fa.fai >
                               genomeFile.txt. By default
                               program uses hg38 genome file
                               (hg38.genome)
  --idrExprs IDREXPRS        idr mode: List
                               of the tagDirectories of the
                               experiments (IDR mode). If
                               --idrMethod is homer then,
                               give as follows: /home/dir1/e
                               xp1_rep1,/home/dir1/exp1_rep2
```

```
                        ;/home/dir1/exp2_rep1,/home/d
                        ir1/exp2_rep2,/home/dir1/exp2
                        _rep3. Give full path. If
                        --idrMethod is macs2 then
                        give list of the bamfiles
--idrCtrl IDRCTRL     idr mode: List
                        of the tagDirectories of the
                        control (IDR mode). If
                        --idrMethod is homer then,
                        give as follows: /home/dir1/c
                        trl1_rep1,/home/dir1/ctrl1_re
                        p2;/home/dir1/ctrl2_rep1,/hom
                        e/dir1/ctrl2_rep2,/home/dir1/
                        ctrl2_rep3. Give full path.
                        If --idrMethod is macs2 then
                        give list of the bamfiles
--idrName IDRNAME     idr mode: List
                        of the Name of the experiment
                        (IDR mode). Give as follows:
                        expr1;expr2
--idrExprSpike IDREXPRSPIKE
                        idr mode: List
                        of the experiment bamfiles of
                        the spike-in, if --idrSpike is
                        True. Format is same. Give
                        full path
--idrCtrlSpike IDRCTRLSPIKE
                        idr mode: List
                        of the control bamfiles of
                        the spike-in, if --idrSpike is
                        True. Format is same. Give
                        full path
--idrControl {True,False}
                        idr mode: Should
                        include the control in IDR
                        peak calling (IDR mode).
                        Default is True.
--idrSpike {True,False}
                        idr mode: Should
                        include the spike-in in IDR
                        peak calling (IDR mode).
                        Default is True
--idrStyle {factor,histone}
                        idr mode: narrow
                        or broad peaks (IDR mode).
                        Default is narrow (factor) or
                        broad (histone)You can give a
                        single style for all
                        experiments e.g. factor or
```

```
                        different style for each
                        experiments e.g.
                        factor;histone
  --idrOutput IDROUTPUT
                        idr mode: Prefix
                        of the output file for each
                        experiment (IDR mode) e.g.
                        idr_expr1;idr_Expr2
  --idrMethod {homer}
                        idr mode: Peak
                        calling method: homer
                        in IDR
  --overFiles OVERFILES
                        PeakComparison mode:
                        Input peak file for the
                        comparison. Give as CSV input
                        e.g. dir1/dir2/peak1.bed,dir1
                        /dir2/peak2.bed. If not give,
                        pipeline automatically
                        identify the IDR peaks or
                        peaks from the output folder.
                        Give the full path of file.
  --overDist OVERDIST   PeakComparison mode:
                        The distance between
                        peaks to be consider as
                        overlapping. Default is 400
                        base-pairs
  --compareInfile COMPAREINFILE
                        PeakComparison mode:
                        Input file for peak
                        comparsion. Prepare a file
                        with two column and write
                        each comparison in a single
                        line e.g. ___
                        sample1_condition1
                        sample1_condition2
                        sample2_condition1
                        sample2_condition2 _____. The
                        sample1_condition1 ... should
                        match with last column of
                        input.txt
  --rdPvalue RDPVALUE   PeakComparison mode:
                        p-value in the
                        differential homer peak
                        calling. Default is 0.0001
  --rdFoldChange RDFOLDCHANGE
                        PeakComparison mode:
                        Fold changes in the
                        differential homer peak
```

```
                        calling. Default is 4.0
--rdSize RDSIZE       PeakComparison mode:
                        size of region around
                        peak to count tags. Default
                        is 1000 base-pairs
--rdPeak RDPEAK       PeakComparison mode:
                        List of the peak files
                        for which differential peaks
                        will be calculated. For each
                        line of --compareInfile, you
                        can provide --rdPeak file as
                        like this: sample1_condition1
                        :sample1_condition2,sample2_c
                        ondition1:sample2_condition2.
                        If you want to compare only
                        for same peak file then use:
                        peak1:peak2, that's it. Leave
                        it, if you have already run
                        callPeak mode.
--rdOther RDOTHER     PeakComparison mode:
                        You can add additional
                        parameters here for peak
                        comparison. Provide it as
                        comma seprated value in large
                        bracket e.g [-xyz,xx,-yy,kk].
                        To find additional option
                        type: getDifferentialPeaks
                        --help in your computer or go
                        to HOMER website.
--annpeakFiles ANNPEAKFILES
                        UserAnnotation/annot
                        ation mode: in this mode
                        you can provide peaks which
                        can be annotated by user
                        provided annotations files
                        (--annFiles). Multiple peak
                        files can be given as comma
                        separated values.
--annFiles ANNFILES   UserAnnotation mode:
                        provide bed files of your
                        region of interest e.g.
                        h3k4me3, jun/fos motif
                        location etc. Users can
                        provide more than one
                        annotation bed files as comma
                        separated values e.g.
                        f1.bed,f2.bed
--annSize ANNSIZE     UserAnnotation mode:
                        maximum distance between
```

```
                        the boundary of peaks
                        (--annpeakFiles) and
                        annotation bed files
                        (annFiles). For each
                        annotation bed files provide
                        this maximum distance e.g.
                        for h3k4me3 500, for jun/fos
                        100 as 500,100
--annName ANNNAME     UserAnnotation mode:
                        give name of your
                        annotations e.g. give
                        h3k4me3,jun/fos for file
                        f1.bed,f2.bed
--annPrefix ANNPREFIX
                        UserAnnotation/annot
                        ation mode: prefix of the
                        output annotated files. Use
                        this option if peak
                        information is given by users
                        by --annpeakFiles
--covSpike {True,False}
                        coverageTracks mode:
                        During calculation of the
                        whole genome coverage in
                        bins, reads should be
                        normalize according to
                        spike-in. This normalization
                        will be calculated within the
                        samples given in the
                        --inputfile. Default is False
--covSpike_NormalizationFormula {1,2}
                        coverageTracks mode:
                        To normalize coverage
                        tracks with spike-in, choose
                        formula among these. Suppose,
                        x1 ... xn is spike-in reads
                        per human reads in sample s1
                        ... sn. Then, per human read
                        spike-in reads will be: x1/s1,
                        ... xn/sn. Suppose this ratio
                        is r1 .. rn, then scaling
                        factor is (1)
                        min(r1...rn)/r1...
                        min(r1..rn)/rn and (2)
                        0.05/r1 ... 0.05/rn. Default
                        is 1. Notice that if you use
                        method 1, you can compare
                        coverage within given set of
                        samples only. If you want to
```

```
                        compare new samples, then you
                        have to generate coverage
                        file again.
  --covOtherOptions COVOTHEROPTIONS
                        coverageTracks mode:
                        Pipeline uses all default
                        parameters. If you want to
                        change something, besides
                        --bl, --effectiveGenomeSize
                        and -p, the other options of
                        bamCoverage (deepTools) can
                        be provided here as comma
                        separated values but in
                        bracket.
  --covExprType COVEXPRTYPE
                        coverageTracks mode:
                        Specify if you experiment
                        is greenCUT&RUN or CUT&RUN.
                        Use gCR for greenCUT&RUN and
                        CR for CUT&RUN
  --cMotif {True,False}
                        cutfrequency mode:
                        cutfrequency mode needs
                        location of the motifs in the
                        whole genome If you do not
                        have this file then create it
                        by using --cMotif True
  --cMotifFile CMOTIFFILE
                        cutfrequency mode:
                        If the --cMotif is False,
                        then give the file which
                        contains the location of the
                        motifs in the whole genome.
                        It should be in HOMER bed
                        file format
  --cMaN CMAN         cutfrequency mode:
                        Provide the MA number of
                        the JASPER motif Go to
                        http://jaspar.genereg.net/
                        for finding this number e.g.
                        for TP53 number is MA0106.
  --cGVersion CGVERSION
                        cutfrequency/annotat
                        ion/PeakComparison: In
                        the cutfrequency mode, if
                        --cMotif is true, provide the
                        version of the genome, so
                        that location of the motifs
                        can be generated. This one is
```

```
                          also require in the
                          annotation mode if you are
                          using other species than
                          human. Specify here. For
                          mouse use mm10.
  --cCenter CCENTER     cutfrequency mode:
                          Give the center of the
                          motif
  --initHeatmapOtherOptions INITHEATMAPOTHEROPTIONS
                          initHeatmap mode:
                          Pipeline uses all default
                          parameters. If you want to
                          change something, besides
                          --bl, --effectiveGenomeSize
                          and -p, the other options of
                          bamCompare (deepTools) can be
                          provided here as comma
                          separated values in large
                          bracket eg. [-x,xyz,-a,abc].
  --hSpike {True,False}
                          initHeatmap mode:
                          Should include spike in
                          preparation of bamcompare
                          files. Default is True
  --hMOpt HMOPT         heatmap mode:
                          Pipeline uses all default
                          parameters. If you want to
                          change something, besides
                          --missingDataAsZero,-bl,--
                          smartLabels,-p,--metagene,--
                          samplesLabel, the other
                          options of computeMatrix
                          (deepTools) can be provided
                          here as comma separated
                          values.(moreover, by default
                          -a 5000, -b 5000 are used. It
                          can be changed here.)
  --hPOpt HPOPT         heatmap mode:
                          Pipeline uses all default
                          parameters. If you want to
                          change something, besides
                          --refPointLabel and --dpi,
                          the other options of
                          plotHeatmap (deepTools) can
                          be provided here as comma
                          separated values.(moreover,
                          by default--colorMap GnBu are
                          used. It can be changed
                          here.)
```

```
--hCovComp {compare,coverage,NA}
                        heatmap mode:
                        Heatmap should be plotted
                        for bamcoverage (spike-in
                        normalized) or bamcompare
                        files? Leave it, if you want
                        to given your own files using
                        --hInFiles
--hCovMethod {1,2,NA}
                        heatmap mode: If
                        --hCovComp is bamcoverage,
                        then either you (1) can use
                        spike-in normalize bamcoverage
                        file or (2) simply a
                        bamcoverage file which will
                        be normalized during heatmap
                        production using following
                        formula: sum of reads within
                        the bins / (spike-in
                        reads/10,000). Default is 1.
                        But I recommend to use 2.
                        Leave it, if you want to
                        given your own files using
                        --hInFiles
--hInCounts HINCOUNTS
                        heatmap mode: If
                        you want to given your own
                        files using --hInFiles and
                        want to normalize it also,
                        then provide normalization
                        count for each samples in
                        comma separated value format.
                        It is basically spike-in-in-
                        sample-i/10,000. and use
                        --hCovMethod 2.
--hInFiles HINFILES   heatmap mode:
                        Input bamcoverage or
                        bamcompare files in csv
                        format. If file is not given,
                        tool will indentify files
                        from the --inputfile
--hInNames HINNAMES   heatmap mode:
                        Name of the samples in
                        csv format. Equal to the
                        number of files in
                        --hInFiles.
--hRegionMode {tss,metagene,bed,peaks}
                        heatmap mode:
                        mode of the region? on
```

```
                        tss, on given peak file or
                        select automatically total
                        peak file or differential
                        peaks from Peak folder or
                        metagene. Default is peaks
                        mode.
  --hGtf HGTF         heatmap mode:
                        Path of the GTF file, if
                        --hRegionMode is tss or
                        metagene
  --hBed HBED         heatmap mode:
                        List of bed files on
                        which you want to generate
                        heatmap in csv format, if
                        --hRegionMode bed
  --hPeakType {narrow-homer,broad-homer,all-homer,
                        seacr.stringent,seacr.relaxed}
                        heatmap mode:
                        Specify the type of peak
                        if --hRegionMode is peak. It
                        will help pipeline to find
                        and choose *-narrow*homer/*-
                        broad*homer/*-
                        all*homer/../*all*seacr*.Clea
                        n.bed file. Default is to use
                        narrow peaks of HOMER.
  --hDiffPeaks {True,False}
                        heatmap mode: In
                        the --hRegionMode peaks,
                        should program consider
                        differential peaks also ?
                        Default is the True
  --lProt LPROT       piggyBack mode:
                        Files with name of the
                        proteins per line. These are
                        those proteins which are
                        significantly enriched in the
                        mass spectrometry experiment
                        and you want to check if
                        these proteins are providing
                        any piggy-back binding to
                        your protein of interest. You
                        can find the list of proteins
                        on basis of the stochiometry
                        (iBaq values) observed in
                        your MassSpectrometry
                        experiment. This mode has
                        limitation because it can
                        predict the piggy-back
```

```
                         binding events only for those
                         proteins which are binding to
                         the DNA (not histone
                         modifications proteins) and
                         their PWM are known. This
                         list should not contain any
                         special character e.g. ; or -
                         or :
--mPwm MPWM            piggyBack and
                         doughnut mode: Provide
                         the MA number of the JASPER
                         motif Go to
                         http://jaspar.genereg.net/
                         for finding this number e.g.
                         for TP53 number is MA0106.
                         You can provide more than 1
                         motifs as comma separated
                         values.
--sProt SPROT          piggyBack and
                       doughnut mode: provide
                         the sequence of protein of
                         interest for which you are
                         searching piggy-back binding
                         event e.g. for NFYA you can
                         give CCAAT, for JUN you can
                         give TGANTCA, for ATF7 you
                         can give TGANNTCA. You can
                         provide more than one
                         sequence using comma
                         separated values. In the
                         sequence IUPAC codes are also
                         applicable.
--mPeak MPEAK          piggyBack: Path
                         of the peak file of greenPipe
                         experiment in bed format. It
                         is optional. If not given,
                         tool will automatically
                         search peak file on basis of
                         your --outputdir and
                         --inputfile. If provided give
                         the full path of peaks as
                         comma-separated values e.g. /
                         home/xyz/exp1.Clean.bed,/home
                         /xyz/exp2.Clean.bed.
--mDist MDIST          piggyBack and
                         doughnut mode: Search
                         motifs from this distance
                         from the centre of peaks.
                         Default is 400
```

```
--distPiggy DISTPIGGY
                        piggyBack and
                        doughnut mode: Distance
                        to find piggy back binding
                        events. Default is 400
--Species {human,mouse,rat,fruitfly,nematode,zebrafish,
           thale-cress,frog,pig}
                        piggyBack and
                        doughnut mode: Taxon ID
                        or name of Species according
                        to NDBI e.g. for home sapiens
                        taxon ID is 9606 and name is
                        human
--sFasta SFASTA       piggyBack/doughnut/a
                        nnotation/PeakComparison
                        mode: Fasta file of the
                        species genome
--gVer GVER           piggyBack and
                        doughnut mode: Genome
                        version of the fasta file.
                        Default is hg38
--mPvalue MPVALUE     piggyBack mode:
                        Motif finding pvalue for
                        piggy back binding events.
                        Default is 0.05
--sDist SDIST         piggyBack and
                        doughnut mode: Search
                        sequence of the motif from
                        the center of peak. Default
                        is 200. best practice:
                        --sDist should be half of the
                        --mDist
--mPrefix MPREFIX     piggyBack mode:
                        If giving your own bed
                        peak files using --mPeak then
                        give prefix of outputfor each
                        bedfiles
--dPeakfiles DPEAKFILES
                        doughnut mode:
                        Path of the peak file for
                        doughnut mode in bed format
                        as comma-separated values
                        e.g. /home/xyz/exp1.Clean.bed
                        ,/home/xyz/exp2.Clean.bed.
--dNames DNAMES       doughnut mode:
                        name of the experiment
                        for each bed file given with
                        --dPeakfiles e.g. exp1,exp2.
```

```
required arguments
______________________:
--modes MODES         run mode.
                        Multiple modes can be
                        provided as comma separated
                        values e.g. --modes
                        qc,alignment. Choices are:
                        qc, alignment,
                        equalRead, qcExperiment,
                        contamination,
                        initPeakCalling,
                        qcTagDirectories, callPeaks,
                        idr, doughnut,
                        PeakComparison, annotation,
                        UserAnnotation,
                        coverageTracks, cutfrequency,
                        initHeatmap, heatmap,
                        piggyBack,
--outputdir OUTPUTDIR
                        output directory (provide
                        full path)

common arguments
______________________:
--inputdir INPUTDIR   input directory having fastq
                        files (provide full path)
--inputfile INPUTFILE
                        input_file/sample_sheet in
                        .txt format
--libraryType {single,pair}
                        type of the library
--threads THREADS     number of threads (default
                        is: total CPU - 2)

Authors: (a) Sheikh Nizamuddin:
snizam001@gmail.com, (b) H.T. Marc Timmers:
m.timmers@dkfz-heidelberg.de
```
